# Review of the genus *Endothyrella* Zilch, 1960 with description of five new species (Gastropoda, Pulmonata, Plectopylidae)

**DOI:** 10.3897/zookeys.529.6139

**Published:** 2015-10-26

**Authors:** Barna Páll-Gergely, Prem B. Budha, Fred Naggs, Thierry Backeljau, Takahiro Asami

**Affiliations:** 1 Department of Biology, Shinshu University, Matsumoto 390-8621, Japan; 2 Central Department of Zoology, Tribhuvan University, Kirtipur, Kathmandu, Nepal; 3 Natural History Museum, Cromwell Road London, SW7 5BD, UK; 4 Royal Belgian Institute of Natural Sciences, Vautierstraat 29, B-1000 Brussels, Belgium; 5 University of Antwerp, Evolutionary Ecology Group Groenenborgerlaan 171, B-2020 Antwerp, Belgium

**Keywords:** Taxonomy, anatomy, India, Nepal, China, Myanmar, sinistrality

## Abstract

All known taxa of the genus *Endothyrella* Zilch, 1960 (family Plectopylidae) are reviewed. Altogether 23 *Endothyrella* species are recognized. All species are illustrated and whenever possible, photographs of the available type specimens are provided. Five new species are described: *Endothyrella angulata* Budha & Páll-Gergely, **sp. n.**, *Endothyrella dolakhaensis* Budha & Páll-Gergely, **sp. n.** and *Endothyrella nepalica* Budha & Páll-Gergely, **sp. n.** from Nepal, *Endothyrella robustistriata* Páll-Gergely, **sp. n.** from the Naga Hills, India, and *Endothyrella inexpectata* Páll-Gergely, **sp. n.** from Sichuan, China. Helix (Plectopylis) munipurensis Godwin-Austen, 1875 is synonymized with Helix (Plectopylis) serica Godwin-Austen, 1875, and Plectopylis (Endothyra) gregorsoni Gude, 1915 is synonymized with Helix (Plectopylis) macromphalus W. Blanford, 1870. Plectopylis plectostoma var. exerta Gude, 1901 is a synonym of Plectopylis plectostoma var. tricarinata Gude, 1896, which is a species in its own right. Five species of the genus *Chersaecia* viz. Plectopylis (Chersaecia) bedfordi Gude, 1915, Helix (Plectopylis) brahma Godwin-Austen, 1879, Helix (Plectopylis) Oglei Godwin-Austen, 1879, Helix (Plectopylis) serica Godwin-Austen, 1875, and Plectopylis (Endothyra) williamsoni Gude, 1915 are moved to *Endothyrella*. The holotype of *Plectopylis hanleyi* Godwin-Austen, 1879 seems to be lost; therefore, *Plectopylis hanleyi* is considered to be a *nomen dubium*.

## Introduction

The Plectopylidae Möllendorff, 1898 are a land snail family of the superfamily Plectopyloidea that ranges across large parts of southeast Asia from Nepal to southern Japan (Gude 1899d, Páll-Gergely and Hunyadi 2013). Schileyko (1999) classified two families in the Plectopyloidea: the Plectopylidae and the mainly Sri Lankan Corillidae Pilsbry, 1905. Other authors (Zilch 1960, Bouchet and Rocroi 2005) also included the African Sculptariidae Degner, 1923 in the superfamily. Historically, the family name Corillidae (e.g. Yen 1939 and Zilch 1960) or the helicid subfamily Corillinae (in Gude 1914b) have been applied to the current concept of Plectopyloidea. The Chinese *Amphicoelina* Haas, 1933 has been included in the Corillidae or the Plectopylidae by Yen (1939), Zilch (1960) and Schileyko (1999). That genus, however, likely belongs to the Camaenidae (see Páll-Gergely and Asami 2014), as originally proposed by Haas (1933). The Plectopylidae differ from the Corillidae by the presence of one or two vertical (= perpendicular to the suture) lamellae on the parietal wall, approximately a quarter to a half whorl behind the aperture. In contrast, the Corillidae have only horizontal (= parallel with the suture) parietal plicae (in *Corilla* all plicae may be absent).

Gude revised every known taxon of *Plectopylis* Benson, 1860 at the end of the 19^th^ century, and published drawings of their shells and armature (lamella complex) (see citations in Richardson 1986). He subdivided *Plectopylis* into seven “sections” (Gude 1899c): *Endothyra* Gude, 1899c, *Chersaecia* Gude, 1899c, *Endoplon* Gude, 1899c, *Plectopylis*, *Sinicola* Gude, 1899c, *Enteroplax* Gude, 1899d and *Sykesia* Gude, 1897f. *Enteroplax* was transferred to the Strobilopsidae Wenz, 1915 (Solem 1968, Schileyko 1998), and *Ruthvenia* Gude, 1911 (replacement name for *Sykesia* which itself was a replacement name for *Austenia* Gude, 1897e) to the Endodontidae Pilsbry, 1895 (Gude 1914b, Schileyko 2001) or to the Charopidae Hutton, 1884 (Schileyko 2010, Raheem et al. 2014). The name *Endothyrella* was established by Zilch (1960) to replace *Endothyra* Gude, 1899, a junior homonym of *Endothyra* Phillips, 1845 (Foraminifera).

Gude’s (1899c) diagnoses of his sections are based on the direction of the coiling of the shell, the depth of the umbilicus, and the morphology and direction of the palatal folds. Most of his diagnoses are not mutually exclusive. Recent revisions of the genera *Endoplon* and *Sinicola* (Páll-Gergely and Hunyadi 2013, Páll-Gergely and Asami 2014, and Páll-Gergely et al. 2015) showed that the species assigned to these two genera should be classified within multiple genera and the genera should be re-diagnosed. Moreover, several species were misassigned by Gude (1899c), which was probably the result of focusing exclusively on the morphology of the parietal plicae.

The aim of this paper is to review and diagnose all *Endothyrella* species, publish images of the type specimens where possible, provide a diagnosis of *Endothyrella*, and delimit it from other plectopylid genera. Ongoing revision of the genera *Chersaecia* and *Plectopylis* revealed that *Chersaecia* sensu Gude (1899c, 1915) worked as a “garbage can” including species that could not be classified within other sections. Revising the validity of *Chersaecia* species is beyond the scope of the present paper. However, three sinistral (*bedfordi*, *brahma*, *williamsoni*) and two dextral (*oglei*, *serica*) species are moved from *Chersaecia* to *Endothyrella*, mainly based on the sculpture of the embryonic whorls and the absence of the apertural fold. Additionally, five new *Endothyrella* species are described from Nepal, India, and China.

## Taxonomic history of Endothyrella and Chersaecia species

*Endothyrella plectostoma* was the first described species that is currently placed in the Plectopylidae. It was introduced as *Helix plectostoma* by Benson (1836), who classified it within the subgenus *Helicodonta* and who mentioned that because of its angulated periphery it shows connection towards the subgenus *Helicigona*. In modern classifications *Helicodonta* and *Helicigona* belong to the families Helicodontidae and Helicidae, respectively, and both are the members of the superfamily Helicoidea (Schileyko 2006a, 2006b). Benson (1836) compared *Helix plectostoma* with *Helix personata*, (= *Isognomostoma isognomostomos* [Schröter, 1784], family Helicidae) and *Helix corcyrensis* (= *Lindholmiola corcyrensis* [Rossmässler, 1838], family Helicodontidae).

The helicid subgenus *Plectopylis* was erected by Benson (1860) for six species subdivided into three unnamed “sections”. His third section is equivalent with Gude’s (1899c) *Endothyra*, and contained *Helix plectostoma* and *Helix pinacis* Benson, 1859. Gude (1899c) diagnosed *Endothyra* as follows: “Sinistral. Umbilicus moderate. Palatal folds horizontal or oblique”. He selected *Helix plectostoma* as the type species and classified the following species in *Endothyra*: *minor* Godwin-Austen, 1879b, *hanleyi* Godwin-Austen, 1879b, *blanda* Gude, 1898, *macromphalus* W. Blanford, 1870, *sowerbyi* Gude, 1898, *plectostoma* Benson, 1836 (including *prodigium* Benson and *tricarinata* Gude, 1896), *affinis* Gude, 1897b, *pinacis* (including *pettos* von Martens, 1868), and *fultoni* Godwin-Austen, 1892.

Simultaneously, Gude (1899c) diagnosed the “section” *Chersaecia* as follows: “Sinistral or dextral. Umbilicus wide. Palatal folds horizontal or oblique. Sometimes with one oblique or vertical plate”. He selected *Plectopylis leiophis* Benson, 1860 as type species and classified the following species in *Chersaecia*: *muspratti* Gude, 1897, *austeni* Gude, 1899b, *oglei* Godwin-Austen, 1879a, *serica* Godwin-Austen, 1875, *munipurensis* Godwin-Austen, 1875, *nagaensis* Godwin-Austen, 1875, *pseudophis* “Blanford” in Godwin-Austen, 1875, *leiophis*, *refuga* Gould, 1846, *perrierae* Gude, 1897, *shiroiensis* Godwin-Austen, 1875, *perarcta* W. Blanford, 1865, *brachydiscus* Godwin-Austen, 1879a, *dextrorsa* Benson, 1860, *shanensis* Stoliczka, 1873, *brahma* Godwin-Austen, 1879a, *andersoni* W. Blanford, 1869, and *laomontana* Pfeiffer, 1863. An additional *Chersaecia* species (*Plectopylis kengtungensis* Gude, 1914a) was described later.

From the shells collected during the Abor Expedition (Abor Country, north-eastern India), Gude (1915) described seven *Plectopylis* species, classifying them in the subgenera *Endothyra* (*oakesi*, *gregorsoni*, *miriensis*), *Chersaecia* (*williamsoni* and *bedfordi*), *Endoplon (aborensis)* and Sinicola (babbagei). Gude (1915) apparently relied only on the morphology of the palatal plicae. In case of *williamsoni* and *bedfordi*, Gude (1915) mentioned that they are closely related to Plectopylis (Chersaecia) brahma. The subgeneric classification of *Plectopylis aborensis* is based on its palatal plicae, which resemble those of Plectopylis (Endoplon) brachyplecta, whereas those of *Plectopylis babbagei* resemble those of *Plectopylis pulvinaris*, a species classified within the subgenus *Sinicola* by Gude (1899c). After Gude (1915), only two new species were added to *Chersaecia*, viz. Plectopylis (Chersaecia) degerbolae Solem, 1966 and Plectopylis (Chersaecia) simplex Solem, 1966. *Plectopylis babbagei* and *Plectopylis aborensis* were moved to *Endothyrella* by Páll-Gergely and Hunyadi (2013).

## Material and methods

Shell whorls were counted according to Kerney and Cameron (1979: 13) (precision 0.25 mm). Differences in size are indicated in the diagnosis using the following terms: tiny (smaller than 6 mm), very small (6–10 mm), small (10–15 mm), medium-sized (15–20 mm), large (20–25 mm), very large (25–30 mm).

**Figure 1.**
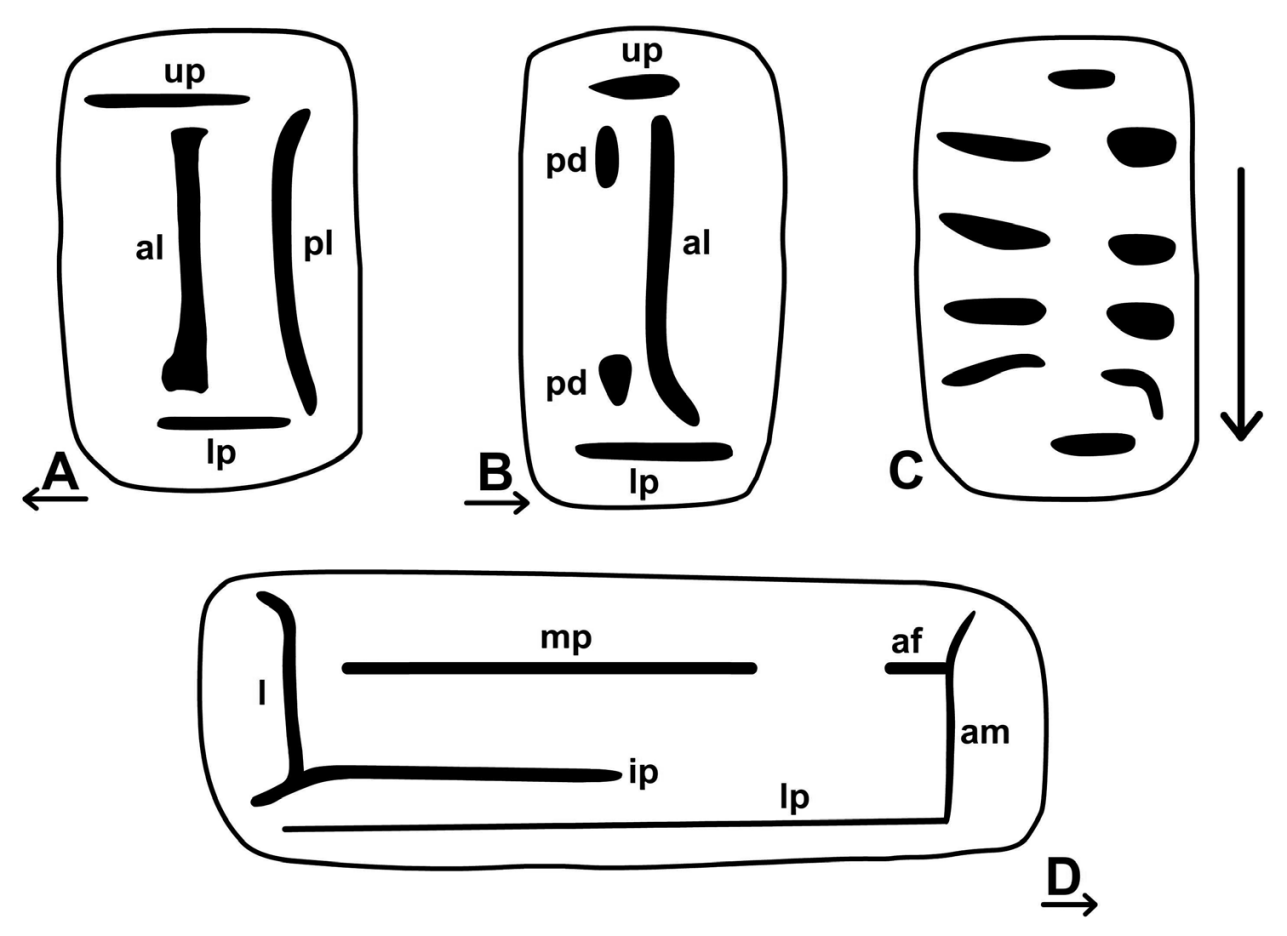
Nomenclature of parietal (**A, B, D**) and palatal (**C**) plicae and lamellae. **A** shows a “*Gudeodiscus*-type” plication with two lamellae **B** shows a usual *Endothyrella* lamellation **D** shows a “*Chersaecia*-type” lamellation with long lower plica and middle plica not connected to the apertural fold (in most species however, they are connected forming a continuous plica). Small arrows under the letters show the direction of the aperture (**A** shows dextral, **B** and **D** sinistral specimen). Large arrow next to **C** shows the direction of counting of palatal plicae (first above, last below). Abbreviations: af: apertural fold; al: anterior lamella; am: apertural margin (peristome); ip: intermediate plica; l: lamella; lp: lower plica; mp: main plica; pl: posterior lamella; pd: posterior denticles; pl: posterior lamella; up: upper plica. Note that there are upper and lower plicae on both (palatal and parietal) walls.

For the nomenclature of lamellae (vertical parietal folds) and plicae (horizontal parietal folds and palatal folds) see Figure 1. Whenever possible, the internal lamellae and plicae have been exposed by removing the shell wall at the appropriate part of the shells (inner view). Yet, if damaging the shells was not an option (because too few shells available), the plicae were figured on the basis of their visibility through the shell wall (outer view). “Anterior” refers to the part or side of the armature in direction of the aperture, “posterior” refers to the other side of the armature.

For each taxon, the specimens studied are listed separately as types, museum material and new material. Geographic names mentioned in the literature and on labels (Table 1) were searched using Google, Google Earth and Lozupone et al. (2004). Locality names are copies from the labels and from the literature with original spelling. Therefore the same locality might present with more than one spelling (e.g. Sikkim/Sikhim, Sadia/Sadiya, Khasi/Khasia).

**Table 1.** Geographic names mentioned in the literature and on labels of *Endothyrella* Zilch, 1960 taxa. Asterisks indicate names with unknown exact localities.

Locality	Region	Taxon
Abor Hills	India, Arunachal Pradesh	*williamsoni*
Arakan Hills (= Rakhine)	Myanmar, Rakhine district	*plectostoma*
Bassein (= Pathein)	Myanmar, Ayeyarwady district: 16°47'N, 94°44'E	*plectostoma*
Brahmakund	India, Assam: 27°51.4'N, 96°22'E	*brahma*
Burrail (= Barail) Gorge	India, mountain range centered 70 km NE Silchar (Assam)	*macromphalus*, *plectostoma*
Cachar (= Katchar)	India, District in Assam: 24°46'N, 92°50'E	*affinis*, *blanda*, *plectostoma*, *serica*
Cherra Poonjee (= Cherrapunji)	India, Meghalaya, Khasi Hills: 25°18'N, 91°42'E	*affinis*, *fultoni*, *plectostoma*, *tricarinata*
Chittagong	City in Bangladesh: 22°22'N 91°48'E; also name of a district	*plectostoma*
Damsang Peak	India, Sikkim	*blanda*, *pinacis*
Darjiling (= Darjeeling)	India, town in West Bengal: 27°2'N, 88°15.5'E	*blanda*, *macromphalus*, *minor*, *pinacis*, *plectostoma*
Dihang (= Siang) River	India, Arunachal Pradesh: river flows to the Brahmaputra at 27°50'N, 95°27'E	*bedfordi*, *oakesi*
Dunsiri (= Dhansiri) River	India, river flows to the Brahmaputra at 26°42'N, 93°35'E	*plectostoma*
Durrang (= Darrang)	India, district in Assam: centered at 26°26'N, 92°1.5'E	*plectostoma*
Garo Hills	India, Western Meghalaya: 25°28'N, 90°20'E	*plectostoma*
Ghoramara	Bangladesh, town approx. 15 km NW from Chittagong, at 22°29'N 91°43'E	*plectostoma*
Prov. Harenni*	Myanmar	*plectostoma*
Hengdan*	India, mountain in northern Cachar Hills	*serica*
Ihang River*	India, Manipur	*robustistriata* sp. n., *serica*
Japvo Peak	India, highest mountain in Naga Hills: 25°36'N, 94°4'E	*serica*
Karenni (= Kayah) State	state located south of Shan State, Myanmar	*plectostoma*
Khasi (= Khasia) Hills	India, Meghalaya	*affinis*, *fultoni*, *macromphalus*, *minor*, *pinacis* (?), *plectostoma*, *sowerbyi*, *tricarinata*
Khunho (= Khono) Mountain	India, Naga Hills: 25°31.3'N, 94°6.5'E	*serica*
Kohima	India, town in SE Nagaland: 25°40'N, 94°6.5'E	*serica*
Kopamedza ridge*	India, Dafla Hills, Barail Range	*serica*
Laisen Peak*	India, Manipur	*robustistriata* sp. n.
Lhota Naga*	India, Nagaland, Naga Hills	*robustistriata* sp. n.
Lopchu	India, Sikkim 27°7.5'N, 88°25'E	*pinacis*
Luyor Peak	India, Abor county, Arunachal Pradesh: 28°45'N, 95°45'E	*babbagei*
Mairung (= Mairang)	India, village in Meghalaya, Khasi Hills: 25°34.2'N, 91°37.8'E	*macromphalus*
Miri Hills	India, on the border between Assam and Arunachal Pradesh	*miriensis*
Munipur (= Manipur)	India, Manipur	*plectostoma*, *robustistriata* sp. n., *serica*
Naga Hills	on the border of Nagaland (India) and Myanmar	*blanda*, *macromphalus* (?), *minor* (?), *plectostoma*, *plectostoma*, *serica*
Naraindher*	India, Assam, Cachar district	*affinis*
Pankabari (= Pankhabari)	India, northern part of West Bengal: 26°50'N, 88°16'E	*pinacis*
Pegu (= Bago)	Myanmar, Bago District: 17°20'N, 96°29'E	*plectostoma*
Picholanulla*	India, probably Dafla Hills	*plectostoma*
Pyema Khyoung	Myanmar, Ayeyarwady district	*plectostoma*
Rarhichu (= Rungpo?)*	India, Sikkim	*blanda*, *minor*, *pinacis*
Renging (= Rengging)	India, Abor county, Arunachal Pradesh: 28°9'N, 95°15.5'E	*aborensis*
Richila (Rechila) Peak	India, Sikkim: 27°8'N, 88°45'E	*blanda*
Rinkpo valley*	India, Sikkim	*blanda*
Rissetchu*	India, Sikkim	*blanda*
Riu*	India, Abor Hills	*oakesi*
Rotung (= Rottung)	India, Abor county, Arunachal Pradesh: 28°8'N, 95°8.5'E	*aborensis*
Rungmaval*	India, Sikkim	*pinacis*
Rungun*	India, probably Sikkim	*minor*, *pinacis*
Sadia (Sadiya)	India, Assam: 27°51.6'N, 95°37.6'E	*oglei*
Salwen (= Salween) River	River in China and eastern Myanmar	*plectostoma*
Shillong	India, city in Meghalaya, Khasi Hills	*macromphalus*
Shiroifurar peak (probably Shirui Hills)	India, NE Manipur: 25°6.3'N 94°27.4'E	*plectostoma*
Shweego (probably Shwegu)	probably Myanmar, Kachin District: 24°12'N, 96°48'E	*plectostoma*
Sibbum (= Sibum)	India, Abor Hills: 28°19'N, 95°9'E	*oakesi*
Sigon (= Siyom) River	India, river runs into the Siang River at 28°14'N, 95°E	*bedfordi*
Singging*	India, Abor Hills	*oakesi*
Sylhet	Bangladesh, Sylhet Division, Sylhet city: 24°54'N, 91°52'E	*plectostoma*, *serica*
Teria Ghat	India, Khasi Hills	*macromphalus*, *plectostoma*
Tongoop*	Myanmar, Rakhine district	*plectostoma*
Torúpútú*	India, Dafla Hills	*robustistriata* sp. n.
Tsanspu (= Tsangpo) River	India (Tibetan name of the Brahmaputra River)	*bedfordi*
Yamne River	India, Abor Hills, river flows into the Siang River at 28°10.5'N, 95°13'E	*gregorsoni*, *oakesi*
Ywathit (= Ywarthit)	Village (?) in Kayah State, Myanmar: 19°10'N 97°30'E	*plectostoma*

Ethanol-preserved specimens were dissected under a Leica stereomicroscope, equipped with a photographic camera. In description of the reproductive system, we used the terms “proximal” and “distal” relative to the centre of the body.

Individual buccal masses was removed and soaked in 2 M KOH solution for 5 h before extracting the radula, which was preserved in 70% ethanol. Radulae and shells were directly observed without coating under a low vacuum SEM (Miniscope TM-1000, Hitachi High-Technologies, Tokyo).

The dates of publication of the Proceedings of the Zoological Society of London follows Duncan (1937).

### Taxonomic treatment

All available type material of each *Chersaecia* taxon deposited in the MCZ, NHMUK, SMF and ZMUC have been examined. The type specimens of *Endothyrella* taxa examined are mentioned under each species.

The following shell characters of species formerly classified in *Chersaecia* and *Endothyrella* were examined in order to revise the generic assignment and diagnose genera: (1) coiling direction; (2) sculpture of the protoconch; (3) presence or absence of the apertural fold; morphology of the parietal plicae and lamellae, namely (4) the presence/absence/length of a horizontal main plica, (5) the presence/absence/length of a the lower plica, and (6) the presence or absence of additional denticles behind the lamella; (7) morphology of middle palatal plicae (the first and last are straight in almost all cases); and the (8) presence/absence/morphology of hairs.

This taxonomic revision of *Endothyrella* species is based on morphology by examination of specimens and literature. The present species are defined based on unique combinations of morphological traits, some of which are discrete in nature (e.g. presence or absence of periostracal filaments) or continuous but with distinct gaps (e.g. height of the spire). No specimens were found that show transitional characters between probably sympatric morphospecies (Table 2). Although we have no, or too little, information on the genetic, physiological and/or ecological basis of the phenotypic characters used to describe the species in this work, we putatively interpret the diagnostic phenotypic differences under the biological species concept (Mayr 1942), i.e. as markers of reproductive isolation. However, the biological species concept is not applicable to specific identification of allopatric populations regardless of their morphological differences. The shell shape, the characters of the armature and the shell sculpture was of primary important in recognizing allopatric species. In some cases we found stable but minor differences of allopatric populations, such as the divided/not palatal plicae in *Plectopylis macromphalus* and not divided ones in *Plectopylis gregorsoni* and the difference in the spaces between hair rows in Nepalese versus Indian populations of *Endothyrella minor*. In those cases we handled these forms under a single specific name. No subspecific differentiation is applied because most samples are provided with poor locality data which, in most cases, does not provide a clear understanding on the distribution of certain morphological forms.

**Table 2.** Co-occurrence of *Endothyrella* Zilch, 1960 species. Two stars indicate that the two species were collected at geographically close sites (*nepalica*-*minor*: 2680 m). One star indicates presence of the two species mixed within museum samples.

	*nepalica* sp. n.	*blanda*	*macromphalus*	*tricarinata*	*sowerbyi*	*affinis*
***affinis***		*	*			
***minor***	**	*	*			
***pinacis***		*				
***blanda***			*		*	
***plectostoma***		*		*	*	*

### Abbreviations

CDZMTU Central Department Zoology Museum of Tribhuvan University (Kathmandu, Nepal)

D shell diameter

H shell height

HA Collection András Hunyadi (Budapest, Hungary)

HNHM Hungarian Natural History Museum (Budapest, Hungary)

JUO Collection Jamen Uiriamu Otani (Osaka, Japan)

MCZ Museum of Comparative Zoology (Massachusetts, USA)

MMGY Mátra Múzeum, Gyöngyös, Hungary

NHM The Natural History Museum (London, UK)

NHMSB Natural History Museum, Sibiu (Romania), Bielz collection

NHMUK When citing NHM registered specimens

NHMW Naturhistorisches Museum Wien (Vienna, Austria)

NMBE Natural History Museum, Bern, Switzerland

SMF Senckenberg Forschungsinstitut und Naturmuseum (Frankfurt am Main, Germany)

TH Collection Takashi Hosoda (Kofu, Japan)

UMZC University Museum of Zoology (Cambridge, UK)

Wh number of whorls

WM Collection Wim J. M. Maassen (Echt, The Netherlands)

ZMB/MOLL Museum für Naturkunde (Berlin, Germany)

ZMH University of Hamburg (Hamburg, Germany)

ZMUC Zoological Museum, University of Copenhagen (Denmark)

ZSI Zoological Survey of India

## Systematic Part

### Family Plectopylidae Möllendorff, 1898

#### Genus Chersaecia

Taxon classificationAnimaliaPulmonataPlectopylidae

Gude, 1899

Chersaecia 1899c Chersaecia (section of the genus *Plectopylis*) Gude: Science Gossip, 6: 148.
Chersaecia 1999 *Chersaecia*, — Schileyko: Treatise on Recent Terrestrial Pulmonate Molluscs, Part 4. (...): 2: 462.

##### Type species.

**Figure 2.**
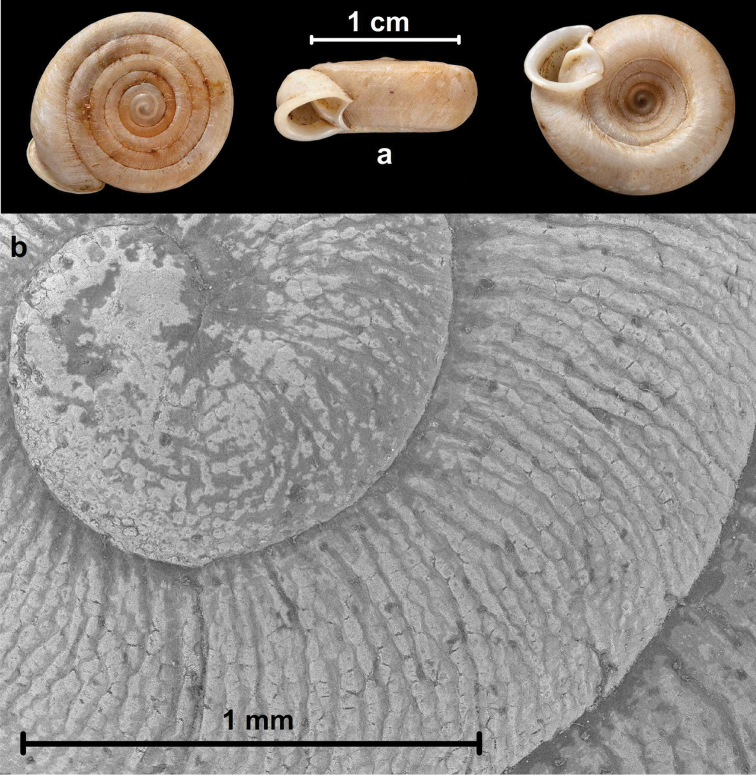
Shell (**A**) and protoconch (**B**) of *Chersaecia leiophis* (Benson, 1860), Akouktoung, purchased of W. Theobald Esq., NHMUK 1888.12.4.1526–1528 (two different shells). Photos: H. Taylor (**A**) and B. Páll-Gergely (**B**).

Helix (Plectopylis) Leiophis Benson, 1860 (Figure 2) by original designation.

##### Diagnosis.

Shell sinistral or dextral, flat, widely umbilicated; in most cases protoconch seemingly “smooth” to the naked eye, but not glossy, rather matt; under the microscope usually tubercles of various size are visible (Figure 2B); sometimes the tuberculated protoconch is irregularly wrinkled; flat periostracal filaments are visible on the body whorl or on the dorsal surface in only a few species; aperture always with a fold; parietal wall with one vertical lamella and usually one or two long horizontal plicae (main plica and lower plica) reaching the callus; palatal plicae horizontal, sometimes divided in the middle, in some species with several additional denticles posteriorly, in some species similar to that of *Plectopylis* (three horizontal plicae above and one below the vertical plate formed by the accretion of two plicae).

Only one *Chersaecia* species is known anatomically (*Chersaecia simplex* in the original description: Solem 1966). Penis internally with approximately eight longitudinal rows, those situated next to the vas deferens are distinctly larger; vas deferens becomes a part of the penis wall at the penioviducal angle; no epiphallic differentiation observed; retractor muscle inserts on the dorsal surface of the penis and attaches to the diaphragm; diverticulum absent, gametolytic sac long and thickened.

##### Differential diagnosis.

*Chersaecia* differs from *Endothyrella*, *Gudeodiscus* Páll-Gergely, 2013, *Halongella* Páll-Gergely, 2013, *Sicradiscus* Páll-Gergely, 2013 and *Sinicola* by the usually tuberculated (not regularly ribbed) protoconch. The presence of long parietal plicae (main and lower) distinguishes most *Chersaecia* species from most *Endothyrella*, *Gudeodiscus*, *Halongella*, *Sicradiscus* and *Sinicola* species. The delimitation of *Chersaecia* from *Plectopylis* and *Endoplon* needs further investigation. Among all plectopylids examined to date *Chersaecia simplex* is the only species found to lack an epiphallus. The anatomy of more *Chersaecia* species should be studied to check the taxonomic value of the lack of the epiphallus.

##### Content.

*austeni*, *brachydiscus*, *degerbolae*, *dextrorsa*, *kengtungensis*, *leiophis* (*pseudophis* is probably a synonym, see Gude 1908a), *muspratti*, *nagaensis*, *perarcta*, *perrierae*, *refuga*, *shanensis*, *shiroiensis*, *simplex*.

##### Distribution.

The genus is known to inhabit northeastern India, eastern and southern Myanmar (Burma) and northern Thailand.

#### Genus Endothyrella

Taxon classificationAnimaliaPulmonataPlectopylidae

Zilch, 1960

Endothyrella 1899c Endothyra (section of the genus *Plectopylis*) Gude: Science Gossip, 6: 148., non *Endothyra* Phillips, 1845 (Foraminifera).
Endothyrella 1960 Plectopylis (Endothyrella), — Zilch: Handbuch der Paläozoologie, 6 (2).
Endothyrella 1999 *Endothyrella*, — Schileyko: Treatise on Recent Terrestrial Pulmonate Molluscs, Part 4.(...): 2: 460.

##### Type species.

*Helix plectostoma* Benson, 1836, by original designation.

##### Diagnosis.

Shell sinistral or dextral; protoconch usually finely, regularly ribbed (see also discussion and Figures 6A–F); periostracal folds usually present on the body whorl; they are arranged in 3–7 lines; folds hair-like in most species, resulting from the rolling of flat folds; folds flat (not rolled) in some species only (see Figures 8D, 20A–F); dorsal sculpture strong, usually reticulated (both radial and spiral lines present, see Figure 8A); umbilicus wide to narrow; body whorl rounded in some species but rather bluntly shouldered (keeled) in others; apertural fold always absent; main plica usually absent (present in a few species only); low plica (if present) runs close to and parallel with the lower suture, it is usually very short (present only under the lamella), but in some species it reaches the callus; parietal wall with a single lamella with denticles posteriorly (probably homologous with the posterior lamella); two lamellae were reported in one species (*Endothyrella aborensis*) only; palatal plicae complicated in most species with many small denticles at their posterior ends; in many species they are at least party divided in the middle.

Genitalia (see Figures 18, 21, 22B–F, 25, 26): The left ommatophoral retractor passes between penis and vagina (in sinistral species). Penis internally with hollows (small pocket-like structures) having calcareous granules inside; penial papilla absent; epiphallus may be longer than penis and enters penis laterally; epiphallus with longitudinal folds internally; small penial caecum usually present at the penis-epiphallus boundary; retractor muscle inserts on the caecum and attaches to the diaphragm; diverticulum (if present) and gametolytic sac are of the same size.

Radula (see Figures 19A–F): Central tooth larger than the ectocones of the first laterals; marginals tricuspid (= ectocones are divided) or even quadricuspid (both the endocones and ectocones are divided); the incision between the ectocones and endocones usually deep (*Endothyrella fultoni* has rhomboid marginals which are unique in the whole family).

##### Differential diagnosis.

All known species of the genera *Sinicola*, *Gudeodiscus*, *Halongella* and *Sicradiscus* are dextral. Regardless of the coiling direction, most *Endothyrella* species differ from *Sinicola* by the presence of usually hair-like periostracal folds standing in multiple lines. Deciduous periostracal folds in *Sinicola* are present only along the keel and the folds are always flat. Most *Sinicola* species (especially the large species) have a sharp keel, whereas *Endothyrella* species usually have a rounded or slightly keeled, shouldered body whorl. The palatal plicae of *Sinicola* are usually simple, horizontal, straight and parallel, but in *Endothyrella* they are often oblique to vertical, divided and ornamented with minute denticles at their posterior ends. In *Sinicola* the posterior lamella is present on the parietal wall, with two horizontal plicae anteriorly above and below, whereas in most *Endothyrella* species (probably except for *Endothyrella aborensis*) the anterior lamella is present and the posterior is missing or reduced to one or two short vertical plicae.

Some *Gudeodiscus* and *Halongella* species possess low, radial periostracal folds (e.g. Páll-Gergely et al. 2015, fig. 10e–f), similar to those of *Endothyrella nepalica* sp. n. (see there). The radial folds have serrated edges in *Gudeodiscus phlyarius* (Mabille, 1887). The tiny tips of the serrated folds seem to occur in a spiralling pattern (see Páll-Gergely and Hunyadi 2013, fig. 113 and Páll-Gergely et al. 2015, fig. 10c–d). All of these periostracal features of *Gudeodiscus* and *Halongella* are, however, easily distinguishable from the long, hair-like folds of the genus *Endothyrella*.

Some *Gudeodiscus* species possess a fold in the aperture, which is always missing in *Endothyrella*. The palatal plicae in *Gudeodiscus* are usually depressed Z- or L-shaped and posterior small denticles are very rare (except for one denticle above the posterior end of the last plica), whereas the palatal plicae of *Endothyrella* are frequently divided in the middle and posterior small denticles are usually present. In *Endothyrella* the anterior lamella is present, and often the upper horizontal plica is missing, whereas in *Gudeodiscus* both lamellae, or only the posterior one, are visible and the upper horizontal plica (above the lamella) is almost always present. Additionally, *Gudeodiscus* species have a rounded body whorl, while in many *Endothyrella* species the body whorl is angled or shouldered. Our limited knowledge on the anatomy of *Endothyrella* species shows that the entire inner penial wall of *Endothyrella* is covered by pits, whereas in *Gudeodiscus* these pocket-like structures are restricted to the a certain (usually apical) portion of the penis.

*Sicradiscus* is similar to *Endothyrella* in possessing a weak or reduced posterior lamella. Long periostracal folds standing in more than one row have also been found in one *Sicradiscus* species, namely in juveniles of *Sicradiscus transitus* Páll-Gergely, 2013. This species, however, has hairs standing in two spiral lines on the body whorl, whereas in *Endothyrella* the hairs are arranged in 3–7 spiral lines. This trait seems to be absent in adult *Sicradiscus transitus* shells and all other species of *Sicradiscus*, but is common in fully grown *Endothyrella* shells (i.e. most species possess them). The two genera (i.e. *Endothyrella* and *Sicradiscus*) differ in the short, straight palatal plicae, which are usually connected in *Sicradiscus* vs. longer, more complex palatal plicae sometimes having additional denticles in *Endothyrella*. In both genera divided plicae may occur, but in the case of *Sicradiscus* the posterior fourth and fifth plicae seem to be always connected, whereas in *Endothyrella* all plicae are free. Moreover, western *Sicradiscus* species (*feheri* Páll-Gergely, 2013, *invius* [Heude, 1885], *mansuyi* [Gude, 1908b], *securus* [Heude, 1885] and *transitus*) differ from *Endothyrella* by the presence of a strong apertural fold.

*Plectopylis* and *Endoplon* species have a granulated or smooth protoconch, whereas it is usually finely ribbed in *Endothyrella*. Moreover, *Plectopylis* and *Endoplon* usually have a strong apertural fold which is often connected to a long main plica. In contrast, although some *Endothyrella* species have a main plica, they all lack an apertural fold. See also under *Chersaecia* and Table 3.

**Table 3.** Characters of the plectopylid genera possessing ribbed protoconchs.

Genus	Coiling direction	Apertural fold	Lower plica	Body whorl	Anterior lamella	Posterior lamella	Periostracal folds	Penial pockets
*Endothyrella*	sinistral or dextral	absent	short or long (reaching peristome)	rounded or keeled	present	present (?), absent or reduced	usually in multiple rows	whole penial wall
*Sinicola*	dextral	absent (present in 1 species)	short	keeled	absent or reduced	present	present in a single row or absent	whole penial wall
*Gudeodiscus*	dextral	absent or present	missing or short	rounded	present, reduced or absent	present	absent	apical part
*Halongella*	dextral	present	short	rounded	present, reduced or absent	present	absent	whole penial wall
eastern *Sicradiscus*	dextral	absent	missing or short	keeled	present	present or reduced	present in a single row or absent	whole penial wall
western *Sicradiscus*	dextral	present	missing or short	rounded	present	present or reduced	absent	apical part

##### Content.

*aborensis*, *affinis*, *angulata* sp. n., *babbagei*, *bedfordi*, *blanda*, *brahma*, *dolakhaensis* sp. n., *fultoni*, *inexpectata* sp. n., *macromphalus* (syn.: *gregorsoni*), *minor*, *miriensis*, *nepalica* sp. n., *oakesi*, *oglei*, *pinacis*, *plectostoma*, *robustistriata* sp. n., *serica* (syn: *munipurensis*), *sowerbyi*, *tricarinata*, *williamsoni*. See also Tables 4 and 5.

**Table 4.** (Sub)generic classification of *Endothyrella* Zilch, 1960 (formerly *Endothyra* Gude, 1899) species by previous authors. Species marked with a star were moved to *Endothyrella* by Páll-Gergely & Hunyadi (2013). Abbreviations: n. m.: not mentioned.

Name	Subgenus in Gude (1899c)	Subgenus in Gude (1915)
*aborensis* *		*Endoplon*
*affinis*	*Endothyra*	n. m.
*babbagei* *		*Sinicola*
*bedfordi*		*Chersaecia*
*blanda*	*Endothyra*	n. m.
*brahma*	*Chersaecia*	*Chersaecia*
*exerta* (syn. of *tricarinata*)	*Endothyra*	n. m.
*fultoni*	*Endothyra*	n. m.
*gregorsoni* (syn. of *macromphalus*)		*Endothyra*
*hanleyi*	*Endothyra*	n. m.
*macromphalus*	*Endothyra*	*Endothyra*
*minor*	*Endothyra*	n. m.
*miriensis*		*Endothyra*
*munipurensis* (syn. of *serica*)	*Chersaecia*	n. m.
*oakesi*		*Endothyra*
*oglei*	*Chersaecia*	n. m.
*pinacis*	*Endothyra*	*Endothyra*
*plectostoma*	*Endothyra*	n. m.
*serica*	*Chersaecia*	n. m.
*sowerbyi*	*Endothyra*	n. m.
*tricarinata*	*Endothyra*	n. m.
*williamsoni*		*Chersaecia*

**Table 5.** Main diagnostic characters of *Endothyrella* species.

Species	Diagnostic characters	Similar species (most similar species in bold)
*aborensis*	depressed Z-shaped palatal plicae; two parietal lamellae (?)	
*affinis*	narrow umbilicus; four hair rows; horizontal parietal plica absent	***plectostoma***, *sowerbyi*, *tricarinata*
*angulata* sp. n.	body whorl shouldered; four hair rows	*dolakhaensis* sp. n., *pinacis*, *minor*
*babbagei*	dextral shell; flattened dorsal side; 14 mm	***inexpectata* sp. n.**, *oglei*, *serica*
*bedfordi*	single parietal lamella with long lower plica; posterior ends of palatal plicae with several additional small denticles	
*blanda*	conical dorsal side; 7 hair rows	*macromphalus*, *minor*, *robustistriata* sp. n., ***williamsoni***
*brahma*	three parallel, horizontal parietal plicae anterior to the lamella	
*dolakhaensis* sp. n.	rather conical dorsal side; slightly angulated body whorl; 5 hair rows	*angulata* sp. n., *sowerbyi*
*fultoni*	large size (19.9–20.3 mm); reversed trapezoid shell shape	
*inexpectata* sp. n.	dextral shell; flattened dorsal side; 6.6–6.7 mm	***babbagei***, *oglei*, *serica*
*macromphalus*	nearly flat dorsal side; smooth ventral side	*blanda*, ***minor***, ***robustistriata* sp. n.**, *williamsoni*
*minor*	flat dorsal side; four hair rows	*blanda*, ***macromphalus***, ***robustistriata* sp. n.**, *williamsoni*
*miriensis*	prominent spiral sculpture	
*nepalica* sp. n.	hairless shell; domed dorsal side; rounded body whorl; simple palatal plicae	*oakesi*, *pinacis*
*oakesi*	hairless shell; slightly domed dorsal side; rounded body whorl; complicated palatal plicae	*nepalica* sp. n., *pinacis*
*oglei*	dextral shell; 16.8–16.9 mm; protoconch without groove	*babbagei*, *inexpectata* sp. n., ***serica***
*pinacis*	hairless shell; slightly elevated dorsal side; shouldered body whorl; simple palatal plicae	*nepalica* sp. n., *oakesi*
*plectostoma*	very narrow umbilicus; five hair rows; horizontal parietal plica present	***affinis***, ***sowerbyi***, *tricarinata*
*robustistriata* sp. n.	elevated spire; smooth ventral side; strongly reticulated dorsal surface	*blanda*, ***macromphalus***, ***minor***, *williamsoni*
*serica*	dextral shell; protoconch with groove	*babbagei*, *inexpectata* sp. n., ***oglei***
*sowerbyi*	narrow umbilicus; thin peristome; five hair rows; weak horizontal parietal plica	*affinis*, *dolakhaensis* sp. n., ***plectostoma***, *tricarinata*
*tricarinata*	very narrow umbilicus;, shouldered whorls; four hair rows	***affinis***, ***plectostoma***, ***sowerbyi***
*williamsoni*	conical dorsal side; hairless shell; long horizontal parietal plica	***blanda***, *macromphalus*, *minor*, *robustistriata* sp. n.

##### Distribution.

**Figure 3.**
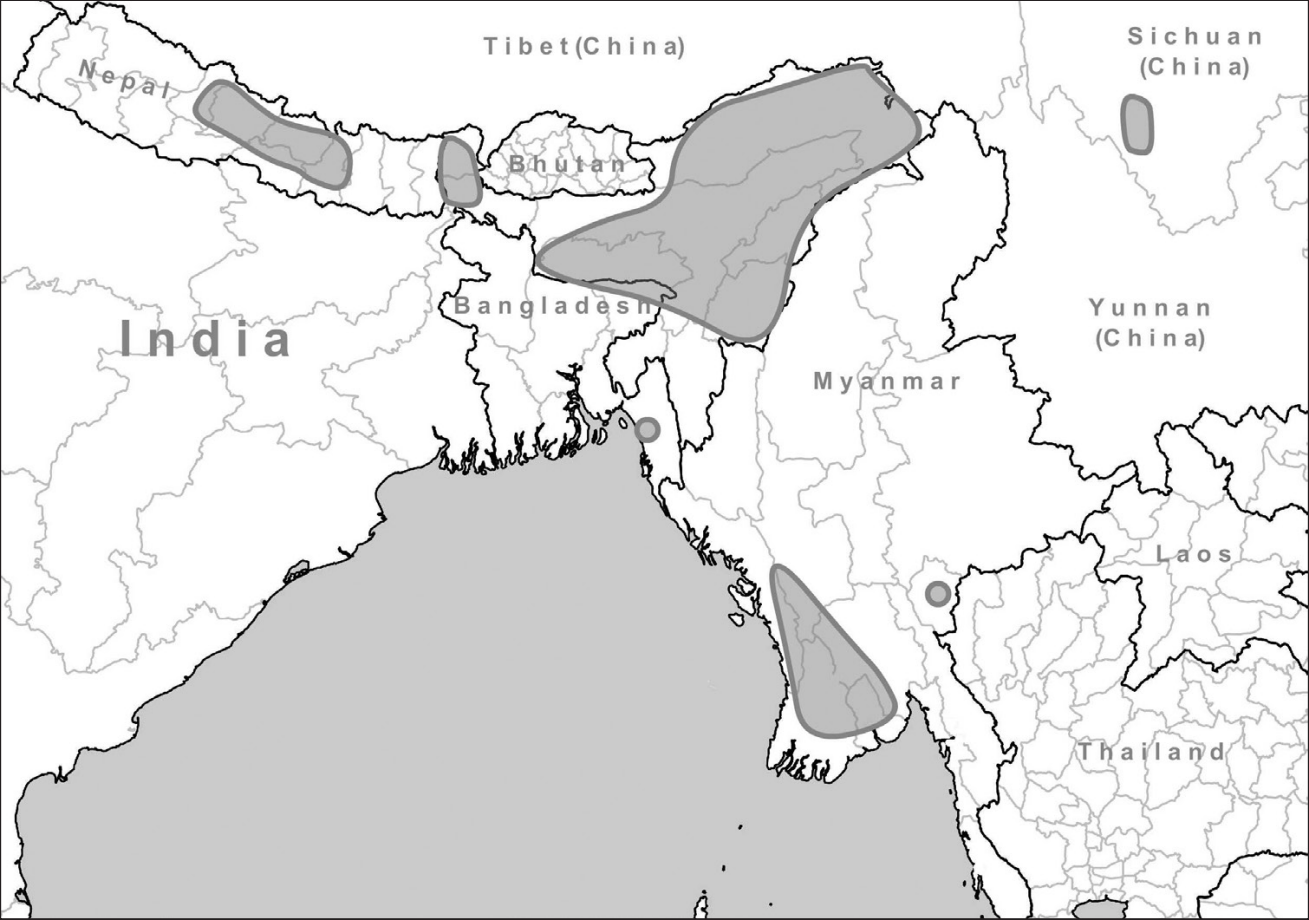
Known distribution of the genus *Endothyrella* Zilch, 1960.

The distribution of this genus is restricted to Nepal, northeastern India and the province Sichuan in China. One species (*Endothyrella plectostoma*) was reported from Myanmar (Figure 3).

### Dextral species

#### Endothyrella babbagei

Taxon classificationAnimaliaPulmonataPlectopylidae

(Gude, 1915)

Figures 4A , 6C

**Figure 4.**
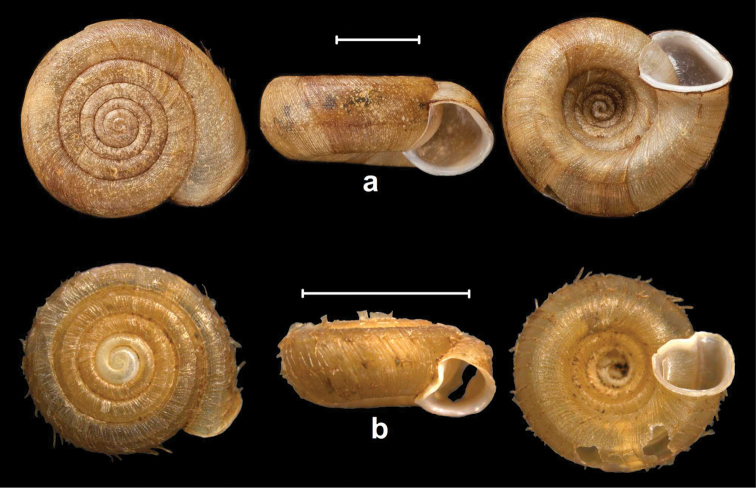
Shells of *Endothyrella* species. **A** *Endothyrella babbagei* (Gude, 1915), NHMUK 1903.7.1.3529 (holotype) **B** *Endothyrella inexpectata* Páll-Gergely, sp. n., NHMUK 20140023 (holotype). Photos: H. Taylor (**A**) and B. Páll-Gergely (**B**). Scale represent 5 mm.

Endothyrella babbagei 1915 Plectopylis (Sinicola) babbagei Gude: Records of the Indian Museum, 8: 512–513, Plate 42, figs 4a–d. [“Luyor Peak, Abor Hills, alt. 7200 ft. Lat. 28°45': Long. 95°45'].
Endothyrella babbagei 1920 Plectopylis (Sinicola) babbagei, — Gude: Proceedings of the Malacological Society of London, 14 (2–3): 64.
Endothyrella babbagei 2013 *Endothyrella babbagei*, — Páll-Gergely & Hunyadi: Archiv für Molluskenkunde, 142 (1): 5.

##### Types.

Peak Luyor, Abor Hills, 7,200 ft, leg. C.F.G. Oakes R.E., NHMUK 1903.7.1.3529. (holotype, Figures 4A, 6C).

##### Diagnosis.

Shell small, dextral, slightly concave above; widely umbilicated; hairs arranged in three spiral lines on the body whorl; callus strong, palatal plicae short, simple, parietal wall with a single curved lamella.

##### Measurements

(in mm): D: 14.4, H: 6.3 (n = 1).

##### Differential diagnosis.

For differences with *Endothyrella oglei*, and *Endothyrella serica* and *Endothyrella inexpectata* sp. n., see there. See also Table 5.

##### Distribution.

Only known from the type locality (Figure 7).

#### Endothyrella inexpectata

Taxon classificationAnimaliaPulmonataPlectopylidae

Páll-Gergely sp. n.

http://zoobank.org/183E6262-0DD8-4881-BBFC-61F608546481

Figures 4B , 6F , 8D , 9A–B

##### Type material.

China, Sichuan Sheng (四川省), Panzhihua Shi (攀枝花市), Yanbian Xian (盐边县), Qinghe Xiang (箐河乡), Qinghepubu (箐河瀑布), Xianrendong (仙人洞), 1410 m, 27°03.834'N, 101°23.611'E, leg. Hosoda, T., Ohara, K., Okubo, K., Otani, J. U., 12.09.2013, NHMUK 20140023 (holotype, Figures 4B, 6F, 8D, 9A–B), JUO/1 (paratype), TH/1 (paratype = juvenile shell); China, Sichuan Sheng (四川省), Liangshan Yizu Zizhizhou (凉山彝族自治州), Yanyuan Xian (盐源县), Bainiao Zhen (白鳥鎮), Kedengrongdong (柯登溶洞) (cave), 2620 m, 27°43.103'N, 101°31.021'E, leg. Hosoda, T., Ohara, K., Okubo, K., Otani, J. U., 13.09.2013, JUO/1 juvenile shell (not paratype); Sichuan Sheng (四川省), Liangshan Zhou (凉山州), Yanyuan Xian (盐源县), Baiwu Zhen (白乌镇), eastern edge of Kedeng Cun (柯登村), 2640 m, 27°43.897'N, 101°31.208'E, leg. Hunyadi, A., Szekeres, M., 11.06.2015., HA/1 paratype.

##### Diagnosis.

Shell very small, dextral, almost flat, relatively widely umbilicated with elevated callus; hairs standing in three lines on the body whorl; parietal wall with a single, curved lamella; palatal wall with six short plicae.

##### Description.

Shell dextral, with almost flat, very slightly domed dorsal side (protoconch slightly elevates from the dorsal surface); brownish or slightly reddish in colour; protoconch consists of 1.5–1.75 whorls, first whorls rather smooth, last 0.25–0.5 whorl regularly ribbed (Figure 6F); teleoconch with irregular, rough growth lines and spiral structure; sculpture stronger on the dorsal surface but still well-visible on the ventral surface; deciduous, slim and flat folds standing in three lines on the body whorl (Figure 8D); whorls 4.75, very much bulging, separated by deep suture; umbilicus moderately wide and deep; apertural lip whitish, thickened and slightly reflexed; callus strong, elevated, sharp and slightly S-shaped; with canals at both ends; no fold in the aperture.

One specimen (the holotype) was opened. The armature is situated very close to the aperture, palatal plicae visible from oblique view through the aperture. Parietal wall with a single curved lamella without additional denticles; arms of the lamella pointing posteriorly; palatal wall with six very short plicae becoming narrower posteriorly; the last one with an additional denticle posteriorly (Figures 9A–B).

##### Measurements

(in mm): D: 6.6–6.7, H: 3.0–3.1 (n = 2, from different localities).

##### Differential diagnosis.

*Endothyrella babbagei* is much larger than *Endothyrella inexpectata* sp. n., and it has flatter whorls and has a weaker callus than the new species. *Sinicola* species of the same size have a keeled or shouldered body whorl and have two parallel parietal plicae anterior to or above the lamella (one near the upper, the other near the lower suture). *Sicradiscus invius* also occurs in Sichuan, but it is smooth (glossy) and has a strong apertural fold. See also under *Endothyrella oglei* and *Endothyrella serica* and Table 5.

##### Etymology.

The name inexpectata (meaning unexpected in Latin) refers to the surprizing new, especially dextral *Endothyrella* species in China.

##### Type locality.

Sichuan Sheng (四川省), Panzhihuashi (攀枝花市), Yanbian Xian (盐边县), Qinghe Xiang (箐河乡), Qinghepubu (箐河瀑布), Xianrendong (仙人洞), 1410 m, 27°03.834'N, 101°23.611'E.

##### Distribution.

*Endothyrella inexpectata* sp. n. is known from two localities in western Sichuan province, China (Figure 7).

#### Endothyrella oglei

Taxon classificationAnimaliaPulmonataPlectopylidae

(Godwin-Austen, 1879)

Figures 5A , 6A

Endothyrella oglei 1879a Helix (Plectopylis) Oglei Godwin-Austen: Journal of the Asiatic Society of Bengal, 48 (2): 3, Plate 1, figs 2, 2a–c. [“Near Sadiya, Assam”].
Endothyrella oglei 1887 Helix (Plectopylis) oglei, — Tryon: Manual of Conchology…, 2 (3): 159, Plate 36, figs 29–31.
Endothyrella oglei 1898 *Plectopylis oglei*, — Gude: Science Gossip, 4: 263, figs 68a–h.
Endothyrella oglei 1899c Plectopylis (Chersaecia) oglei, — Gude: Science Gossip, 6: 148.
Endothyrella oglei 1899d Plectopylis (Chersaecia) oglei, — Gude: Science Gossip, 6: 175, 176.
Endothyrella oglei 1914b Plectopylis (Chersaecia) oglei, — Gude: The Fauna of British India…: 73, 92–93, figs 39a–h.

##### Types.

**Figure 5.**
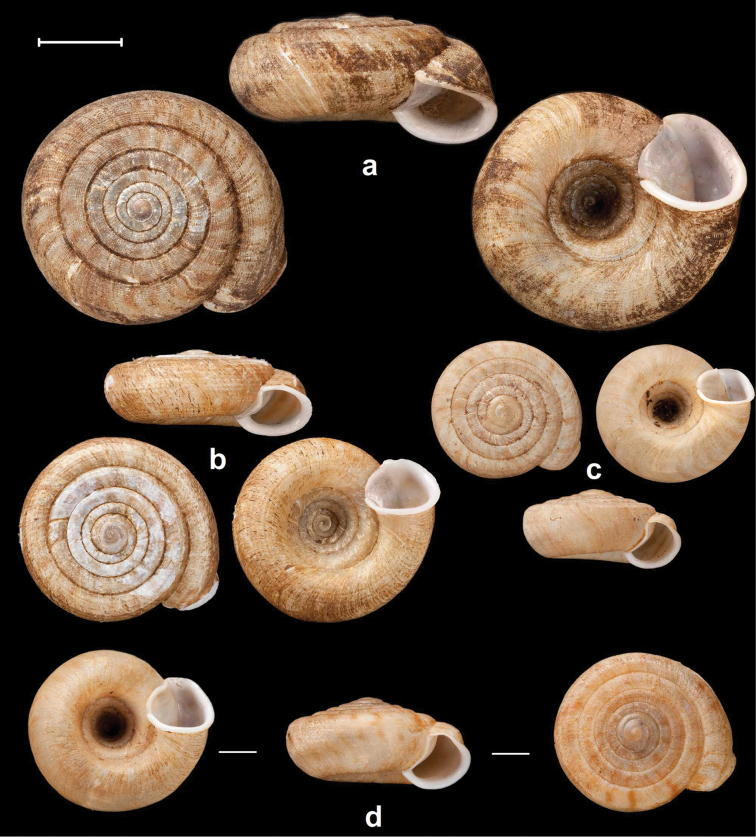
Shells of *Endothyrella* species. **A** *Endothyrella oglei* (Godwin-Austen, 1879), NHMUK 1903.7.1.740. (syntype) **B** *Endothyrella serica* (Godwin-Austen, 1875), NHMUK 1903.7.1.741 (syntype of *serica*) **C** *Endothyrella serica*, NHMUK 1903.7.1.744 (syntype of *serica*) **D** *Endothyrella serica*, NHMUK 1903.7.1.742. (syntype of *munipurensis*). All photos by Harold Taylor (NHMUK). Scale represent 5 mm.

**Figure 6.**
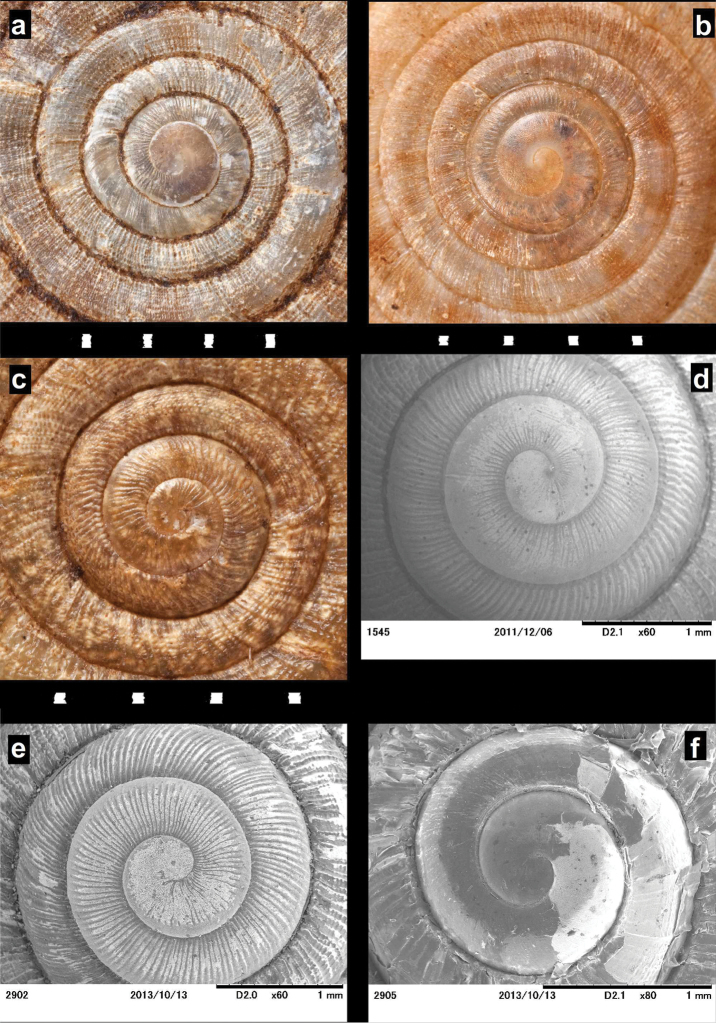
Photos (**A–C**) and SEM images (**D–F**) of *Endothyrella* protoconchs. **A** *Endothyrella oglei* (Godwin-Austen, 1879), same data as in Fig. 5 **B** *Endothyrella serica* (syntype of *munipurensis*, same data as in Fig. 5.) **C** *Endothyrella babbagei* (Gude, 1915), same data as in Fig. 3 **D** *Endothyrella plectostoma* (Benson, 1836), MNHN 2012-27053 **E** *Endothyrella nepalica* Budha & Páll-Gergely sp. n., paratype from the Siddha Cave **F** *Endothyrella inexpectata* Páll-Gergely, sp. n., (holotype). **A–C** Harold Taylor **D–F** B. Páll-Gergely.

Sadia, E. Assam, leg. Ogle, NHMUK 1903.7.1.740. (4 syntypes, Figure 5A, 6A).

**Figure 7.**
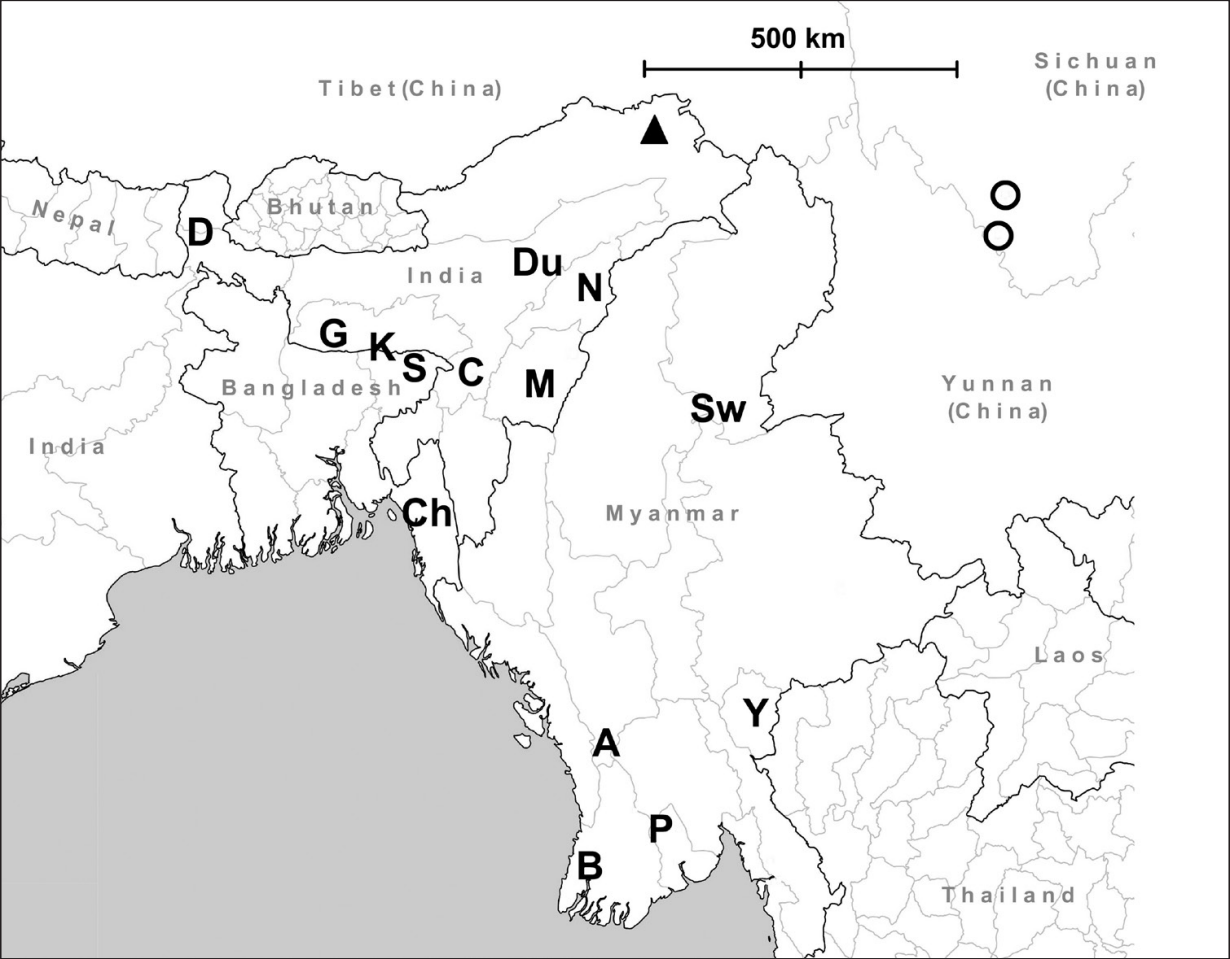
Distribution of *Endothyrella* species. Empty circle: *Endothyrella inexpectata* sp. n.; Filled triangle, top up: type locality of *Endothyrella babbagei*. Letters indicate localities of *Endothyrella plectostoma* (Benson, 1836). Abbreviations: A Arakan Hills
B Bassein (= Pathein)
C Silchar (Cachar)
Ch Chittagong (Ghoramara)
D Darjeeling
Du Dunsiri valley
G Garo Hills
K Khasi Hills
M Manipur
N Naga Hills
P Pegu (= Bago)
S Sylhet
Sw Shwegu
Y Ywathit.

**Figure 8.**
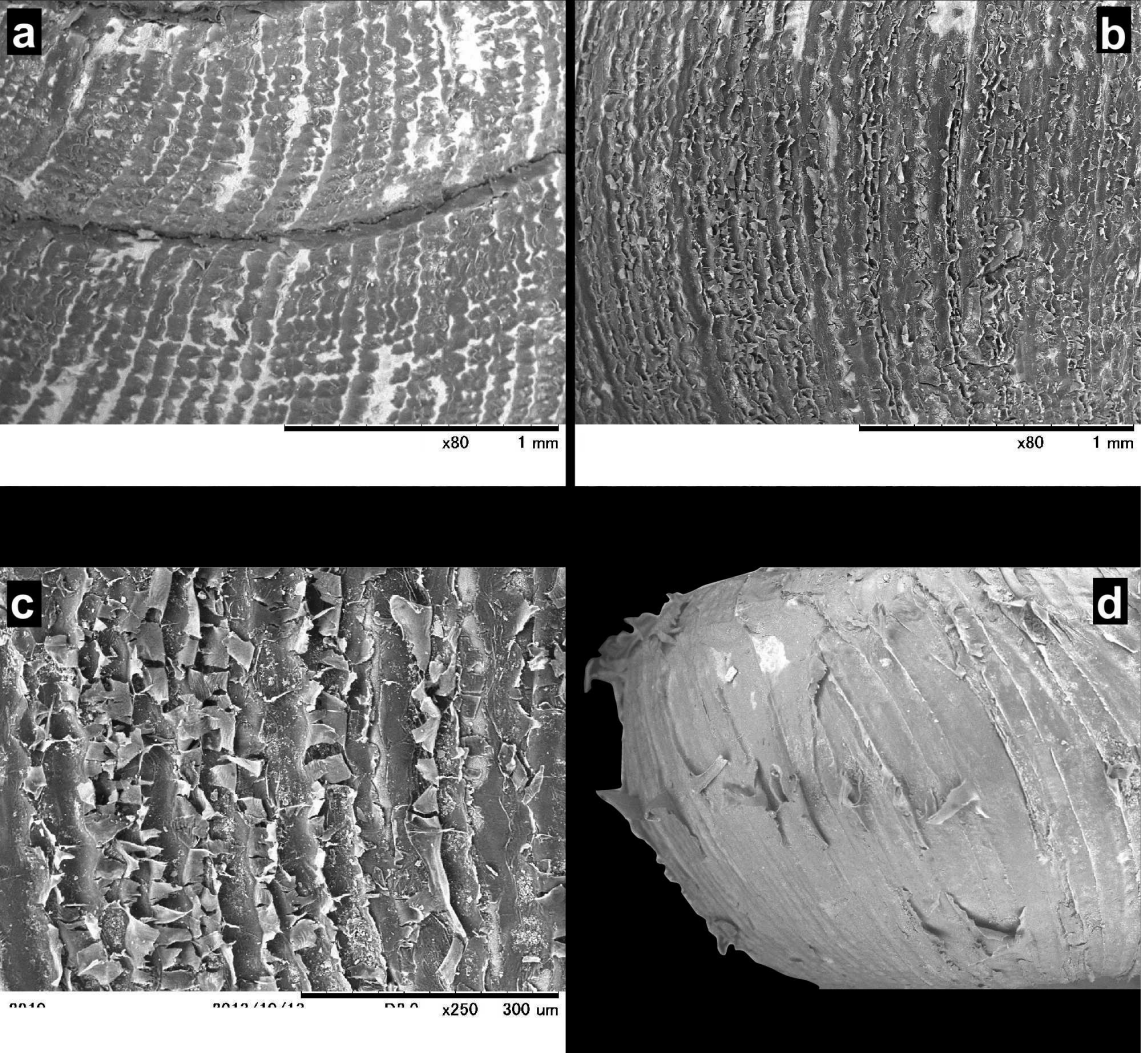
SEM images of *Endothyrella* shells. **A** *Endothyrella nepalica* Budha & Páll-Gergely, sp. n., 4^th^, 5^th^ whorl, for locality see Fig. 6. **B** *Endothyrella nepalica* Budha & Páll-Gergely, sp. n. body whorl **C** *Endothyrella nepalica* Budha & Páll-Gergely, sp. n. body whorl **D** *Endothyrella inexpectata* Páll-Gergely, sp. n., body whorl (holotype). All images by B. Páll-Gergely.

**Figure 9.**
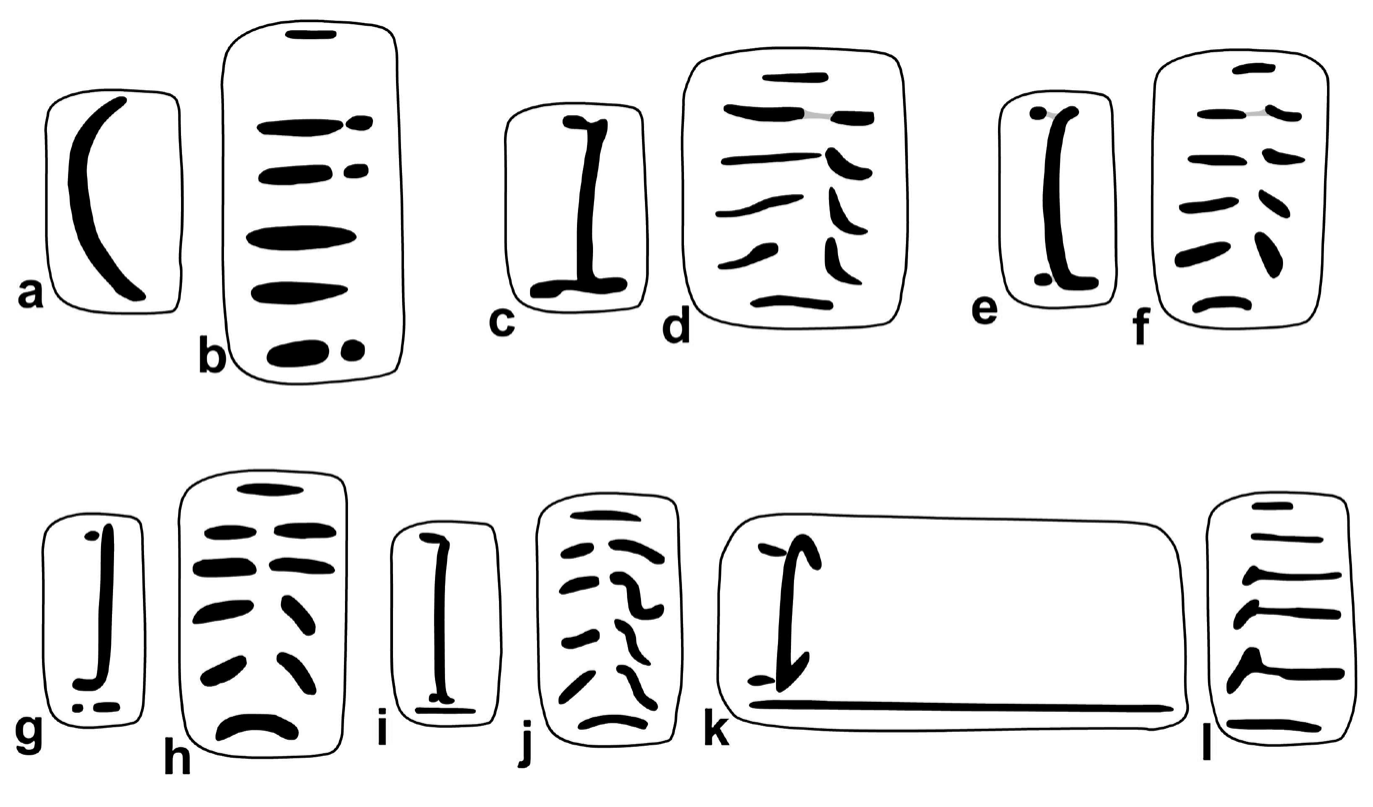
Parietal (**A, C, E, G, I, K**) and palatal (**B, D, F, H, J, L**) lamellation of *Endothyrella* spp. **A–B** *Endothyrella inexpectata* sp. n. (holotype) **C–D** *Endothyrella nepalica* sp. n., for locality, see Fig. 6. **E–F** *Endothyrella nepalica* sp. n., shell from Mahadevsthan **G–H** *Endothyrella dolakhaensis* sp. n., shell from the type locality **I–J** *Endothyrella angulata* sp. n., shell from the type locality **K–L** *Endothyrella robustistriata* sp. n., NHMUK 1903.7.1.767 (two different specimens). Outer view: **B, L**; inner view: **D, F, H, J**. Diagrammatic.

##### Diagnosis.

Shell middle sized, dextral, yellowish-reddish striped with moderately wide umbilicus and somewhat domed dorsal surface; callus strong, palatal plicae divided at their middle and the posterior fragments are connected by a ridge; parietal wall with a single curved lamella with posteriorly elongated upper and lower ends. Probably at least the upper elongation is homologous with the posterior denticle of other *Endothyrella* species.

##### Measurements

(in mm): D: 16.8–16.9, H: 7.7–8.1 (n = 2, type series).

##### Differential diagnosis.

*Endothyrella babbagei* and *Endothyrella inexpectata* sp. n. differ from the *Endothyrella oglei* by the flat dorsal surface of the shell and the presence of hairs arranged in three rows on the body whorl. See also under *Endothyrella serica* and Table 5.

##### Remarks.

The information published by Gude (1914b) (major diameter 27, minor diameter 25 mm) is wrong; it probably refers to “*Chersaecia*” *andersoni*.

##### Distribution.

**Figure 10.**
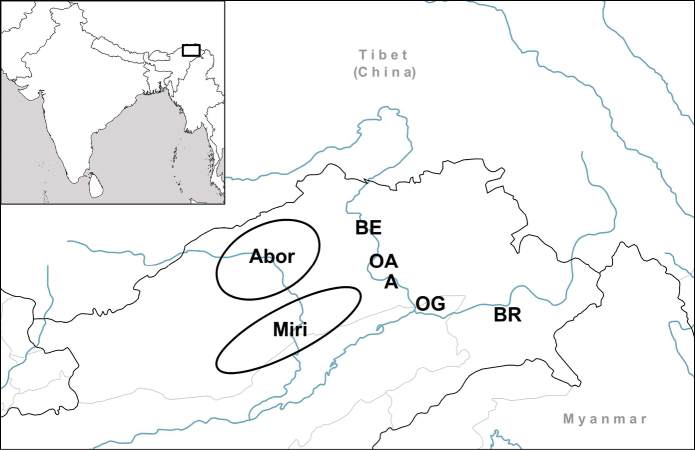
Distribution of *Endothyrella* species in Northeastern India. Abbreviations: A type locality of *Endothyrella aborensis* (Gude, 1915); Abor Abor Hills (type locality of *Endothyrella williamsoni* (Gude, 1915)
BE type locality of *Endothyrella bedfordi* (Gude, 1915)
BR Type locality of *Endothyrella brahma* (Godwin-Austen, 1879); Miri Miri Hills (type locality of *Endothyrella miriensis* (Gude, 1915)
OA Type locality of *Endothyrella oakesi* (Gude, 1915)
OG Type locality of *Endothyrella oglei* (Godwin-Austen, 1879).

The species is known from the type locality only (Figure 10).

#### Endothyrella serica

Taxon classificationAnimaliaPulmonataPlectopylidae

(Godwin-Austen, 1875)

Figures 5B–D , 6B

Endothyrella serica 1875 Helix (Plectopylis) serica Godwin-Austen: Proceedings of the Zoological Society of London: 608, 609, 612, Plate 73, figs 5a–c. [“on the peak of Henozdan, Burrail range” “above 5000 feet on the same range as far east as the Kopamedza ridge”] (1874, part IV, published in 1875; see Duncan 1937).
Endothyrella serica 1875 Helix (Plectopylis) munipurensis Godwin-Austen **new synonym**: Proceedings of the Zoological Society of London: 610, 612, Plate 73, figs 6a–c. [“At the end of the Ihang valley, Munipúr, at about 3000–4000 feet”] (1874, part IV, published in 1875; see Duncan 1937).
Endothyrella serica 1875 *Helix sericata* (sic.), — Hanley & Theobald: Conchologia Indica...: 53, Plate 132, figs 8, 9.
Endothyrella serica 1878 Helix (Plectopylis) serica, — Nevill: Hand list of Mollusca in the Indian Museum, Calcutta...: 71. [“Hengdan Peak and Burrail”].
Endothyrella serica 1879a Helix (Plectopylis) serica, — Godwin-Austen: Journal of the Asiatic Society of Bengal, 48 (2): 3.
Endothyrella serica 1887 Helix (Plectopylis) serica, — Tryon: Manual of Conchology…, 2 (3): 159, Plate 34, figs 49–52.
Endothyrella serica 1887 Helix (Plectopylis) Munipurensis, — Tryon: Manual of Conchology…, 2 (3): 160, Plate 34, figs 56–58.
Endothyrella serica 1897a *Plectopylis serica*, — Gude: Science Gossip, 3: 205–206, figs 31a–c.
Endothyrella serica 1897 *Plectopylis serica*, — Gude: Science Gossip, 3: 246.
Endothyrella serica 1898 *Plectopylis munipurensis*, — Gude: Science Gossip, 4: 263–264, figs 69a–g.
Endothyrella serica 1899c Plectopylis (Chersaecia) serica, — Gude: Science Gossip, 6: 148.
Endothyrella serica 1899c Plectopylis (Chersaecia) munipurensis, — Gude: Science Gossip, 6: 148.
Endothyrella serica 1899d Plectopylis (Chersaecia) serica, — Gude: Science Gossip, 6: 175, 177.
Endothyrella serica 1899d Plectopylis (Chersaecia) munipurensis, — Gude: Science Gossip, 6: 175, 176.
Endothyrella serica 1914b Plectopylis (Chersaecia) serica, — Gude: The Fauna of British India…: 73, 93–94, figs 40a–c.
Endothyrella serica 1914b Plectopylis (Chersaecia) munipurensis, — Gude: The Fauna of British India…: 73, 94–95, figs 41a–g.

##### Types.

Khunho, H.S. Naga Hills, leg. Godwin-Austen, NHMUK 1903.7.1.741 (8 syntypes of *serica*, Figure 5B); Hengdan P., Naga Hills, leg. Godwin-Austen, NHMUK 1903.7.1.744 (6 syntypes of *serica*, Figure 5C); Munipur Hills, head of the Ihang valley, Munipur, leg. Godwin-Austen, NHMUK 1903.7.1.742. (6 syntypes of *munipurensis*, Figure 5D).

##### Additional material examined.

Naga Hills, coll. Godwin-Austen, NHMUK 1903.7.1.743/4 (under the name *munipurensis*); Japvo Peak, Nr. Kohima, Naga Hills, NHMUK 20150128/8; Lhota Naga, coll. Godwin-Austen, NHMUK 1903.7.1.745/6; no locality, leg. Maxwell, coll. Godwin-Austen, NHMUK 20150129/5; India, Hengdan Peak, NHMUK 1891.3.17.356–357/2; India, NHMUK 1874.4.26.2/2; Khasi Hills, coll. W. Blanford, NHMUK 1906.2.2.360/2.

##### Diagnosis.

Shell very small to small, dextral, yellowish-reddish striped with moderately wide umbilicus and depressed conical dorsal surface; callus strong, palatal plicae more or less straight, simple or have dichotomously divided posterior ends; parietal wall with a single curved lamella with denticles near the upper and lower ends posteriorly, which occasionally fuse with the lamella.

##### Measurements

(in mm): D: 9.7–9.9, H: 4.4–4.8 (n = 3, NHMUK 1903.7.1.744); D: 9.9–13, H: 4.9–5.5 (n = 4, NHMUK 1903.7.1.741); D: 10.9–11.7, H: 5.1–5.7 (n = 3, NHMUK 1903.7.1.742).

##### Differential diagnosis.

*Endothyrella babbagei* and *Endothyrella inexpectata* sp. n. differ from *Endothyrella serica* by the flat dorsal surface of the shell and the presence of three rows of hairs on the body whorl. *Endothyrella oglei* differs from the also dextral *Endothyrella serica* by the much larger size, the absence of the groove on the protoconch, which runs parallel with the suture in *Endothyrella serica*, and the morphology of the lamella which has only posteriorly elongated ends. See also Table 5.

##### Distribution.

**Figure 11.**
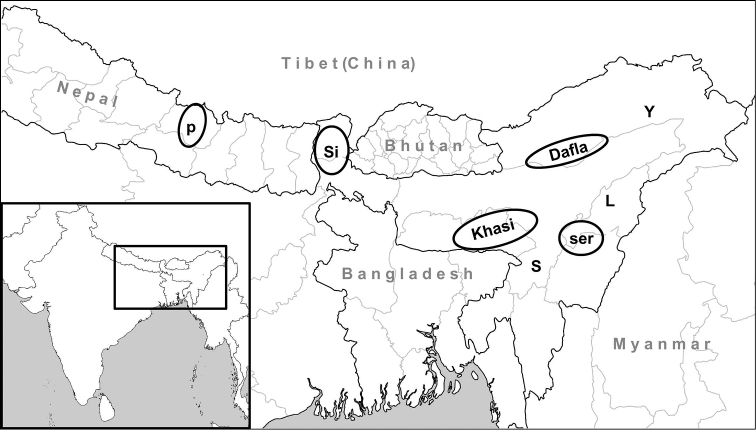
Distribution of *Endothyrella* species in Northeastern India. Abbreviations: Dafla Dafla Hills (locality of *Endothyrella macromphalus*); Khasi Khasi Hills (locality of *Endothyrella affinis*, *Endothyrella fultoni*, *Endothyrella robustistriata* sp. n., *Endothyrella macromphalus*, *Endothyrella minor* and *Endothyrella tricarinata*), L Lhota Naga (locality of *Endothyrella robustistriata* sp. n.)
P Nepalese localities of *Endothyrella minor*
SER
*Endothyrella serica* (Godwin-Austen, 1875)
S Silchar (locality of *Endothyrella blanda*)
**SI** Sikhim (locality of *Endothyrella blanda*, *Endothyrella minor*, *Endothyrella pinacis*) **Y** Yamne valley (type locality of *Plectopylis gregorsoni*).

The species is recorded from the Naga Hills (see also remarks). “*Plectopylis munipurensis*” was described from “end of the Ihang valley” (Figure 11).

##### Remarks.

Godwin-Austen (1875) described Helix (Plectopylis) serica and Helix (Plectopylis) munipurensis in the same publication. He did not mention the differences between the two species. According to the illustrations and the identification key in the original description, the upper end of the lamella in *munipurensis* is more elongated anteriorly than that of *Helix serica*. Two shells of *Endothyrella serica* were opened from the Hengdan sample, and both had an anteriorly elongated plica. In this respect, and also in shell shape, these shells were more similar to *Endothyrella munipurensis* specimens. In the Khunho sample four shells were opened, three having no or very slight upper elongation, but one had an as long plica as in typical *munipurensis* shells. Examining the type specimens of the two species we have not found significant differences. The width of the umbilicus and the height of the spire showed some variability. Therefore we synonymize *munipurensis* with *serica*. We choose Helix (Plectopylis) serica to be the valid specific name.

In the original description Godwin-Austen (1875) reported the species from the “peak of Henozdan” and from the “Kopamedza ridge”. The second sample is probably identical with the one from Khunho in the type collection of the NHM.

Gude (1897h) mentions that according to Godwin-Austen, the correct names for “Henozdan” and “Kopameda” in Gude (1897a) are “Hengdan” and “Kopamedza”, respectively. According to the same erratum, Godwin-Austen also mentioned that the locality of Ponsonby’s shell (Sylhet) is probably incorrect, because *Plectopylis serica* is a very local species, inhabiting altitudes higher than 5000 feet.

### Sinistral species

#### Endothyrella aborensis

Taxon classificationAnimaliaPulmonataPlectopylidae

(Gude, 1915)

Figure 12

Endothyrella aborensis 1915 Plectopylis (Endoplon) aborensis Gude: Records of the Indian Museum, 8: 511–512, Plate 42, Fig. 3a–d. [“Between Renging and Rotung, 2200 ft., Abor country.”].
Endothyrella aborensis 2013 *Endothyrella aborensis*, — Páll-Gergely & Hunyadi: Archiv für Molluskenkunde, 142 (1): 5.

##### Types.

According to the original description, two shells, an adult and a juvenile were collected and finally deposited in the Indian Museum (inventory numbers: 5998 and 6135). Specimen reference collections in the Indian Museum were transferred to the ZSI following foundation of the ZSI in 1916. The ZSI supplied us with two photos of an adult shell under the name of *Plectopylis aborensis*, which they considered as one of the type specimens. These photos, however, clearly showed a different specimen than the one figured in Gude (1915). No other information could be obtained from the ZSI.

##### Diagnosis.

Shell small, sinistral, almost flat, widely umbilicated; callus strong; palatal plicae Z or L-shaped; there are two parietal lamellae, a short upper plica which is in contact with the posterior lamella, and a long lower plica which reaches the peristome.

##### Measurements

(in mm): D: 14, H: 6.5 (according to the original description).

##### Differential diagnosis.

The species was not examined by us, but according to the original description the species differs from all congeners by the short and uniquely shaped palatal plicae, which are depressed Z-shaped, or the lower branch of the “Z” is elongated. See also Table 5.

##### Distribution.

Known from the type locality only (approximately 28°10'N, 95°13'E) (Figure 10).

##### Remarks.

So far, this is the only *Endothyrella* species with two well-developed lamellae. The parietal lamellae show a very unusual arrangement which has not been observed in any other species of Plectopylidae. The two parietal plicae can be the result of teratological duplication which has been reported for some species (Gude 1908b: 347).

**Figure 12.**
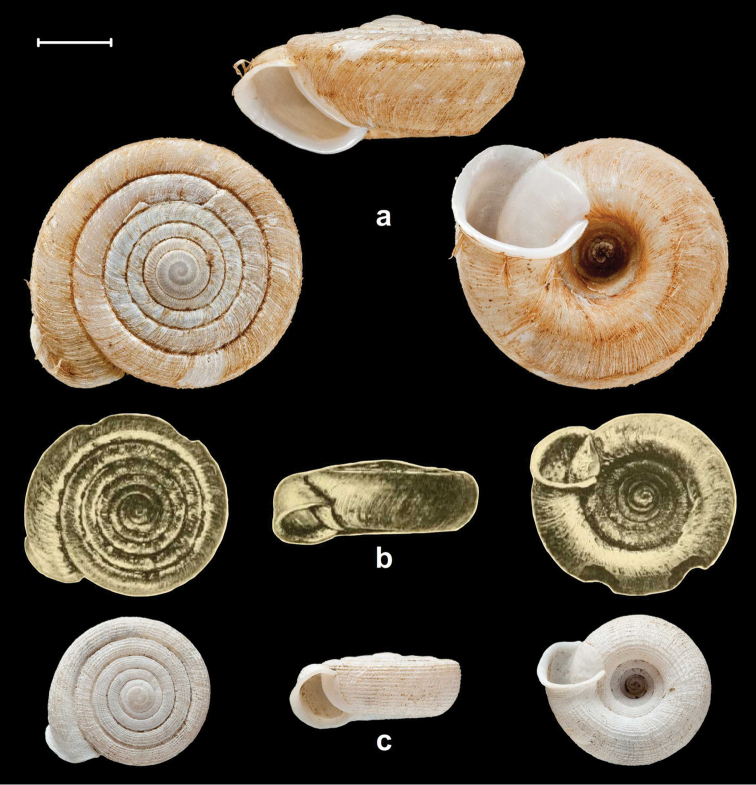
Shells of *Endothyrella* and *Chersaecia* species. **A** *Endothyrella fultoni* (Godwin-Austen, 1892), NHMUK 1903.7.1.301. (syntype) **B** *Endothyrella aborensis* (Gude, 1915), (syntype, photos published in Gude 1915) **C** *Endothyrella miriensis* (Gude, 1915), NHMUK 1903.7.1.3205. (syntype). Photos **A** and **C** by H. Taylor. Scale represent 5 mm.

#### Endothyrella affinis

Taxon classificationAnimaliaPulmonataPlectopylidae

(Gude, 1897)

Figure 13G

Endothyrella affinis 1897b *Plectopylis affinis* Gude: Science Gossip, 3: 276, figs 41a–d. [“Khasia Hills, Assam”].
Endothyrella affinis 1897g *Plectopylis affinis*, — Gude: The Journal of Malacology, 6: 46–48, fig. 3.
Endothyrella affinis 1899c Plectopylis (Endothyra) affinis, — Gude: Science Gossip, 6: 148.
Endothyrella affinis 1899d Plectopylis (Endothyra) affinis, — Gude: Science Gossip, 6: 175, 176.
Endothyrella affinis 1914b Plectopylis (Endothyra) affinis, — Gude: The Fauna of British India…: 73, 84–85, figs 34a–d.

##### Types.

**Figure 13.**
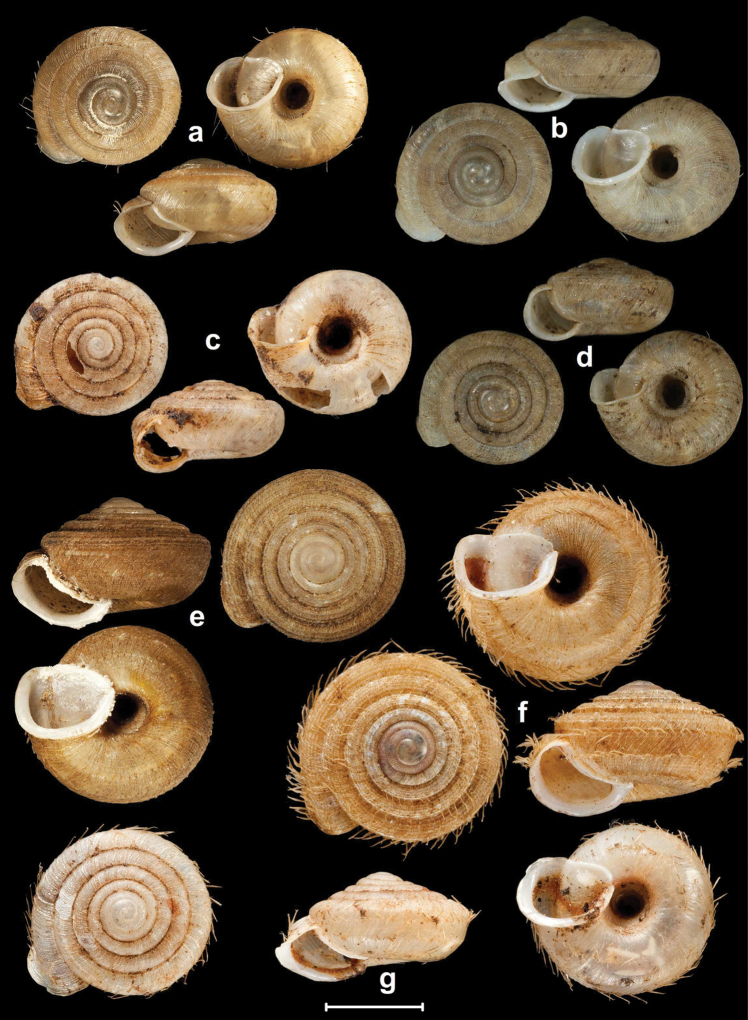
Shells of *Endothyrella* species. **A** *Endothyrella plectostoma* (Benson, 1836), UMZC 102155 (syntype, specimen figured by Gude 1897b) **B** *Endothyrella plectostoma*, SMF 118091 **C** *Endothyrella sowerbyi* (Gude, 1899), NHMUK 1922.8.29.48. (holotype) **D** *Endothyrella sowerbyi*, SMF 346408 **E** *Endothyrella tricarinata* (Gude, 1897), UMZC 102170 (syntype of *tricarinata*) **F** *Endothyrella tricarinata*, NHMUK 1922.8.29.50. (syntype of *exerta*) **G** *Endothyrella affinis* (Gude, 1897), NHMUK 1922.8.29.26 (syntype). Photos: J. Gundry (**A, E**), B. Páll-Gergely (**B, D**) and H. Taylor (**C, F, G**). Scale represent 5 mm.

India, Khasia Hills, ex Fulton, NHMUK 1922.8.29.36 (syntype, Figure 13G); Khasia Hills, NHMUK 1901.4.25.41–43 (3 syntypes).

##### Additional material examined.

India, Khasi Hills, NHMUK 1892.9.22.1–4 (4 specimens); India, NHMUK 1916.3.15.1–2/2 (“showing immature armature”); Khasi Hills, Assam, coll. Salisbury ex coll. Beddome, NHMUK 20150130/3; Khasi Hills, NHMUK 20150131/3; Cherra, leg. Godwin-Austen, NHMUK 20150132/1 juvenile shell; N-Vorderindien, Khasi-Berge, coll. C. R. Boettger 1911, SMF 118096/1 (labelled as “cotype”); Cherrapoonjee, coll. Jetschin ex coll. Gude 1900, SMF 118095/2; India, Khasi Hills, NHMW 34233/2; Khasi Hills, coll. Möllendorff, SMF 150107/3; Khasi-Berge, coll. Möllendorff, ex coll. Gude, SMF 9279/4; Khasi Hills, coll. Bosch ex coll. Rolle, SMF 172074/2; N. O(?) Indien, coll. Steenberg, ZMUC-GAS-1811/1; no locality, coll. Jousseaume, MNHN 2012-27051/2; no locality, coll. Jousseaume, MNHN 2012-27048/29 (strongly shouldered, relatively small shells together with typical ones).

##### Diagnosis.

Shell small, sinistral, yellowish, with narrow umbilicus, conical dorsal surface and shouldered body whorl; hairs are arranged in four rows on the body whorl; callus strong, middle palatal plicae usually divided in the middle; the posterior fragments are oblique, the anterior ones are rather straight; parietal wall with a single, slightly curved lamella with short denticles posteriorly, one above and one below, and a horizontal lower plica which may be divided in the middle.

##### Measurements

(in mm): D: 9.7–10.9, H: 5.4–5.7 (n = 4, SMF 9279); D: 8.5–10.6, H: 5.1–5.6 (n = 3, MNHN 2012-27048).

##### Differential diagnosis.

See under *Endothyrella plectostoma*, *Endothyrella sowerbyi* and *Endothyrella tricarinata* and Table 5.

##### Distribution.

The species is recorded from the Khasi Hills only (Figure 11).

#### Endothyrella angulata

Taxon classificationAnimaliaPulmonataPlectopylidae

Budha & Páll-Gergely sp. n.

http://zoobank.org/0359FD0B-BACA-4B47-A483-1DF6AD3F79A5

Figures 9I–J , 14B

##### Type material.

**Figure 14.**
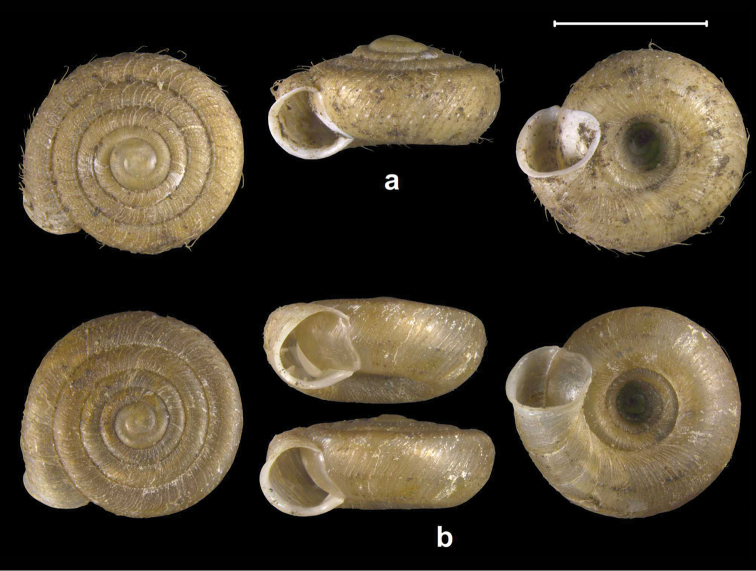
Shells of *Endothyrella* species. **A** *Endothyrella dolakhaensis* Budha & Páll-Gergely, sp. n., CDZMTU001 (holotype) **B** *Endothyrella angulata* Budha & Páll-Gergely, sp. n., CDZMTU018 (holotype). Both photos by E. Bochud. Scale represent 5 mm.

Nepal, Taubas, Bhainse, Makwanpur District, 27°492521'N, 85°04839'E., leg. Budha, P., 30.03.2012., holotype (CDZMTU018, Figure 14B); 3 paratypes and 2 juvenile shells (not paratypes) (CDZMTU019).

##### Diagnosis.

Shell small, sinisttral, with flat dorsal surface and shouldered (keeled) body whorl; hairs are arranged in four rows; parietal lamella simple with a short free horizontal plica below it, and two denticles posterior to the lamella which are in contact with the lamella; middle palatal plicae divided.

##### Description.

Shell sinistral, semi-transparent; protoconch elevated from the flat dorsal surface; colour brownish or greyish; protoconch conspicuously large, consists of 2.5, 2.75 whorls, very finely, regularly ribbed; teleoconch with clearly visible reticulated sculpture dominated by radial growth lines; sculpture somewhat weaker on the ventral surface; very slender, long periostracal folds (hairs) standing in four spiral lines along the body whorl; two closely adjacent rows running with the keel above, one row on the ventral side around the umbilicus, and one row approximately in the middle line of the body whorl; whorls 6.25 (holotype) moderately bulging, separated by relatively deep suture; umbilicus wide and deep; peristome thin, slightly reflexed; callus moderate; no fold in the aperture.

One specimen was opened. Palatal wall with a single, straight lamella, with two short denticles on the posterior side of the lamella, both are in contact with the lamella; a short, free horizontal plica is visible under the lamella; palatal wall with six plicae, first straight, last slightly curved, the middle plicae are divided in the middle, the fragments are horizontal, oblique or Z-shaped (Figure 9I–J).

##### Measurements

(in mm): D: 8.5, H: 3.5 (holotype); D: 5.5, H: 2.5, Wh: 5 (paratype; subadult specimen).

##### Differential diagnosis.

See under *Endothyrella dolakhaensis* sp. n., *Endothyrella minor*, *Endothyrella nepalica* sp. n., *Endothyrella pinacis* and Table 5.

##### Etymology.

The Latin angulatus (cornered, angular) refers to the shouldered/angulated body whorl of the new species.

##### Type locality.

Nepal, Taubas, Bhainse, Makwanpur District, 27°492521'N, 85°04839'E.

##### Distribution.

**Figure 15.**
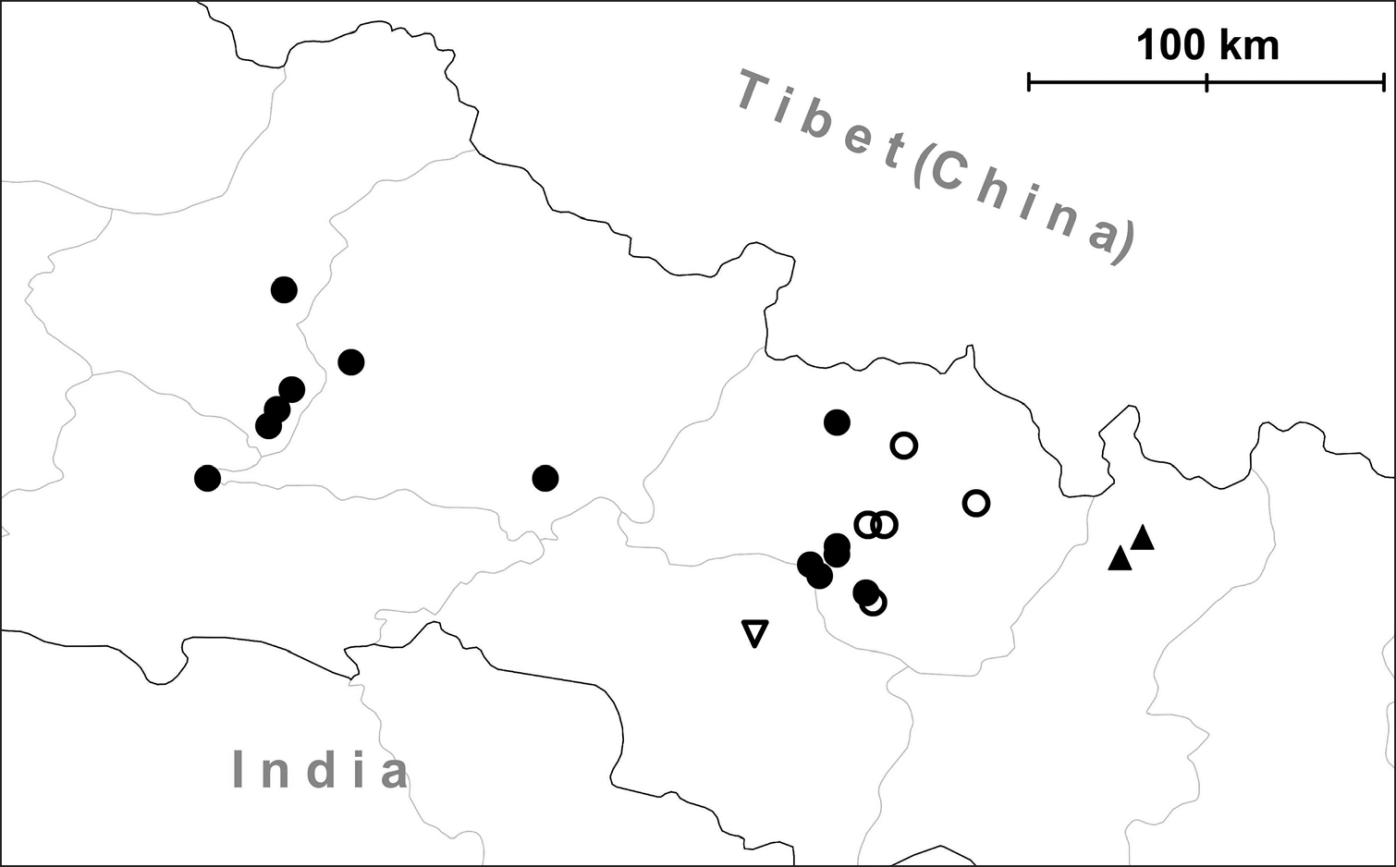
Distribution of *Endothyrella* species in Nepal. Filled circle: *Endothyrella nepalica* sp. n.; filled tringle, top up: *Endothyrella dolakhaensis* sp. n.; empty triangle, top down: *Endothyrella angulata* sp. n.; empty circle: *Endothyrella minor*.

*Endothyrella angulata* sp. n. is known only from the type locality (Figure 15).

#### Endothyrella bedfordi

Taxon classificationAnimaliaPulmonataPlectopylidae

(Gude, 1915)

Figure 16C

Endothyrella bedfordi 1915 Plectopylis (Chersaecia) bedfordi Gude: Records of the Indian Museum, 8: 510–511, plate 42, fig. 2a–d. [“Abor country, Tsanspu Valley, on the Dihang, about 50 miles above the junction of the Sigon River, alt. 2800 ft.”].

##### Types.

**Figure 16.**
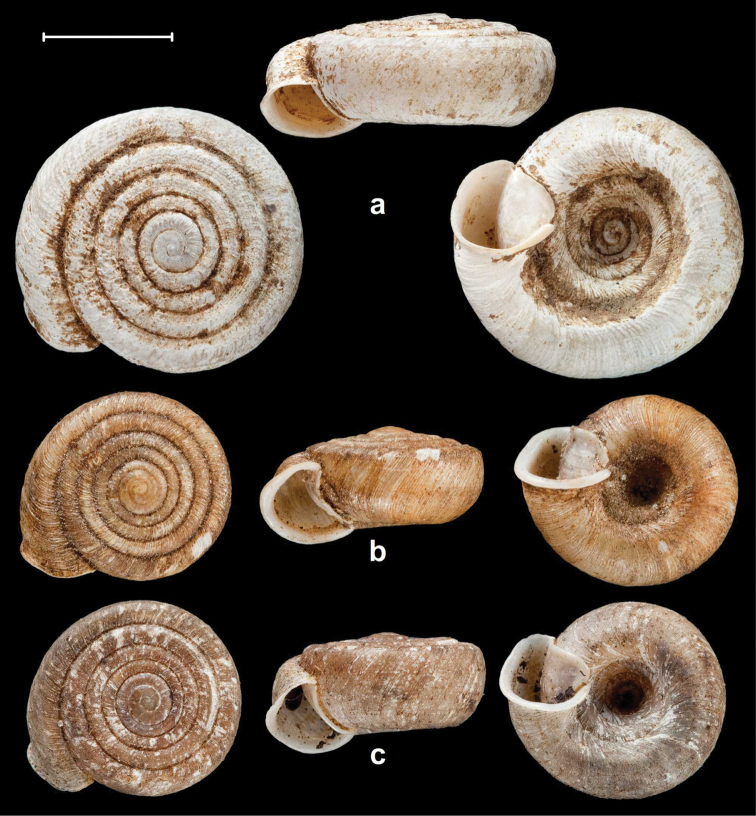
Shells of *Endothyrella* species. **A** *Endothyrella oakesi* (Gude, 1915), NHMUK 1903.7.1.3125. (syntype) **B** *Endothyrella brahma* (Godwin-Austen, 1879), NHMUK 1903.7.1.751. (syntype) **C** *Endothyrella bedfordi* (Gude, 1915), NHMUK 1903.7.1.3584. (syntype). All photos by H. Taylor. Scale represent 5 mm.

Tsanspu Valley Abor Hills, 2800 ft, leg. C.F.G. Oakes R.E., NHMUK 1903.7.1.3584. (2 syntypes, Figure 16C).

##### Diagnosis.

Shell very small, sinistral, brownish, with moderately wide umbilicus, almost flat dorsal surface (only the apex is elevated slightly), and rounded body whorl; callus strong, palatal plicae long, more or less straight horizontal, with dichotomously divided posterior ends and many small denticles at their posterior ends; lamella single, curved, in contact with a lower plica, which runs until the peristome.

##### Measurements

(in mm): D: 9.1, H: 4.9 (n = 1, type series).

##### Differential diagnosis.

*Endothyrella bedfordi* has a single curved parietal lamella with a long lower plica (which reaches the peristome) attached to it, and at the posterior ends of palatal plicae there are several small denticles. These features distinguish *Endothyrella bedfordi* from all congeners. See also Table 5.

##### Distribution.

The species is known from the type locality only (approximately 28°44'N, 94°56'E) (Figure 10).

#### Endothyrella blanda

Taxon classificationAnimaliaPulmonataPlectopylidae

(Gude, 1898)

**Figure 17.**
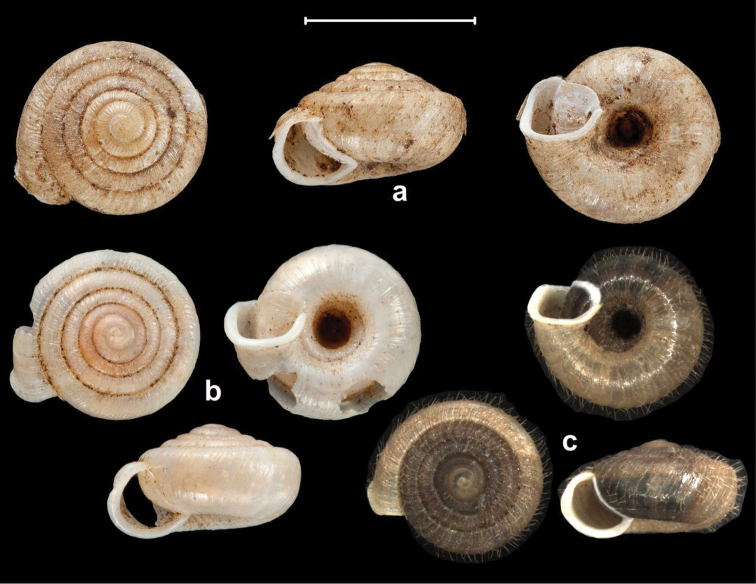
Shells of *Endothyrella* species. **A** *Endothyrella williamsoni* (Gude, 1915), NHMUK 1903.7.1.3087. (syntype) **B** *Endothyrella blanda* (Gude, 1898), NHMUK 1922.8.29.41. (holotype) **C** *Endothyrella blanda*, Silchar Cachar, F. Ede, coll. Godwin-Austen, NHMUK 1903.7.1.502. Photos: B. Páll-Gergely (**C**) and H. Taylor (**A, B**). Scale represent 5 mm.

**Figure 18.**
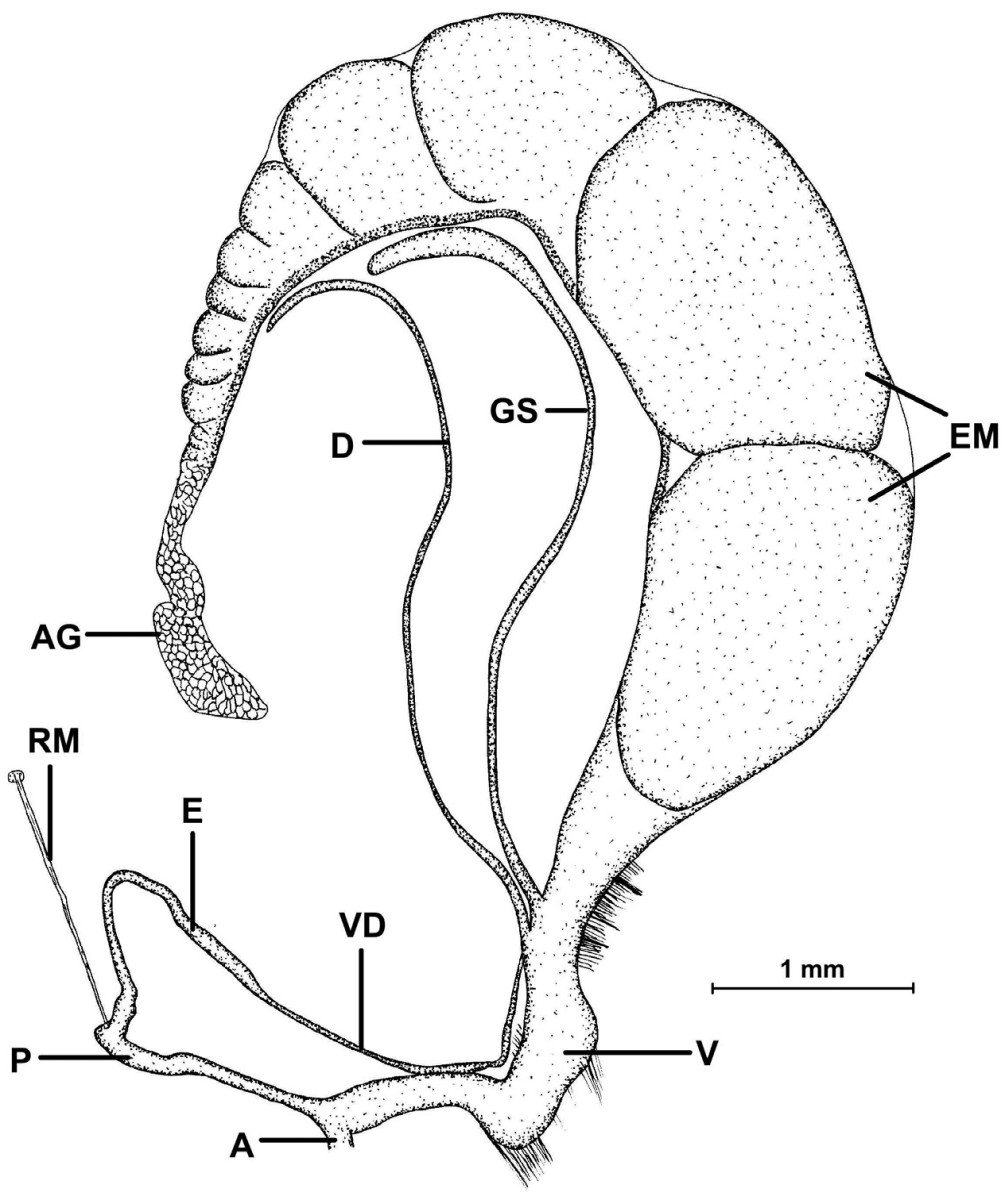
Genital anatomy of *Endothyrella blanda* (Gude, 1898). For locality see Fig. 17C. Abbreviations: A atrium
AG albumen gland
D diverticulum
E epiphallus
EM embryos
GS gametolytic sac
P penis
RM retractor muscle
V vagina
VD vas deferens.

**Figure 19.**
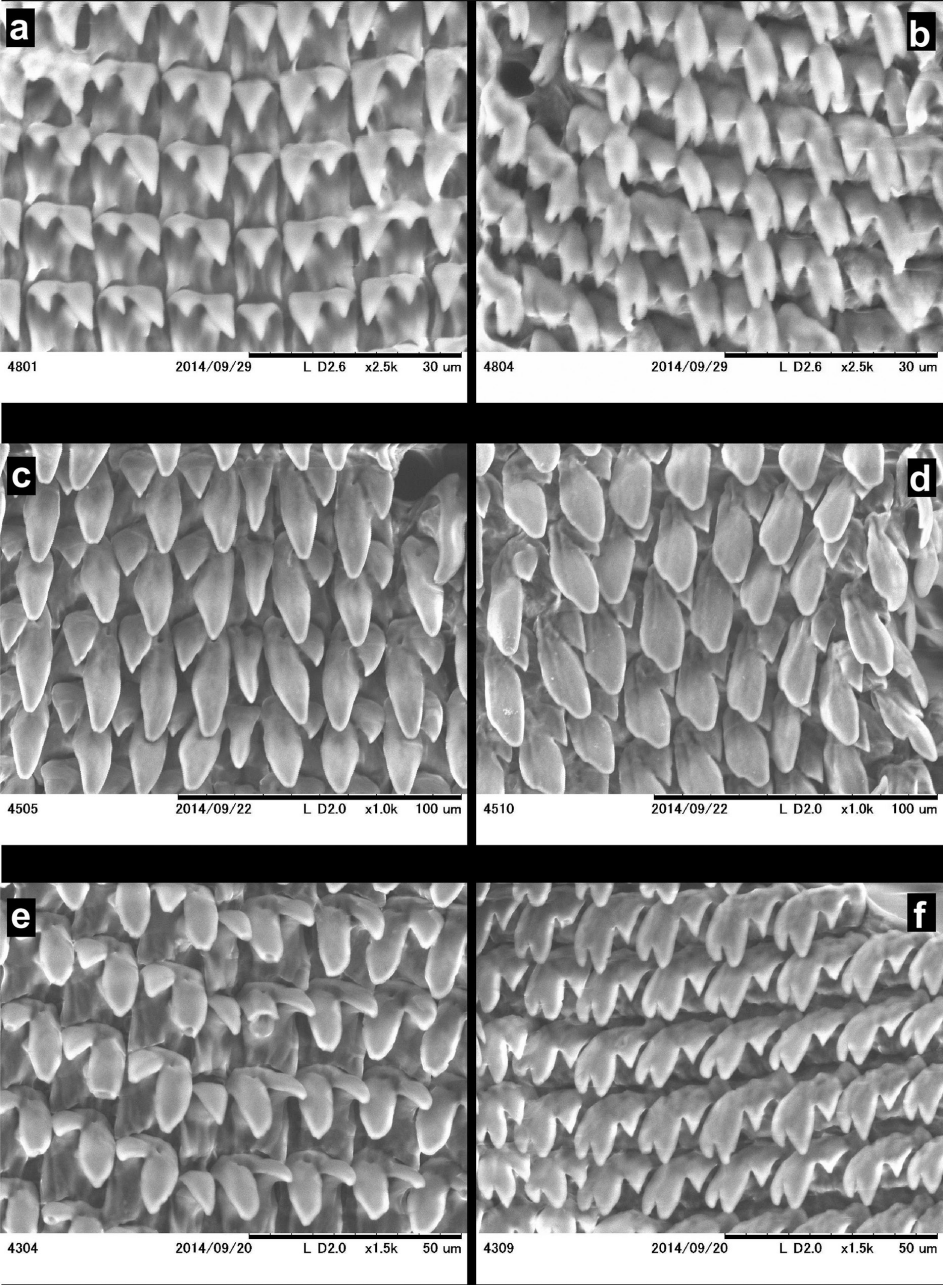
SEM images of the radula of *Endothyrella* species. **A, C, E** central and adjacent lateral teeth **B, D, F** marginal teeth **A–B** *Endothyrella blanda* (Gude, 1898) (For locality see Fig. 17C) **C–D** *Endothyrella fultoni* (Godwin-Austen, 1892) (for locality see Fig. 18) **E–F** *Endothyrella plectostoma* (Benson, 1836), Sikhim, leg. Godwin-Austen, NHMUK 1903.7.1.451. All images by B. Páll-Gergely.

**Figure 20.**
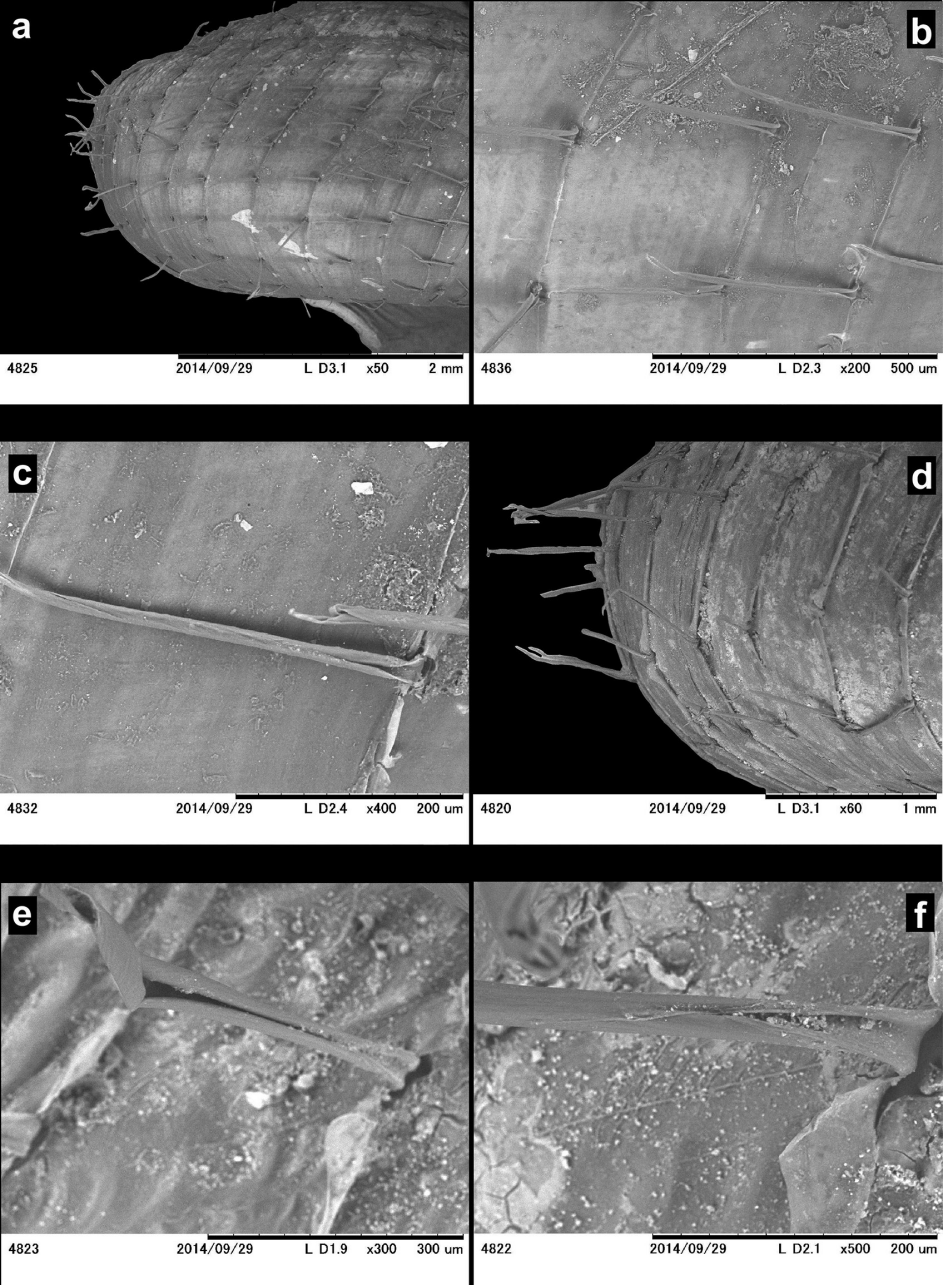
SEM images of *Endothyrella* shells. **A–C** *Endothyrella blanda* (Gude, 1898), For locality see Fig. 17C **D–F** *Endothyrella plectostoma* (Benson, 1836), Sikhim, leg. Godwin-Austen, NHMUK 1903.7.1.451. All images: B. Páll-Gergely.

Figures 17B–C , 18 , 19A–B , 20A–C

Endothyrella blanda 1898 *Plectopylis blanda* Gude: Science Gossip, 4: 264, figs 70 a–f. [“Naga Hills, Assam”]
Endothyrella blanda 1899c Plectopylis (Endothyra) blanda, — Gude: Science Gossip, 6: 148.
Endothyrella blanda 1899d Plectopylis (Endothyra) blanda, — Gude: Science Gossip, 6: 175, 176.
Endothyrella blanda 1900 *Plectopylis blanda*, — Gude: The Journal of Malacology, 7: 34–35, figs 11a–f.
Endothyrella blanda 1914b Plectopylis (Endothyra) blanda, — Gude: The Fauna of British India…: 73, 77–78, figs 28a–f.

##### Types.

Naga Hills, NHMUK 1922.8.29.41., coll. Godwin-Austen (holotype, Figure 17B).

##### Additional material examined.

Richila Peak, Sikkim, India, coll. Ottó, L., MMGY 66425/2; Darjeeling, India, West Bengal, Darjeeling, North Point 900–1400 m asl., under stones in forest clearings, coll. Topál, 1967. HNHM 98849/2; Damsang, coll. Godwin-Austen, NHMUK 20150133/26; Rissetchu, Sikkim, coll. Godwin-Austen, NHMUK 20150135/8; Rissetchu & Richila Peak, W. Bhutan, coll. Godwin-Austen, NHMUK 20150136/33 (several of these are juvenile shells); Sikhim, Nampok, coll. Godwin-Austen, NHMUK 20150137/28; Richila Peak, Sikkim, coll. Godwin-Austen, NHMUK 20150138/102; Risset-Chu, Sikkim, NHMUK 20150139/309; Sikhim, NHMUK 20150140/8 (there is a label with the number “749”); Sikhim, coll. Beddome ex coll. Godwin-Austen, NHMUK 1912.4.16.318/1 (large variety); Sikhim, Rinkpo valley, NHMUK 1906.1.1.752/1; Sikkim, Rechila Peak, coll. W. Robert, NHMUK 1903.7.1.28/1; Sikhim, NHMUK 20150141/8; Sikkim, Rarhichu, coll. Godwin-Austen, NHMUK 20120110/1 (labelled as *hanleyi*?); Sikhim, Rarhichu, NHMUK 20150143/35 (mixed sample with *Endothyrella minor*); Darjiling, NHMUK 1906.2.2.142/5 (mixed sample with *Endothyrella plectostoma*); Rarhichu, NHMUK 20150134/49; Khasi Hills, leg. Stoliczka, 1880, NHMW 109255/3 (mixed sample with *Endothyrella plectostoma*: NHMW 92593 and *Endothyrella sowerbyi*: NHMW 109254).

##### Diagnosis.

Shell tiny to very small, sinistral, with narrow umbilicus, conical dorsal surface and 7 rows of hairs; callus weak but present; palatal plicae divided, posterior fractions denticle-like; anterior fractions horizontal, straight; lamella straight or very slightly S-shaped, with posterior denticles above and below, and with a lower and an upper plica close to the sutures; lower plica sometimes short, sometimes very long, and reaches the peristome.

##### Measurements

(in mm): D: 4.9–5.7, H: 2.8–3.3 (n = 3, NHMUK 20150134).

##### Differential diagnosis.

See under *Endothyrella macromphalus*, *Endothyrella minor*, *Endothyrella robustistriata* sp. n. and *Endothyrella williamsoni* and Table 5.

##### Description of the genitalia

(Figure 18): Two specimens were anatomically examined. Collection data: Silchar Cachar, F. Ede, coll. Godwin-Austen, NHMUK 1903.7.1.502. Both specimens had several embryos developing in the uterus.

The left ommatophoral retractor passes between penis and vagina. Atrium short, penis long, rather cylindrical, but slowly tapers towards the proximal end; opening the penis was very difficult, not only because of its size, but also due to the age of the specimen; the internal morphology could hardly be seen, although parallel folds forming “pockets” were visible; a little thickening was found near the posterior end of the penis, this could be interpreted as a penial caecum. The slender and relatively long retractor muscle inserts on the proximal end of the penis, slightly in proximal direction from the caecum; epiphallus also slender, slightly longer than the penis; vas deferens long and slim; vagina shorter than the penis and epiphallus combined, it is very thick, with a well-developed vaginal bulb; several short muscle fibres attach the vagina to the body wall and diaphragm; both the gametolytic sac and the diverticulum are very long and slim, although the gametolytic sac is somewhat thickened.

##### Radula

(Figures 19A–B): Radula elongated, but not very slender, central tooth present, laterals approximately 6, standing in straight lines (perpendicular to the central column); marginals approximately 14, although it is difficult to distinguish which are laterals and which are marginals; marginals are placed in oblique rows; central tooth wide-based triangular, smaller than the endocone of the first lateral, but much larger than the ectocone; laterals bicuspid, endo- and ectocones are triangular; marginals usually tricuspid (the endocone has two cusps), but some of the marginals are tetracuspid (both the endocone and the ectocone have two cusps); all cusps pointed, the incision between the innermost two cusps is deep.

##### Distribution.

Most museum samples have been collected in the Sikkim area. Gude received the holotype from Godwin-Austen, and it was said to be collected in the Naga Hills, approximately 600 km from Sikkim. The anatomically examined specimens have been collected from Silchar Cachar, which is located at least 500 km from Sikkim, but not far from the Naga Hills. If the samples from the Naga Hills and from Silchar are correctly labelled, we may expect that the species is widely distributed throughout north-eastern India (see also Figure 11).

#### Endothyrella brahma

Taxon classificationAnimaliaPulmonataPlectopylidae

(Godwin-Austen, 1879)

Figure 16B

Endothyrella brahma 1879a Helix (Plectopylis) brahma Godwin-Austen: Journal of the Asiatic Society of Bengal, 48 (2): 3–4, plate 1, fig 3. [“near Brahmakund, eastern Assam, at 1,000 feet elevation”].
Endothyrella brahma 1887 Helix (Plectopylis) brahma, — Tryon: Manual of Conchology…, 2 (3): 164, Plate 36, figs 35–37.
Endothyrella brahma 1894 *Plectopylis brahma*, — Pilsbry: Manual of Conchology, 2 (9): 145.
Endothyrella brahma 1897d *Plectopylis brahma*, — Gude: Science Gossip, 4: 170–171, figs 63a–c.
Endothyrella brahma 1899c Plectopylis (Chersaecia) brahma, — Gude: Science Gossip, 6: 148.
Endothyrella brahma 1899d Plectopylis (Chersaecia) brahma, — Gude: Science Gossip, 6: 175, 176.
Endothyrella brahma 1914b Plectopylis (Chersaecia) brahma, — Gude: The Fauna of British India…: 74, 113–114, 54a–c.
Endothyrella brahma 1915 Plectopylis (Chersaecia) brahma, — Gude: Records of the Indian Museum, 8: 509, 511.
Endothyrella brahma 1920 Plectopylis (Chersaecia) brahma, — Gude: Proceedings of the Malacological Society of London, 14 (2–3): 63.

##### Types.

Brahamakund, E. Assam, NHMUK 1903.7.1.751. (6 syntypes, Figure 16B).

##### Additional material examined.

Assam, leg. Hungerford, NHMUK 1891.3.17.362–364 (3 specimens); Assam, Brahmakund, coll. Godwin-Austen, NHMUK 20150144/27 (several shells juvenile); Brahmakund, NHMUK 20150145/8.

##### Diagnosis.

Shell very small, sinistral, with narrow umbilicus, depressed conical dorsally, conspicuous radial sculpture without hairs; callus very strong; palatal plicae short, straight, with many small denticles at their posterior ends, standing along a vertical line; lamella oblique, with three horizontal plicae anteriorly, the lowermost is in contact with the lower end of the lamella; besides these anterior plicae, there is a short upper plica above the lamella, and long lower plica close to the lower suture, which runs until the aperture.

##### Measurements

(in mm): D: 8.1–8.2, H: 4.6 (n = 2, type series).

##### Differential diagnosis.

*Endothyrella brahma* can be distinguished from all other *Endothyrella* species by the presence of three parallel, horizontal parietal plicae anterior to the lamella. See also Table 5.

##### Distribution.

The species is known from the type locality only (Figure 10).

#### Endothyrella dolakhaensis

Taxon classificationAnimaliaPulmonataPlectopylidae

Budha & Páll-Gergely sp. n.

http://zoobank.org/B1043A93-8B29-4E3E-A291-5AEED2E3B66F

Figures 9G–H , 14A

##### Type material.

Nepal, Suridobhan, Dolakha, 1023 m, 27.758852°N, 86.197894°E, leg. Budha, P., 03.02.2009., holotype (CDZMTU001, Figure 14A), CDZMTU002 (2 paratypes = shells from the same locality); Nepal, Bhorle, Dolakha, 800 m, 27.696652°N, 86.129583°E, leg. Budha, P., 03.02.2009., 11 paratypes = shells (CDZMTU003).

##### Diagnosis.

Shell small with rather conical dorsal surface; body whorl slightly angulated with five rows of hairs; parietal lamella simple with one or two denticles posteriorly and a plica below; middle palatal plicae divided or almost divided.

##### Description.

Shell very small, sinistral, with somewhat elevated spire and rather conical apex; protoconch elevated from the dorsal surface; colour brownish or greyish; protoconch conspicuously large, consists of 2.25–2.5 whorls (n = 2), very finely, regularly ribbed; teleoconch with clearly visible reticulated sculpture dominated by radial growth lines; sculpture somewhat weaker on the ventral surface; very slender, long periostracal folds (hairs) standing in five spiral lines along the body whorl; whorls 5.25–5.5 (n = 3) moderately bulging, separated by relatively deep suture; umbilicus wide and deep; apertural lip whitish, thin, slightly reflexed; callus also very weak, slightly S-shaped; no fold in the aperture.

One specimen from the type locality was opened. Parietal wall with one rather straight lamella with slight lower arms pointing in both directions; small denticle near the upper end posteriorly, connected to the lamella; two short horizontal plicae under the lamella; palatal wall with six plicae; first slim and short, the second-fifth plicae are divided in the middle and are of the same length; last plica also short, rather straight (Figures 9G–H).

##### Measurements

(in mm): D: 6.5–9.0, H: 4.0–5.0., Wh: 5.5–6.0 (n = 5).

##### Differential diagnosis.

The most similar species are *Endothyrella affinis* and *Endothyrella plectostoma*, which are larger, have a higher spire, and a deeper, narrower umbilicus. *Endothyrella dolakhaensis* sp. n. has a more elevated spire and more rounded body whorl than *Endothyrella angulata* sp. n. Moreover, *Endothyrella dolakhaensis* sp. n. has five rows of periostracal folds, whereas *Endothyrella angulata* sp. n. has four. See also under *Endothyrella macromphalus*, *Endothyrella minor* and *Endothyrella nepalica* sp. n. and Table 5.

##### Etymology.

The new species is named after the district name (Dolakha).

##### Type locality.

Nepal, Suridobhan, Dolakha, 1023 m, 27.758852°N, 86.197894°E.

##### Distribution.

*Endothyrella dolakhaensis* sp. n. is known from two localities in the valley of the Tamakoshi River, Dolakha district, Central Nepal (Figure 15).

#### Endothyrella fultoni

Taxon classificationAnimaliaPulmonataPlectopylidae

(Godwin-Austen, 1892)

**Figure 21.**
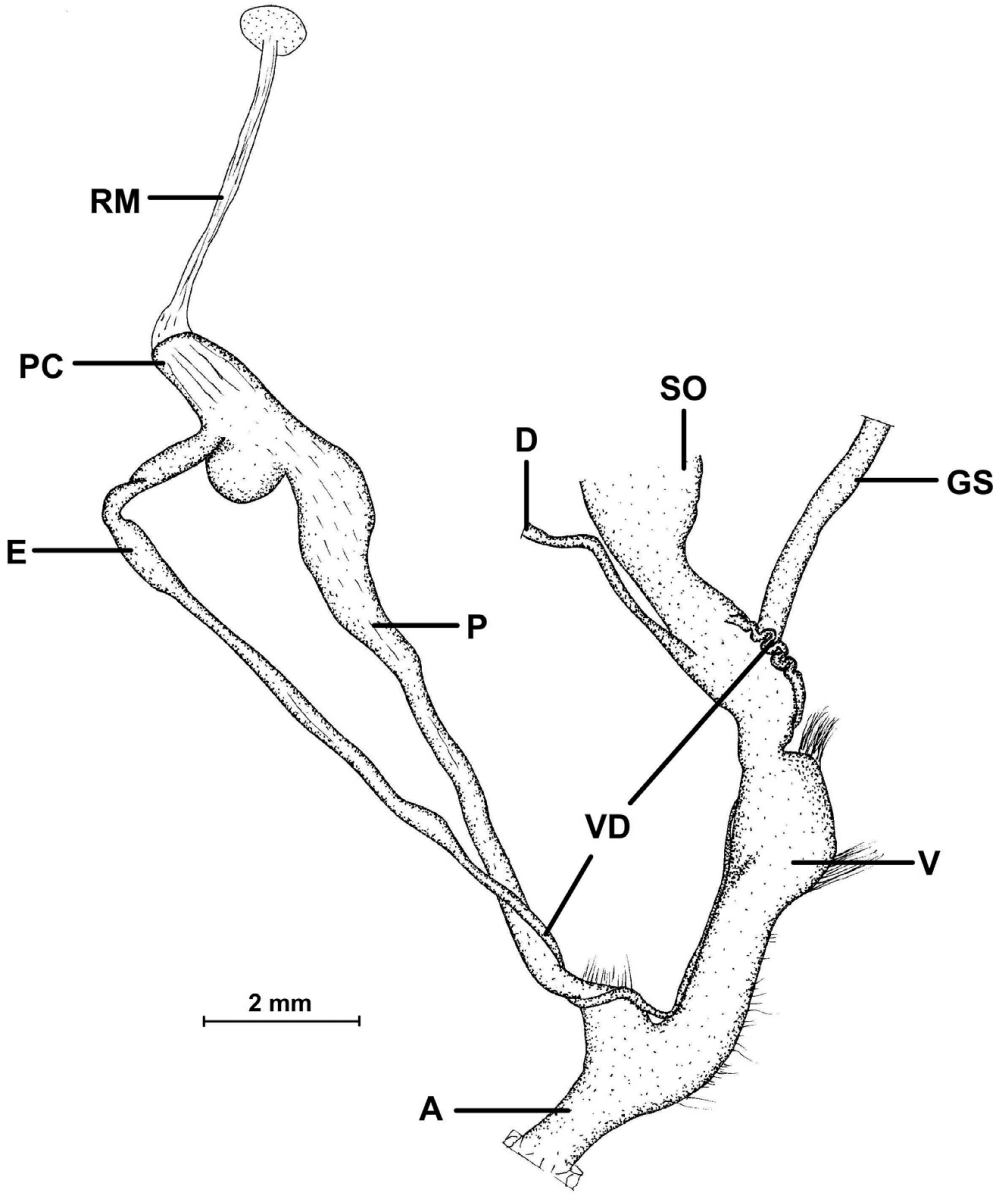
Genital anatomy of *Endothyrella fultoni* (Godwin-Austen, 1892). Locality data: Khasi, leg. Godwin-Austen, NHMUK 1903.7.1.598. Abbreviations: A atrium
D diverticulum
E epiphallus
GS gametolytic sac
P penis
PC penial caecum
RM retractor muscle
SO spermoviduct
V vagina
VD vas deferens.

**Figure 22.**
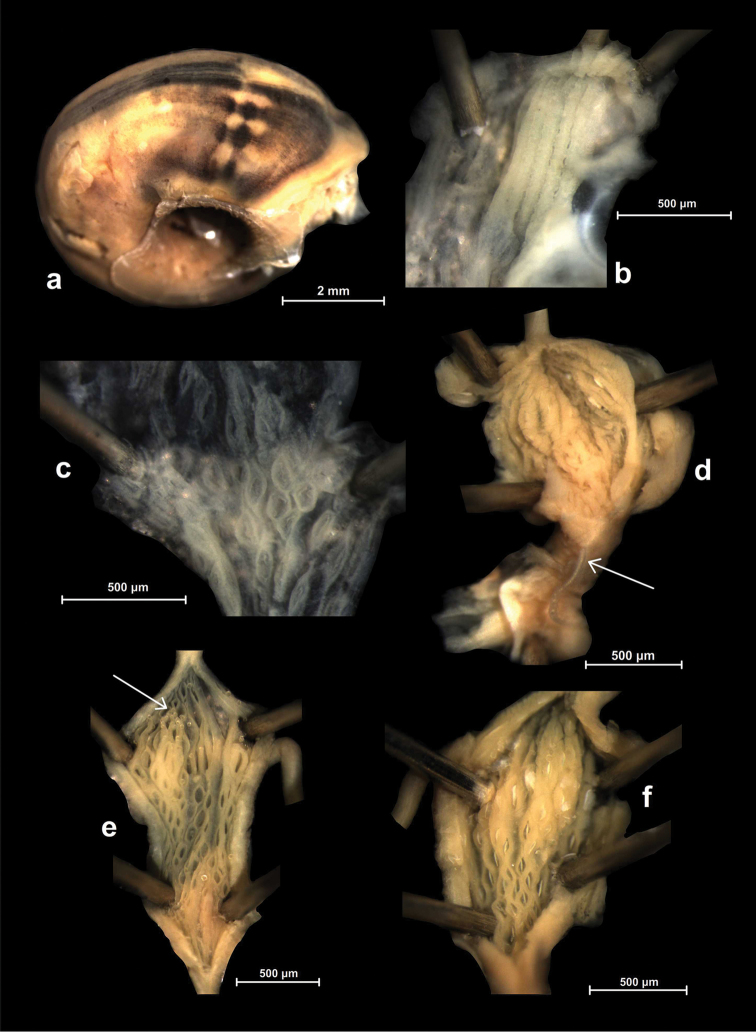
Mantle pattern (**A**) and inner wall of the penis (**C–F**) and the penial caecum (**B**) of *Endothyrella* species. **A** *Endothyrella plectostoma* (Benson, 1836), For locality, see Fig. 19 **B–C** *Endothyrella fultoni* (Godwin-Austen, 1892), for locality see Fig. 21. **D–F** *Endothyrella plectostoma*, for locality, see Fig. 19. Arrow on **D** shows the entering point of the vas deferens to the penis. Arrow on **E** shows rounded calcareous granules. All photos by B. Páll-Gergely.

Figures 12A , 19C–D , 21 , 22B–C

Endothyrella fultoni 1892 Helix (Plectopylis) fultoni Godwin-Austen: The Annals and Magazine of Natural History, 6 (10): 300–301. [“Exact locality unknown. Khasi Hills?”; detailed description on the exactness of the locality on page 301].
Endothyrella fultoni 1893 *Plectopylis fultoni*, — Pilsbry: Manual of Conchology..., 2 (8): 296, 297.
Endothyrella fultoni 1894 *Plectopylis fultoni*, — Pilsbry: Manual of Conchology..., 2 (9): 144, 146, Plate 40, figs 13–15.
Endothyrella fultoni 1896 *Plectopylis fultoni*, — Gude: Science Gossip, 3: 178–179, figs 23a–b.
Endothyrella fultoni 1899c Plectopylis (Endothyra) fultoni, — Gude: Science Gossip, 6: 148.
Endothyrella fultoni 1899d Plectopylis (Endothyra) fultoni, — Gude: Science Gossip, 6: 175, 176.
Endothyrella fultoni 1914b Plectopylis (Endothyra) fultoni, — Gude: The Fauna of British India…: 72, 87–89, figs 36a–b.

##### Types.

Khasi Hills (?) from Fulton, NHMUK 1903.7.1.301. (2 syntypes, Figure 12A).

##### Additional material examined.

Ost-Ind., coll. Gerstenbrandt, NHMW 5954/2; Khasi Hills, Assam, coll. Rušnov, ex coll. Blume, NHMW 71770/R/9 (1 shell); Khasi Hills, leg. Godwin-Austen, NHMW 19599/2; India, Meghalaya, Khasi Hills, leg. Godwin-Austen, Altonaer Museum, ZMH 45907/2; Khasi-Berge, coll. Möllendorff, SMF 150103/3; Assam, Cherrapoonjeh, SMF 150104/4; Ostindien, Assam, coll. C. R. Boettger 1909, SMF 102818/1; Indien, Khasi Berge, coll. Bosch ex coll. Rolle, SMF 172070/3; Khasi Hills, coll. W. Blanford, NHMUK 1906.1.1.737/2; Khasi Hills, coll. Fulton, NHMUK 20150146/3; Assam, Khasi Hills, coll. Trechmann, NHMUK 20150147/2; Assam, Khasi Hills, NHMUK 1892.9.11.9–11/3 (one of them is small juvenile); Assam, Khasi Hills, coll. Lucas, NHMUK 20150148/2; Assam, Khasi Hills, coll. Smith, NHMUK 1937.12.30.13862–13864/3; India, Khasi Hills, coll. Salisbury ex coll. Beddome, NHMUK 20150149/2; Khasi Hills, Assam, coll. Gude, coll. Kennard, NHMUK 20150150/9; Assam, Cherrapoonje, coll. Lucas, NHMUK 20150151/1; no locality, dissected dried animal, NHMUK 20150152/3; no locality, coll. Jousseaume, MNHN 2012-27052/1 juvenile shell.

##### Diagnosis.

Shell middle sized to large, sinistral, with reversed trapezoid shape, narrow umbilicus, angled body whorl, an apex which is elevated from the dorsal surface, and four rows of hairs on the body whorls; callus very strong; 3rd, 4th and 5th palatal plicae are divided in the middle, the others are more or less straight and horizontal; lamella vertical or oblique, with short lower and upper plicae above and below.

##### Measurements

(in mm): D: 19.9–20.3, H: 9.5–10.4 (n = 2, SMF 150103).

##### Differential diagnosis.

*Endothyrella fultoni* is much larger than any other *Endothyrella* species and has a characteristic reversed trapezoid shell shape. See also Table 5.

##### Description of the genitalia

(Figures 21, 22B–C): A single specimen was anatomically examined. Collection data: Khasi, leg. Godwin-Austen, NHMUK 1903.7.1.598. The specimen had some embryos developing in the uterus. The whole body was very fragile, therefore the gametolytic sac and the diverticulum could not be dissected out.

The left ommatophoral retractor passes between penis and vagina. Atrium relatively long; penis long, consists of a longer, slimmer distal and a shorter, more thickened proximal part; at the proximal end of the penis there is a rounded bulb-like thickening (similar to that of some *Gudeodiscus* species, see Páll-Gergely and Asami 2014 and Páll-Gergely et al. 2015); penis internally with honey-comb-like tubercles without calcareous granules (Figure 22C); the somewhat slimmer penial caecum has some (approximately 8) parallel folds inside, which also form minute hollows standing in lines between the folds (Figure 22B); these small pockets may serve for small calcareous granules, although no granules were found; epiphallus enters penis at the basis of the rounded penial thickening; epiphallus relatively short, approximately as long as the proximal, thickened part of the penis; retractor muscle inserts on the proximal end of the penial caecum, it is approximately as long as the proximal part of the penis; vas deferens long and thick, it becomes curly near its insertion to the spermoviductus; vagina shorter than the the half of the penis; it has a vaginal bulb at the middle; two batch of fibres attach the proximal and distal part of the vaginal bulb to the body wall; there are also some longer and more slender muscle fibres attached to the vagina; between the atrium and the vaginal bulb there is a slender, longitudinal thickening on the inner vaginal wall; vaginal bulb internally with fine, irregularly reticulated sculpture; the area of the inner vaginal wall between the bulb and the spermoviductus is roughly reticulated; gametolytic sac relatively thick, the diverticulum is more slender.

##### Radula

(Figure 19C–D): The radula of the only available specimen was very fragile, probably because of the age of the sample; only a fragment of the middle part of the radula could be examined; central tooth present, laterals 14, marginals at least 8; central tooth very long, but somewhat shorter than the endocone of the first lateral, although larger than the ectocones; central tooth elongated triangular with slightly concave marginal line; endocone of the laterals are rather rhomboid, blunt, ectocone pointed triangular; endocones of marginals deformed rhomboid, sometimes oval, showing the sign of becoming bicuspid; ectocones of marginals blunt or pointed triangular.

##### Distribution.

The species is assumed to occur in the Khasi hills (Godwin-Austen 1892) (Figure 11).

#### Endothyrella macromphalus

Taxon classificationAnimaliaPulmonataPlectopylidae

(W. Blanford, 1870)

Figures 23A–B

Endothyrella macromphalus 1870 Helix (Plectopylis) macromphalus W. Blanford: Journal of the Asiatic Society of Bengal, 39 (2): 17–18, Plate 3, fig. 14. [“ad Mairung in montibus Khasi”].
Endothyrella macromphalus 1870–1876 *Helix macromphalus*, — Hanley & Theobald: Conchologia Indica…: Plate 83, figs 8–10.
Endothyrella macromphalus 1875 *Plectopylis macromphalus*, — Godwin-Austen: Proceedings of the Zoological Society of London: 612, 613, Plate 73, figs 1, 1a. [“Darjeeling and N. E. frontier, Bengal. Khási”] (1874, part IV, published in 1875; see Duncan 1937).
Endothyrella macromphalus 1878 Helix (Plectopylis) macromphalus, — Nevill: Hand list of Mollusca in the Indian Museum, Calcutta...: 71.
Endothyrella macromphalus 1879b Helix (Plectopylis) macromphalus, — Godwin-Austen: The Annals and Magazine of Natural History, 5 (4): 163–164.
Endothyrella macromphalus 1887 Helix (Plectopylis) macromphalus, — Tryon: Manual of Conchology…, 2 (3): 160, Plate 34, figs 65–68.
Endothyrella macromphalus 1892 *Plectopylis macromphalus*, — Godwin-Austen: The Annals and Magazine of Natural History, 6 (10): 301.
Endothyrella macromphalus 1893 *Plectopylis macromphalus*, — Pilsbry: Manual of Conchology..., 2 (8): 297.
Endothyrella macromphalus 1894 *Plectopylis macromphalus*, — Pilsbry: Manual of Conchology..., 2 (9): 146.
Endothyrella macromphalus 1897c *Plectopylis macromphalus*, — Gude: Science Gossip, 4: 10–11, figs 46a–b. [“Khasia, Dafla and Naga Hills, in Assam”].
Endothyrella macromphalus 1899c Plectopylis (Endothyra) macromphalus, — Gude: Science Gossip, 6: 147, 148.
Endothyrella macromphalus 1899d Plectopylis (Endothyra) macromphalus, — Gude: Science Gossip, 6: 175, 177.
Endothyrella macromphalus 1914b Plectopylis (Endothyra) macromphalus, — Gude: The Fauna of British India…: 72, 79, figs 29a–b.
Endothyrella macromphalus 1915 Plectopylis (Endothyra) macromphalus, — Gude: Records of the Indian Museum, 8: 507.
Endothyrella macromphalus 1915 Plectopylis (Endothyra) gregorsoni Gude **new synonym**: Records of the Indian Museum, 8: 506–507, Plate 41, figs 2a–d. [“Yamne Valley, Abor Hills”].

##### Types.

**Figure 23.**
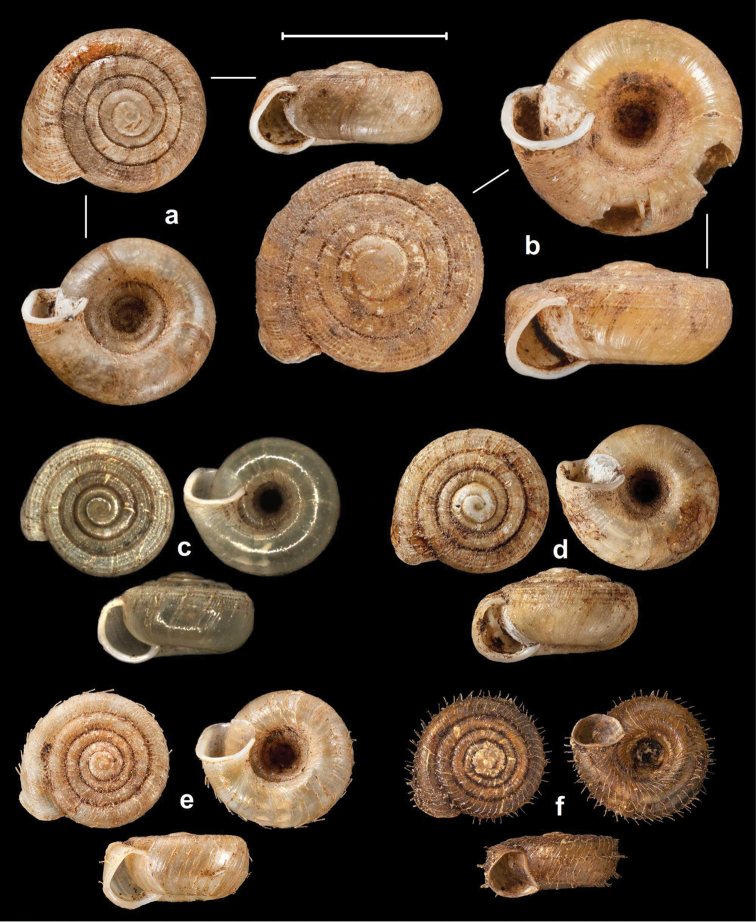
Shells of *Endothyrella* species. **A** *Endothyrella macromphalus* (W. Blanford, 1870), NHMUK 1906.1.1.754. (holotype of *macromphalus*) **B** *Endothyrella macromphalus*, NHMUK 1903.7.1.3124. (holotype of *gregorsoni*) **C** *Endothyrella robustistriata* sp. n., NHMUK 1903.7.1.767. **D** *Endothyrella robustistriata* sp. n., NHMUK 1903.7.1.3453. (holotype) **E** *Endothyrella minor* (Godwin-Austen, 1879), NHMUK 1891.3.17.358–359 (probably syntype) **F** *Endothyrella minor*, Nepal, Lalitpur, Phulchowki Hill, 2308 m, 27.574557°N, 85.400842°E, leg. Budha, P., 04.05.2007. Photos: B. Páll-Gergely (**C**) and H. Taylor (**A, B, D, E, F**). Scale represent 5 mm.

Darjiling, coll. W. Blanford, NHMUK 1906.1.1.754. (holotype of *macromphalus*, Figure 23A); Yamne Valley, Abor Hills, leg. C.F.G. Oakes, R.E., NHMUK 1903.7.1.3124. (holotype of *gregorsoni*, Figure 23B).

##### Additional material examined.

Cherra, leg. Godwin-Austen, NHMUK 20150156/2 (juveniles, mixed sample with *Endothyrella affinis*); Khasi, leg. Stoliczka, 1880.xv.194., NHMW 92589/1 juvenile shell; Khasi Berge, SMF 150102/3 (mixed sample with *Endothyrella minor*); Khasi Berge, coll. Bosch, ex coll. Rolle, SMF 172069/2; Brit. Indien, Toruputu Dfola, 5000', coll. Ehrmann ex coll. Webb, SMF 150101/3; Dafla Hills, Burrail Gorge, coll. Godwin-Austen, NHMUK 1903.7.1.772/10 (4 had, 6 lacked a long lower plica); Khasi Hills, Mairang, coll. W. Blanford, NHMUK 1906.2.2.362/4 (3 lacked, 1 had a long lower plica); Khasi Hills, coll. Godwin-Austen, Figured in Godwin-Austen (1874), NHMUK 1903.7.1.766/9 (2 lacked, 7 had a long lower plica); Mairang, Khasi, NHMUK 1906.1.1.750/1; no locality, NHMUK 20150153/66 (19 shells had a long lower plica, 43 shells lacked, 4 corroded/dirty shells were not examined); Digny, coll. Godwin-Austen, NHMUK 20150154/1 (with long lower plica); Shillong, Khasi, “animal dissected”, NHMUK 1903.7.1.773/1 (with long lower plica); Teria Ghat, coll. Godwin-Austen, NHMUK 20150155/1 (with long lower plica); Toruputu Pk., Dafla Hills, NHMUK 1903.07.01.769/2 (mixed sample with *Endothyrella minor*); no locality, NHMUK 1871.9.23.68/4 (1 with, 3 without a long lower plica).

##### Diagnosis.

Shell very small, sinistral with relatively wide umbilicus, reticulated, almost flat spire (only the apex is elevated) and smooth umbilical side; callus weak, only very slight whitish lime layer is visible; palatal plicae straight, divided or not, lamella with short upper and lower plicae and two posterior denticles, one above and one below; the lower plica might be long (see under Additional material examined).

##### Measurements

(in mm): D: 5.5–8.2.2, H: 2.7–4.2 (n = 13, shells from different samples); the holotype of *Plectopylis gregorsoni* is 7.5 × 3.7 mm.

##### Differential diagnosis.

*Endothyrella macromphalus* has more depressed shells than *Endothyrella blanda*. Moreover, *Endothyrella macromphalus* shells are smooth on the ventral side, whereas most *blanda* shells have hairs, or in case of corroded *Endothyrella blanda* specimens, holes which indicate the hairs’ positions. *Endothyrella dolakhaensis* sp. n. is hairy, has weaker sculpture, and its spire is more elevated than in *Endothyrella macromphalus*. *Endothyrella robustistriata* sp. n. is smaller, has a narrower umbilicus and stronger dorsal sculpture. See also under *Endothyrella williamsoni* and Table 5.

##### Distribution.

*Endothyrella macromphalus* seems to have a wide range including Assam and the Dafla and Khasi Hills. It has been reported from the Naga Hills, but those samples are probably misidentified. *Plectopylis gregorsoni* (treated here as a synonym of *Endothyrella macromphalus*) is recorded from the type locality only (approximately: 28°13.4'N, 95°13.3'E) (Figure 11).

##### Remarks.

The type specimen of *Plectopylis gregorsoni* is very similar to typical *Endothyrella macromphalus* specimens. The main difference is that the palatal plicae are not divided in *gregorsoni*, and the base is less glossy (rather weakly ribbed). In our view these minor difference are not sufficient for species level distinction, especially because *Endothyrella macromphalus* is a relatively variable species inhabiting wide geographical range. Very little is known about the distribution of specimens having divided or undivided palatal plicae. Therefore, until more information becomes available, *Plectopylis gregorsoni* is synonymised with *Endothyrella macromphalus*.

#### Endothyrella minor

Taxon classificationAnimaliaPulmonataPlectopylidae

(Godwin-Austen, 1879)

Figure 23E–F

Endothyrella minor 1870 Helix (Plectopylis) macromphalus var. minor, — W. Blanford, Journal of the Asiatic Society of Bengal, 39 (2): 18. (no formal description presented) [“in valle Rungnu prope Darjiling in Sikkim”].
Endothyrella minor 1879b Helix (Plectopylis) minor Godwin-Austen: The Annals and Magazine of Natural History, 5 (4): 164.
Endothyrella minor 1895 Helix (Plectopylis) minor, — Godwin-Austen: Journal of the Asiatic Society of Bengal, 64: 154, Plate 7, figs 3, 3a.
Endothyrella minor 1897c *Plectopylis minor*, — Gude: Science gossip, 4: 11, figs 47a–k.
Endothyrella minor 1899c Plectopylis (Endothyra) minor, — Gude: Science Gossip, 6: 148.
Endothyrella minor 1899d Plectopylis (Endothyra) minor, — Gude: Science Gossip, 6: 175, 177.
Endothyrella minor 1914b Plectopylis (Endothyra) minor (partim), — Gude: The Fauna of British India…: 73, 75–77, figs 27a–l. [“Sikkim: Darjeeling”, “Rungun Valley”, “India: Naga Hills”, “Laisen Peak, Munipur” (this is the locality of *Endothyrella robustistriata* sp. n.)].
Endothyrella minor 2015 *Endothyrella minor*, — Budha et al., ZooKeys, 492: 18–19.

##### Types.

Darjiling, leg. Stoliczka, coll. Godwin-Austen, NHMUK 1903.07.01.768/10 syntypes. See also remarks.

##### Additional material examined.

Nepal, Lalitpur, Phulchowki Hill, 2308 m, 27.574557°N, 85.400842°E, leg. Budha, P., 04.05.2007., 21 shells (Figure 23F); Nepal, Kathmandu, Chisapani, Shivapuri-Nagarjun National Park, 2361 m, 27.804855°N, 85.436468°E, leg. Budha, P., 11.06.2007., 5 shells; Nepal, Golphubhanjyan, Langtang National Park, Rasuwa, 3340 m, 27.873931°N, 85.757744°E, leg. Budha, P., 10.06.2007., 1 shell; Nepal, Shivapuri-Nagarjun National Park, Deurali, Baghdwar, 2386 m, 27.798318°N, 85.385448°E, leg. Budha, P., 25.04.2008., 1 shell; Nepal, Shivapuri-Nagarjun National Park, Shivapuri Peak, 2707 m, 27.810987°N, 85.383763°E, leg. Budha, P., 24.04.2008., 1 shell; India, Darjiling, leg. Stoliczka, coll. Oberwimmer, NHMW 71640/O/6881 (4 shells); Darjeeling, coll. Rolle, NHMW 71770/R/11 (3 shells); Darjiling, coll. Dr. Stoliczka, 1880, NHMW 91587/20; Darjeeling, coll. Möllendorff, SMF 150112/2; Darjeeling, coll. Webb, SMF 150111/2; Khasi Hills, NHMUK 20150159/3; Sikhim, Rarhichu, NHMUK 20150158/6 (mixed sample with *Endothyrella blanda*); India, Darjeeling, coll. Oldham, NHMUK 20150160/5; India, 1879.12.26.172–177/5; Sikhim, NHMUK 1906.2.2.361/3; Darjeeling, NHMUK 20150161/1 (there is a number “751” on the bottom); Sikkim, NHMUK 1888.12.4.1525(?) (1 specimen); Darjeeling, under stones, 7000', coll. Everest Expedition 9 and 18.03.1924, NHMUK 20150162/5; Khasi Berge, SMF 345110/3 (ex *Endothyrella macromphalus*, SMF 150102); Toruputu Pk., Dafla Hills, NHMUK 1903.07.01.769/4 (mixed sample with *Endothyrella macromphalus*); Darjiling, coll. Hungerford ex coll. Nevill, NHMUK 1891.3.17.358–359 (Figure 23E).

##### Diagnosis.

Shell tiny, sinistral, with relatively narrow umbilicus, flat dorsal surface and four rows of hairs; callus strong; palatal plicae divided; lamella straight or slightly curved, with two denticles posteriorly, one above and one below; lower plica can be short and in some specimens reaching the peristome.

##### Measurements

(in mm): D: 4.9–5.3, H: 2.4–2.6 (n = 3, type series); D: 5–5.1, H: 2.4 (n = 3, SMF 345110); D: 4–5, H: 2–2.5, Wh: 5–5.5 (n = 12, Nepalese specimens).

##### Differential diagnosis.

*Endothyrella minor* is smaller and has weaker keeled body whorl than *Endothyrella angulata* sp. n. Moreover, the first and second rows of the periostracal folds are comparatively at larger distance from each other in *Endothyrella minor* than in *Endothyrella angulata* sp. n. *Endothyrella blanda* has more elevated spire and more hair rows than *Endothyrella minor*. *Endothyrella robustistriata* sp. n. has more elevate spire than *Endothyrella minor* and lacks the hairs on its ventral surface. *Endothyrella macromphalus* is hairless and larger than *Endothyrella minor*, it has a comparatively larger protoconch and a lower (or missing) parietal callus. *Endothyrella minor* is smaller and flatter than *Endothyrella dolakhaensis* sp. n. Moreover, it has a more elevated parietal callus, and has only four rows of hairs (*Endothyrella dolakhaensis* sp. n. has five). See also under *Endothyrella williamsoni* and Table 5.

##### Distribution.

Originally the species was recorded from Darjeeling, Sikkim area. Very similar specimens were found from Central Nepal in the surroundings of Kathmandu (Shivapuri-Nagarjun National Park and Phulchowki hill) and Langtang National Park. Some literature records (Laisen Peak, Naga Hills) are based on misidentified specimens (see Figure 11 and 15).

##### Remarks.

W. Blanford (1870) described Helix (Plectopylis) macromphalus, and while giving information on its locality, he mentioned that “varietas minor” inhabits the Rungun valley near Darjeeling. No description or illustration of “varietas minor” was provided in the paper, therefore the name is not available. Later, Godwin-Austen (1879b) described Helix (Plectopylis) minor from “Darjiling hills” and mentioned those shell “no doubt are referable to Plectopylis macromphalus W. Blf., var. minor”. Blanford’s specimens labelled as *macromphalus minor* have not been found in the collection of the NHM, but the type sample examined and described by Godwin-Austen (NHMUK 1903.07.01.768) was found.

Recent fieldwork in Nepal yielded a few populations in the surroundings of Kathmandu which can be assigned to *Endothyrella minor*. “Typical” specimens of *Endothyrella minor* and Nepalese shells are very similar in terms of size, shell and aperture shape and the morphology of the plicae and lamellae. The only notable difference between these shells is the position of the hair rows on the body whorl. The first row is situated more upper in position (on the upper angle of the body whorl) in the Nepalese shells, whereas in typical shells the first row runs under the angle. Additionally, the distance between the third and fourth rows is smaller in the Nepalese populations.

#### Endothyrella miriensis

Taxon classificationAnimaliaPulmonataPlectopylidae

(Gude, 1915)

Figure 12C

Endothyrella miriensis 1915 Plectopylis (Endothyra) miriensis Gude: Records of the Indian Museum, 8: 507–508, Plate 41, figs 3a–d. [“Miri Hills, Upper Assam”].

##### Types.

Miri Hills, leg. C.F.G. Oakes, R.E., NHMUK 1903.7.1.3205. (4 syntypes, Figure 12C)

##### Diagnosis.

Shell small, sinistral, with very slightly elevated spire, relatively wide umbilicus, and conspicuous spiral sculpture; callus moderately strong, palatal plicae slightly oblique, connected by a vertical ridge; lamella almost straight, with anteriorly elongated upper and lower ends and small denticles on the posterior side, one above and one below.

##### Measurements

(in mm): D: 12.1–12.3, H: 5.3–5.4 (n = 2, type series).

##### Differential diagnosis.

The unique spiral sculpture, which is very prominent on the ventral side as well, distinguishes *Endothyrella miriensis* from all congeners. See also Table 5.

##### Distribution.

The species is known from the type locality only (Figure 10).

#### Endothyrella nepalica

Taxon classificationAnimaliaPulmonataPlectopylidae

Budha & Páll-Gergely sp. n.

http://zoobank.org/1ED614EA-455F-4507-B1EF-F5129052F4E0

**Figure 24.**
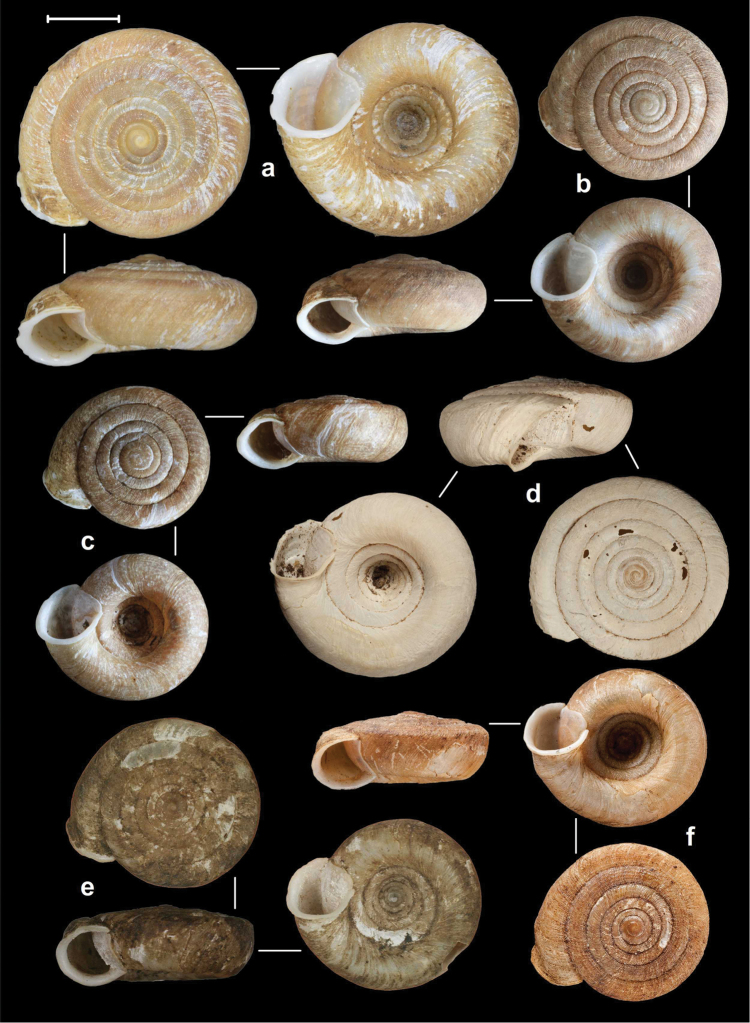
Shells of *Endothyrella* species. **A** *Endothyrella nepalica* Budha & Páll-Gergely, sp. n., paratype, same data as on Fig. 6E **B** *Endothyrella nepalica* Budha & Páll-Gergely, sp. n., holotype **C** *Endothyrella nepalica* Budha & Páll-Gergely, sp. n., paratype, NMBE 527538 **D** *Endothyrella pinacis* (Benson, 1859), (holotype of *pinacis*) **E** *Endothyrella pinacis* (holotype of *Helix pettos*) **F** *Endothyrella pinacis*, NHMUK 1906.2.2.143. Photos: B. Páll-Gergely (**A**), E. Bochud (**B, C**), J. Gundry (**D**), Ch. Zorn (**E**), H. Taylor (**F**). Scale represent 5 mm.

**Figure 25.**
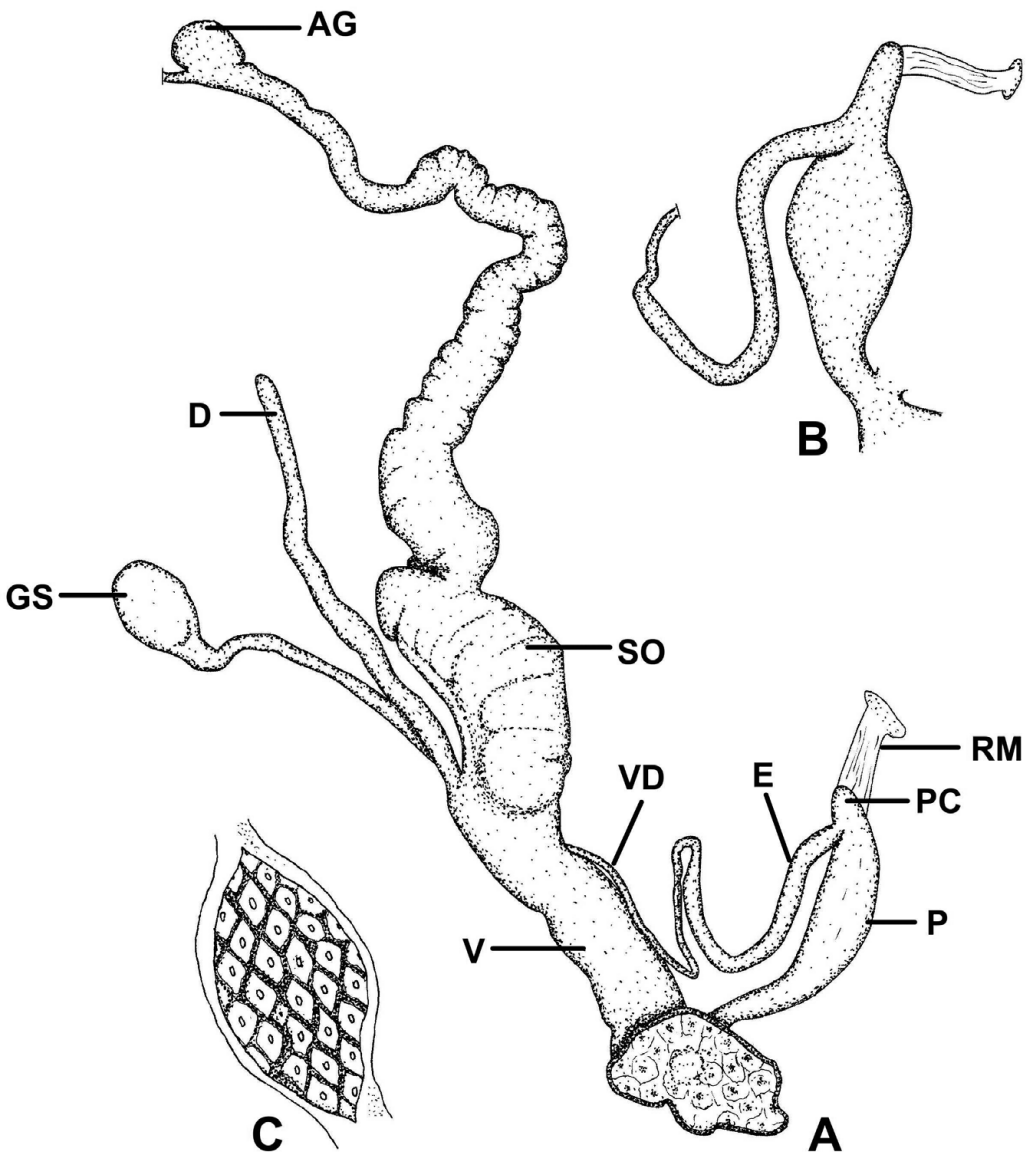
Genital anatomy of *Endothyrella nepalica* sp. n. **A** Specimen from Godawari **B, C** penis of a specimen form Balaju. Diagrammatic. Abbreviations: AG albumen gland
D diverticulum
E epiphallus
GS gametolytic sac
P penis
PC penial caecum
RM retractor muscle
SO spermoviduct
V vagina
VD vas deferens.

Figures 6E , 8A–C , 9C–F , 24A–C , 25

Endothyrella nepalica 2015 *Endothyrella affinis*, — Budha et al., ZooKeys, 492: 18.

##### Type material.

Champadevi, Kirtipur, Kathmandu District, 1326–1500 m, 27.654868°N, 85.244084°E, leg. Budha, P., 02.10.2010., holotype (CDZMTU005.1), paratypes CDZMTU005.2–16 (15 shells), CDZMTU005P (2 paratypes = specimens dissected and preserved, 3 dry shells = paratypes, 2 juvenile shells = not paratype); W-Nepal, Dhaulagiri Zone, Myagdi District, Annapurna Conservation Area, right side of Kali Gandaki valley, 300 m NNW of Suke Bagar village along “Tatopani-Dana” track, 1430 m alt., 14.05.1996., leg. A. Kuznetsov, WM/10 paratypes; Nepal, Kathmandu Valley, NW end of Kathmandu, middle part of S slope of Swoyambhunath Hill, in dry oak forest, 1500 m, 25.04.1995, leg A. Kuznetsov, WM/4 sinistral and 1 dextral paratypes; W Nepal, Daulagiri zone, Hyagdi distr., Annapurna NP., right side of Kali Gandaki v., NNW od Suke Bagar, Tatop, leg. A. Kuznetsov, 14.05.1996., ex coll. W. Maassen, HNHM 95867/1 paratype (labelled as paratype of “*Plectopylis nepalensis* Schileyko and Kuznetsov”); Nepal, Swoyambhunath, Kathmandu District, 1366 m, 27.716971N, 85.289386 E, leg. Budha, P., 05.09.2008, CDZMTU006 (24 paratypes = shells); Siddha Cave, Tanahun District, 600 m, 27.94718°N, 84.421338°E, leg. Budha, P., 24.10.2008, CDZMTU004, CDZMTU007 (11 paratypes = shells, and one juvenile shell, which is not paratype) (Figs 6E, 24A); Dhunche, Rasuwa, 1985 m, 28.1092°N, 85.2916°E, leg. Budha, P., 31.05.2007., CDZMTU008 (2 shell = paratypes, and one damaged shell which is not paratype); Balaju, Kathmandu District, 1356 m, 27.741173°N, 85.293763°E, leg. Budha, P., 04.01.2009., CDZMTU009 (8 paratypes = shells), CDZMTU009P (2 paratypes = specimens preserved, 4 dry shells = paratypes); Mahadevsthan, Thankot, Kathmandu District 1500 m, 27.683366°N, 85.213834°E, leg. Budha, P., 06.02.2007., CDZMTU010 (25 paratypes = shells), CDZMTU010P 2 paratypes = specimens preserved, 4 dry shells = paratype, 5 juvenile shells = not paratypes); Arjewa, Baglung, 900 m, 28.154393°N, 83.630703°E, leg. Budha, P., 13.09.2006., CDZMTU011 (14 paratypes = shells, one juvenile shells = not paratype); Majhbeni, Parbat, 700 m, 28.205708°N, 83.674605°E, leg. Budha, P., 13.09.2006., CDZMTU012 (9 paratypes = shells, 6 juvenile/damaged shells = not paratypes); Sirsuwa, Parbat District, 780 m, 28.136478°N, 83.642135°E, leg. Budha, P., 13.09.2006., CDZMTU013 (6 paratypes = shells); Foksing, Parbat District, 790 m, 28.093252°N, 83.604283°E, leg. Budha, P., 11.06.2006., CDZMTU014 (11 paratypes = shells, 2 juvenile shells 7 not paratypes); Godawari, Lalitpur, 1868 m, 27.94718°N, 84.421338°E, leg. Budha, P., 01.10.2008., CDZMTU015a (1 paratype); Annapurna Conservation Area, Tatopani, 1282 m, 28.495172°N, 83.628883°E, leg. Budha, P., 01.10.2008., CDZMTU016 2 (2 paratypes = shells); Godawari, Lalitpur, 1575 m, 27.596459°N, 85.389432°E, leg. Budha, P., 30.06.2007., CDZMTU015b (1 paratype = shell); Ridi, Gulmi, 832 m, 27.945621°N, 83.43215°E, leg. Budha, P., 30.06.2007., CDZMTU017 (5 paratypes = shells); Godawari Botanical Garden, Lalitpur, 1453 m, 27.596671°N, 85.381758°E, leg. Budha, P., 03.09.2008., CDZMTU015c (50 paratypes = shells); Nepal, Pokhara, Khare, 1520 m alt., 28.2860°N, 83.8472°E, leg. C. Huber, 18.03.1991, NMBE 527538/1 paratype (Figure 24C).

##### Diagnosis.

A small to middle-sized, hairless species with domed dorsal surface and rounded body whorl; parietal lamella simple with one or two denticles posteriorly and sometimes a plica below the lamella, middle palatal plicae divided or almost divided.

##### Description.

Shell very small to small, sinistral, with somewhat elevated spire and domed dorsal surface; protoconch slightly elevates from the dorsal surface; usually brownish but sometimes turns into yellowish; protoconch consists of 1.5–1.75 whorls, very finely, regularly ribbed; teleoconch with very weak, irregular growth lines on the ventral surface and fine reticulated sculpture on the dorsal surface; in high magnification the surface is covered by flat periostracal folds; no spirally arranged large deciduous folds found; whorls 5.5–6.25, moderately bulging, separated by relatively deep suture; umbilicus wide and deep, whorls almost flat inside, resulting in an funnel-like shape, apertural lip whitish, rather thin, slightly reflexed; callus inconspicuous, but present, slightly S-shaped; no fold in the aperture.

Ten specimens were opened from different populations. Parietal wall with one slightly curved lamella with arms pointing in the direction of the aperture; lower end on the lamella more conspicuously curved than the upper end; two small denticles above and below posteriorly of the lamella (exceptionally, the lower one is missing); in some populations (e.g. Majhbeni – Parbat District, Champadevi – Kathmandu District and Siddha Cave – Tanahu District) with short plica under the lamella; palatal wall with six plicae; first slim and short, parallel with the suture; second plica is the longest, it shows a tendency towards dividing in the middle, but the two parts always fused; third, fourth and fifth plicae usually divided (third one sometimes not); last plica short, slightly curved with arms pointing in the direction of the lower suture (Figures 9C–F).

##### Measurements

(in mm): D: 8.2–14.9, H: 4.0–6.0, Wh: 5.5–7.5 (n = 35, different populations).

##### Differential diagnosis.

*Endothyrella nepalica* sp. n. is usually larger than *Endothyrella angulata* sp. n., it has a domed dorsal surface, rounded body whorl and lacks hairs standing in spiral rows, whereas *Endothyrella angulata* sp. n. has a flat dorsal surface, shouldered body whorl and has hairs which are arranged in spiral rows. *Endothyrella dolakhaensis* sp. n. differs from *Endothyrella nepalica* sp. n. by the usually smaller size, fewer whorls, stronger sculpture, comparatively larger protoconch, conical dorsal surface, slightly angulated body whorl and the presence of hairs standing in five spiral lines. For comparison with *Endothyrella oakesi* and *Endothyrella pinacis*, see under those species. See also Table 5.

##### Description of the genitalia

(Figures 25A–C): Three specimens from three populations were anatomically examined (Champadevi, Balaju of Kathmandu District and Godawari Botanical Garden, Lalitpur District). Penis short, narrow distally and slowly tapers toward the proximal end; internal surface with several tubercles including minute calcareous hooks; epiphallus slender, cylindrical, longer than the penis, it enters penis laterally; penial caecum very short, blunt, cylindrical, with a short retractor muscle attached at its proximal end; vas deferens thin and nearly 1.5 times longer than epiphallus, convoluted before connection to prostate; vagina shorter than the penis with well-developed vaginal bulb; gametolytic sac very thin throughout and ends into a small rounded sac; there is a slender diverticulum running parallel with the gametolytic sac; it is as long as the gametolytic sac.

##### Etymology.

The name *nepalica* refers to the country (Nepal) where the new species lives.

##### Type locality.

Champadevi, Kirtipur, Kathmandu District, Nepal, 1326–1500 m, 27.654868°N, 85.244084°E.

##### Distribution.

*Endothyrella nepalica* sp. n. inhabits a relatively large area in western and central Nepal (Figure 15).

##### Remarks.

Schileyko (1999) figured a shell from the “SW slope of Swayambhunat (= Swoyambhunath) hill, Kathmandu valley, Nepal” (Fig. 594.). The figured specimen is probably *Endothyrella nepalica* sp. n., but the drawing is not sufficient for identification.

#### Endothyrella oakesi

Taxon classificationAnimaliaPulmonataPlectopylidae

(Gude, 1915)

Figure 16A

Endothyrella oakesi 1915 Plectopylis (Endothyra) oakesi Gude: Records of the Indian Museum, 8: 505–506, Plate 41, Figs 1a–d. [“Yamne Valley, Abor Hills and Sibbum”, “between Riu and Singging, on the Dihang River”].

##### Types.

Yamne Valley, Abor Hills, leg. C.F.G. Oakes, R.E., NHMUK 1903.7.1.3125 (5 syntypes, Figure 16A).

##### Additional material examined.

Sibbum, Abor, NHMUK, coll. Godwin-Austen, NHMUK 20150157/1; Abor Hills, “exact position not known”, below alt. 3000' between lat. 28°15'+29°15', long. 94°50'+95°10', leg. Oakes, coll. Godwin-Austen, NHMUK 1903.7.1.3125/1.

##### Diagnosis.

Shell small, sinistral, with wide umbilicus, and slightly domed dorsal surface; callus strong, palatal plicae complicated, their anterior part is horizontal, but the posterior part vertical; there are several short horizontal plicae between posterior parts of the palatal plicae; lamella almost straight with posteriorly elongated upper end, and sometimes with a long lower plica which reaches the aperture.

##### Measurements

(in mm): D: 11.7–12.5, H: 4.7–5.5 (n = 3, type series).

##### Differential diagnosis.

*Endothyrella nepalica* sp. n. also has simpler palatal plicae than those of *Endothyrella oakesi*. Moreover, *Endothyrella nepalica* sp. n. has a flatter shell and a less descending aperture. See also under *Endothyrella pinacis* and Table 5.

##### Distribution.

This species was reported only from the localities mentioned in the original description (Yamne Valley, Abor Hills and Sibbum”, “between Riu and Singging, on the Dihang River”) (Figure 10).

##### Remarks.

Three specimens (two adults and a juvenile) of the type lot of *Endothyrella oakesi* were opened (probably by Gude). The long lower parietal plica, described as characteristic feature of this species, is present only in one specimen. In face of this, *Endothyrella oakesi* seems to be a distinct species on the basis of the palatal plicae and shell shape.

#### Endothyrella pinacis

Taxon classificationAnimaliaPulmonataPlectopylidae

(Benson, 1859)

Figures 24D–F

Endothyrella pinacis 1859 *Helix pinacis* Benson: The Annals and Magazine of Natural History, 3 (3): 268–269. [“Habitat raro in regione Sikkim in valle Rungun (4000 ped.), necnon prope Pankabari (1000 ped. alt.)”].
Endothyrella pinacis 1860 Helix (Plectopylis) pinacis, — Benson: The Annals and Magazine of Natural History, 3 (5): 243–247. [“Darjiling and the Khasia Hills”].
Endothyrella pinacis 1868 *Helix pinacis*, — Pfeiffer: Monographia Heliceorum Viventium…, 5: 417.
Endothyrella pinacis 1868 Helix (Corilla) pettos Martens: Malakozoologische Blätter, 15: 158.
Endothyrella pinacis 1869 *Helix pettos*, — Pfeiffer: Novitates conchologicae…: 462–463.
Endothyrella pinacis 1872 *Helix pinacis*, — Hanley & Theobald: Conchologia Indica…: 7, 36, Plate 13, fig. 5, Plate 84, figs 1–4. [“Sikkim (Rungun, and near Pankabari)”].
Endothyrella pinacis 1875 *Plectopylis pettos*, — Godwin-Austen: Proceedings of the Zoological Society of London, 612. [“Himalaya?”].
Endothyrella pinacis 1875 Helix (Plectopylis) pinacis, — Godwin-Austen: Proceedings of the Zoological Society of London: 612, 613, plate 74, fig. 1. (1874, part IV, published in 1875; see Duncan 1937).
Endothyrella pinacis 1878 Helix (Plectopylis) pinacis, — Nevill: Hand list of Mollusca in the Indian Museum…: 71.
Endothyrella pinacis 1879b Helix (Plectopylis) pinacis, — Godwin-Austen: The Annals and Magazine of Natural History, 5 (4): 163.
Endothyrella pinacis 1887 Helix (Atopa) pettos, — Tryon: Manual of Conchology…, 2 (3): 156, Plate 34, figs 36–38.
Endothyrella pinacis 1887 Helix (Plectopylis) pinacis, — Tryon: Manual of Conchology…2(3)159–160, Plate 34, figs 53–55.
Endothyrella pinacis 1894 *Plectopylis pinacis*, — Pilsbry: Manual of Conchology..., 2 (9): 144, 146.
Endothyrella pinacis 1894 *Plectopylis pettos*, — Pilsbry: Manual of Conchology..., 2 (9): 146.
Endothyrella pinacis 1895 *Plectopylis pinacis*, — Godwin-Austen: Journal of the Asiatic Society of Bengal, 64: 154, Plate 7, figs 2, 2a.
Endothyrella pinacis 1897a *Plectopylis pinacis*, — Gude: Science Gossip, 3: 206, figs 32a–d.
Endothyrella pinacis 1897a Helix (Corilla) pettos = *Plectopylis pinacis*, — Gude: Science Gossip, 3: 206.
Endothyrella pinacis 1899c Plectopylis (Endothyra) pinacis, — Gude: Science Gossip, 6: 147, 148.
Endothyrella pinacis 1899c Plectopylis (Endothyra) pettos (under *pinacis*), — Gude: Science Gossip, 6: 148.
Endothyrella pinacis 1899d Plectopylis (Endothyra) pinacis, — Gude: Science Gossip, 6: 175, 177.
Endothyrella pinacis 1899d *pettos*, — Gude: Science Gossip, 6: 177.
Endothyrella pinacis 1907 *Plectopylis pinacis*, — Godwin-Austen: Land and freshwater Mollusca of India…: 203–204.
Endothyrella pinacis 1914b Plectopylis (Endothyra) pinacis, — Gude: The Fauna of British India…, 72, 86–87, figs 35a–d. [“Sikkim : Darjeeling”, “Rungun, Pankabari”, “Rungmaval”, “Damsang”].
Endothyrella pinacis 1914b Plectopylis (Endothyra) pinacis, — Gude: The Fauna of British India…: 72, 86.
Endothyrella pinacis 1915 Plectopylis (Endothyra) pinacis, — Gude: Records of the Indian Museum, 8: 506, 508.

##### Types.

Sikkim, coll. Benson, UMZC 102755 (holotype of *Helix pinacis*, Figure 24D); Himalaya, ZMB/MOLL 17905 (holotype of *Helix pettos*, Figure 24E).

##### Additional material examined.

India, West Bengal, Darjeeling District, Lopchu + Ghum, coll. Topál, 21–22.04.1967, locality code: 869, HNHM 98848/2; Darjiling, coll. Dr. Stoliczka, 1880, NHMW 92590/7; Sikkim, coll. Möllendorff, SMF 150110/6 (3 of them juvenile); Darjeeling, coll. Bosch, ex coll. Rolle, SMF 172075/2; Darjiling, figured in Godwin-Austen (1874), coll. Godwin-Austen, NHMUK 1903.7.1.746/5; Darjiling, coll. W. Blanford, NHMUK 1860.6.27.14 (1 specimen); Kungna valy. (?) Sikm., NHMUK 20150163/2; Darjiling, NHMUK 1906.2.2.143/2 (Figure 24F); Damsang Peak, Daling Hills, coll. Godwin-Austen, NHMUK 20150164/26 (several of the juvenile); Sikkim, Rarhichu, NHMUK 20150165/5; Rechila Peak, coll. Godwin-Austen, NHMUK20150167/1; Darjiling, NHMUK 1888.12.4.1524/1; Darjeeling, 5000', coll. Everest Expedition 1924, NHMUK 20150168/1; Rarkichu, Sikkim, coll. Godwin-Austen, NHMUK 20150166/1.

##### Diagnosis.

Shell very small to small, sinistral, hairless, with wide umbilicus and slightly angulated body whorl; callus strong, palatal plicae short and oblique, lamella rather straight with anteriorly elongated upper and lower ends, and posteriorly elongated upper end; there are two denticles on the posterior side of the lamella, one above and one below, the lower one might be in contact with the lamella.

##### Measurements

(in mm): D: 13.6–14.1, H: 5.9–6.1 (n = 3, SMF 150110).

##### Differential diagnosis.

*Endothyrella angulata* sp. n. is usually smaller than *Endothyrella pinacis*, it has a stronger keel and has weaker spiral lines on the ventral side of the shell, which are clearly visible in *Endothyrella pinacis*. The most similar species is *Endothyrella nepalica* sp. n., which nevertheless has a higher spire and rounded whorls, whereas *Endothyrella pinacis* has shouldered whorls and nearly flat dorsal surface. The ventral surface of the two species is similar, but *Endothyrella pinacis* has slender hairs standing in 3 lines, which is missing in *Endothyrella nepalica* sp. n. According to previous studies (Godwin-Austen 1889–1914, Schileyko 1999) *Endothyrella pinacis* has no diverticulum, but in all *Endothyrella nepalica* sp. n. we dissected that organ was present. *Endothyrella oakesi* is similar to *Endothyrella pinacis*, but has much more complicated palatal plicae, more descending aperture, differently shaped umbilicus and rounded body whorl. See also Table 5.

##### Anatomy.

The anatomy of *Endothyrella pinacis* was described by Godwin-Austen (1889–1914) and Schileyko (1999). According to these descriptions, only the gametolytic sac is present and the diverticulum is missing. The penial caecum seems to be missing, although none of these drawings show this part clearly. Other features of the genitalia (penis shape, internal wall of the penis, vagina) are similar to those of *Endothyrella nepalica* sp. n.

##### Radula.

Stoliczka (1871) mentioned that the central tooth is larger than that of *Plectopylis achatina* (= *bensoni*), and that its shape is similar to that of the laterals. Godwin-Austen (1889–1914) gave an accurate description and drawings of the teeth. According to his drawings the morphology of the teeth of *Endothyrella pinacis* is typical for the genus *Endothyrella*, i.e. the central tooth is larger than the ectocones of the first laterals, and the marginals are tricuspid with deep incisions between the two innermost cusps.

##### Distribution.

All museum samples examined were collected from Sikkim. Benson’s (1860) locality in the Khasi Hills is probably incorrect (Figure 11).

#### Endothyrella plectostoma

Taxon classificationAnimaliaPulmonataPlectopylidae

(Benson, 1836)

**Figure 26.**
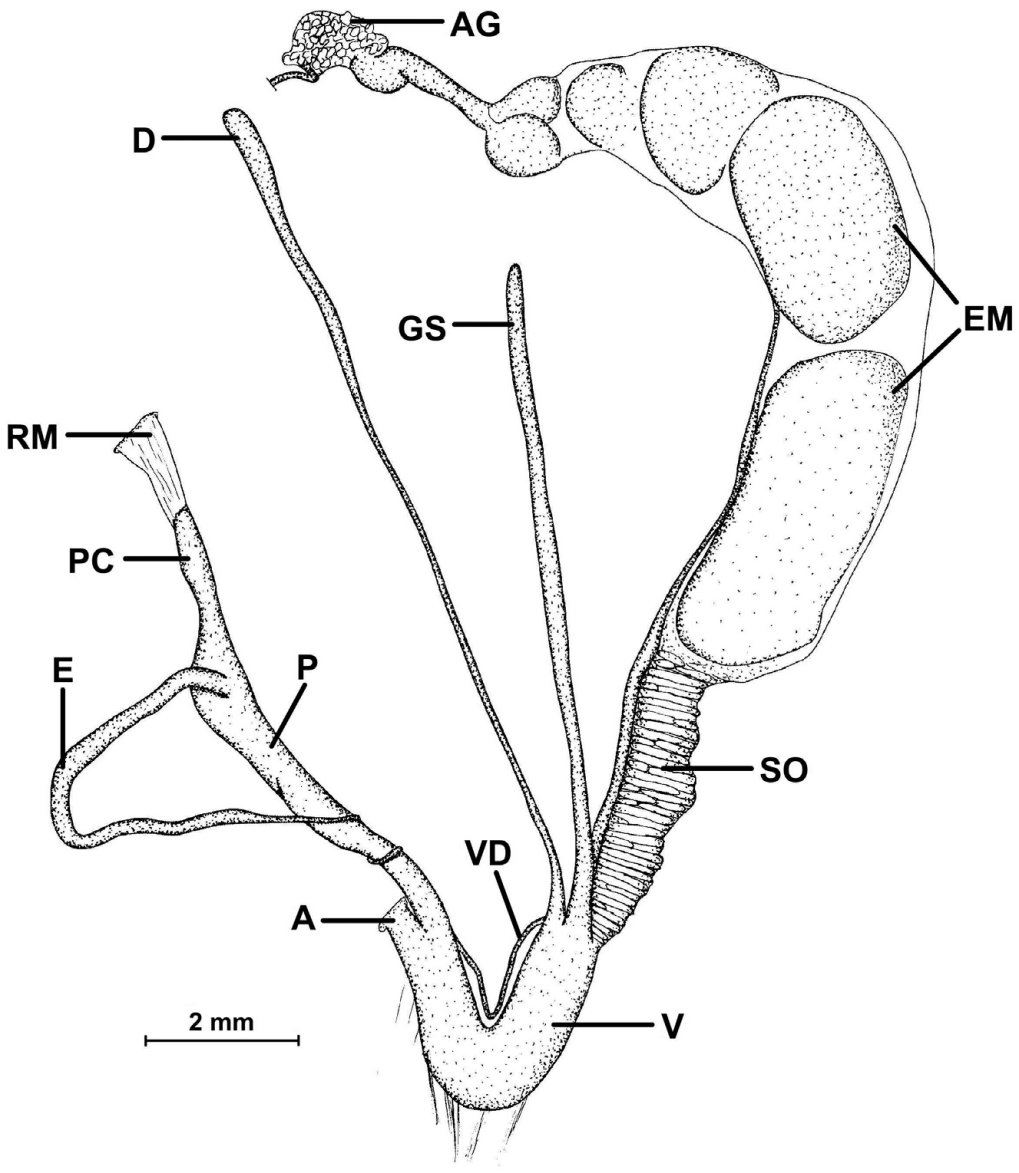
Genital anatomy of *Endothyrella plectostoma* (Benson, 1836). For locality, see Figs 19E–F. Abbreviations: A atrium
AG albumen gland
D diverticulum
E epiphallus
EM embryos
GS gametolytic sac
P penis
PC penial caecum
RM retractor muscle
SO spermoviduct
V vagina
VD vas deferens.

Figures 6D , 13A–B , 19E–F , 20D–E , 22A , 22D–F , 26

Endothyrella plectostoma 1836 Helix (Helicodonta) plectostoma Benson: Journal of the Asiatic Society of Bengal, 5: 351. [not specified. “North-East Frontier of Bengal” (in the title)].
Endothyrella plectostoma 1848 *Helix plectostoma*, — Pfeiffer, Martini & Chemnitz, 1(12): 367, Plate 64, figs 19–21.
Endothyrella plectostoma 1854 *Helix plectostoma*, — Reeve: Conchologia Iconica 7, species 782.
Endothyrella plectostoma 1860 *Helix plectostoma*, — Benson: The Annals and Magazine of Natural History, 3 (5): 247.
Endothyrella plectostoma 1865 *Helix plectostoma*, — W. Blanford: Journal of the Asiatic Society of Bengal 34 (2): 94. [“...the Himalayan and Khasi *Helix plectostoma*, Bens. abounded south of the town of Bassein in several places, Pyema Khyoung, Long Island, &c. It was also found by Captain Ingram in Arakan, near Tongoop.”].
Endothyrella plectostoma 1872 Helix (Plectopylis) plectostoma, — Hanley & Theobald: Conchologia Indica…: 7, Plate 13, fig. 2. [“Darjiling and Khasia Hills”].
Endothyrella plectostoma 1875 *Plectopylis plectostoma*, — Godwin-Austen: Proceedings of the Zoological Society of London: 612–613, Plate 73, figs 2–2a. (1874, part IV, published in 1875; see Duncan 1937).
Endothyrella plectostoma 1878 Helix (Plectopylis) plectostoma, — Nevill: Hand list of Mollusca in the Indian Museum…: 1: 71. [“Nágá Hills”, “Bassein, &c., Pegu”, “Sylhet”, “Arakan Hills”, “Khasi Hills”, “Darjeeling”].
Endothyrella plectostoma 1887 Helix (Plectopylis) plectostoma, — Tryon: Manual of Conchology…, 2 (3): 160–161, Plate 34, figs 69–70.
Endothyrella plectostoma 1894 *Plectopylis plectostoma*, — Pilsbry: Manual of Conchology..., 2 (9): 146.
Endothyrella plectostoma 1897b *Plectopylis plectostoma*, — Gude: Science Gossip, 3: 274–275, figs 39a–7c. [“Darjeeling”, “Burma— Bassein and Arakan; Assam — Sylhet, Khasia and Naga Hills”, “Dafla Hills in Assam”].
Endothyrella plectostoma 1899c Plectopylis (Endothyra) plectostoma, — Gude: Science Gossip, 6: 148, 149.
Endothyrella plectostoma 1899d Plectopylis (Endothyra) plectostoma, — Gude: Science Gossip, 6: 175, 177.
Endothyrella plectostoma 1914b Plectopylis (Endothyra) plectostoma, — Gude: The Fauna of British India…: 72, 73, 75, 81–83, figs 31a–c. [“Naga Hills”, “Dafla Hills, Khasi Hills”, “Burma: Arakan Hills”, “Tongoop”, “Bassein: Pegu”, “Sylhet”, “Sikkim : Darjeeling”].
Endothyrella plectostoma 1922 Plectopylis (Endothyra) plectostoma, — Ehrmann: Sitzungsberichte der Naturforschender Gesellschaft zu Leipzig, 45–48: 8–10.
Endothyrella plectostoma 1960 Plectopylis (Endothyrella) plectostoma, — Zilch: Handbuch der Paläozoologie, 6 (2): fig. 2092.

##### Types:.

Darjeeling, coll. MacAndrew ex coll. Benson, UMZC 102160 (7 syntypes of *plectostoma*); Darjeeling, coll. MacAndrew ex coll. Benson, UMZC 102155 (1 syntype of *plectostoma*, Figure 13A); Bengal, coll. MacAndrew ex coll. Benson, UMZC 102156 (3 syntypes of *plectostoma*).

##### Additional material examined.

Indien, Khasi Hills, ex coll. Oberwimmer, NHMSB 122805–122810/5; India, Meghalaya, Khasi Hills, Altonaer Museum, ZMH 45909/4; Assam, coll. Steenberg, ZMUC-GAS-1812/2; Naraindher, Cachar, Ede, coll. Godwin-Austen, NHMUK 1903.7.1.1666/15 (several of them are juveniles); Darjiling, coll. W. Blanford, NHMUK 1860.6.27.10/2; India, NHMUK 20150169/1; Teria Ghat, NHMUK 1888.12.4.1536–1540/5; Pegu, coll. Godwin-Austen, NHMUK 1909.3.15.92/7; Naga Hills, coll. Godwin-Austen, NHMUK 1903.7.1.760/3; Pegu, Arakan, NHMUK 1903.7.1.758/3; Arakan, coll. W. Blanford, NHMUK 1909.3.15.60/3; Assam, Khasi Hills, coll. Salisbury ex coll. Beddome, NHMUK20150170/3; Lhota Naga, coll. Chennell, NHMUK 1903.7.1.759/10; Saddia, E Assam, coll. Godwin-Austen, NHMUK 1903.7.1.761/8; Picholanulla, Durrang, Assam, coll. Godwin-Austen, NHMUK 1903.7.1.763/1; Khasi Hills, coll. W. Blanford, NHMUK 1906.2.2.356.1–3 (3 shells; mixed sample with *Endothyrella sowerbyi*: 1906.2.2.356.4); Arakan, coll. W. Blanford, NHMUK 1906.2.2.355/4; India, NHMUK 20150171 (6 specimens); Darjiling, NHMUK 1906.2.2.142/1 (mixed sample with *Endothyrella blanda*); Shiroifurar, Lahupa Naga, coll. Godwin-Austen, NHMUK 1903.6.1.762/1; India, NHMUK 71.9.23.206/3; no data, coll. W. Blanford, NHMUK 20150172/2; Munipur valley, Bishenpur, west side, NHMUK 20150173/25 (several of them are juvenile shells); N. Cachar, coll. Godwin-Austen, NHMUK 20150174/2; Teria Ghat, coll. Godwin-Austen, NHMUK 20150175/1; Cherra, Khasi Hills, Assam, coll. Godwin-Austen, NHMUK 20150176/25; Dunsiri valley, coll. Godwin-Austen, NHMUK 20150177/4; Khasi Hills, coll. Godwin-Austen, NHMUK 20150178/68; Garo Hills, NHMUK leg. W. Robert, coll. Godwin-Austen, NHMUK 20150179/27; Burma, Bassein, coll. Benson 1863, NHMUK 1954.6.2.287/1; Khasi Hills, NHMUK 20150180/1 (mixed sample with *Endothyrella tricarinata*: NHMUK 20150181); Khasi Hills, NHMUK 20150182/3; Burroi Gorge, NHMUK 20150183/2; label not readable, NHMUK 20150185/7; Burrali, NHMUK 20150186/10; Khasi Hills, coll. Godwin-Austen, NHMUK 20150187/65; Khasi Hills, coll. W. Blanford, NHMUK 20150188/3; W. Khasi Hills, coll. Godwin-Austen, NHMUK 20150189/1; N. Khasi, coll. Godwin-Austen, NHMUK 20150190 (more than 100 shells); Khasi Hills, coll. Kennard, NHMUK/20150195/2 (mixed sample with *Endothyrella tricarinata*); Manipur, station 36, Godwin Austen Collection. NHMUK 20150191/87; Manipur, station 48, Godwin Austen Collection. NHMUK 20150192/58; Manipur, station 54, Godwin Austen Collection. NHMUK 20150193/119; Manipur, station 54, Godwin Austen Collection. NHMUK 20150194/89; Indien, leg. Stoliczka, coll. Oberwimmer, NHMW 71640/O/415 (2 shells; mixed sample with *Endothyrella sowerbyi*: NHMW 109252); Khasi Hills, leg. Stoliczka, 1870, NHMW 92588/3; Viaggio in Birmania (= trip to Burma), Shweego, coll. Fea, 1885–1889, NHMW 20034/4; Shwegoo, Birmania, leg. Mission L. Fea 1885–1889, MNHN 2012-27053/3; Khasi Hills, Himalaya, India, coll. Rušnov ex coll. Blume, NHMW 71770/R/13 (2 adult, 1 juv. shells); Ostindien, Pegu, leg. Stoliczka, coll. Edlauer, 477, NHMW 75000/E/4770 (1 shell; mixed sample with *Endothyrella sowerbyi*: NHMW 109253); Darjeeling, Himalaya, India, coll. Rušnov ex coll. Blume, NHMW 71770/R/14 (1 adult, 1 juv. shells; mixed sample with *Endothyrella sowerbyi*: NHMW 71770/R/15); Ostind., coll. Gerstenbrandt, NHMW 83901/G/2745 (2 shells); Pegu, ex coll. Hauer, NHMW 21617/4; Assam, coll. Landauer, NHMW 92594/2; Khasi Hills, Pegu (2 different label were found in the sample), coll. Stoliczka, NHMW 92591/41 (one of them is probably a juvenile *Endothyrella tricarinata*); Khasi, leg. Stoliczka, 1880, NHMW 92592/7; Khasi Hills, leg. Stoliczka, 1880, NHMW 92593/2 (mixed sample with *Endothyrella sowerbyi*: NHMW 109254 and *Endothyrella blanda*: NHMW 109255); East India, leg. Bernardi, Altonaer Museum, coll. O. Semper, ZMH 45908/1; Siam, Altonaer Museum, ZMH 45910/2; Birma, Moulmein, Hinterindien, coll. Krüper 1928, ex coll. Oberwimmer, SMF 118090/2 (mixed sample with *Endothyrella sowerbyi*: SMF 346406); Darjeeling, Himalaya, coll. Jetschin ex coll. Oberwimmer 1899, SMF 118088/1 (mixed sample with *Endothyrella sowerbyi*: SMF 346407); Khasi Hills, coll. Bosch, ex coll. Rolle, SMF 172072/1 (mixed sample with *Endothyrella sowerbyi*: SMF 346408); S-Shan Staaten, Ywathit, Prov. Karenni, a. mittleren Salwen, leg. Michelitz, SMF 150108/3; Indien, coll. Jetschin ex coll. Oberwimmer 1899, SMF 118089/2; Indien, Darjeeling, (alte Schau-sammlung), coll. Kobelt, SMF 150109/2; Khasi-Berge, coll. C. R. Boettger 1904, SMF 118091/1 (Fig. 13B); Assam, coll. Bosch, ex coll. Rolle, SMF 172071/4; Indien, Khasi-Hills, coll. Webb 1928, SMF 150086/2; Indien, Katschar, coll. Möllendorff, Orig. Handb. Pal. Fig. 2092; SMF 150106/4; Goramarah (Ghoramara), Chittagong, coll. Foulon 1936, MNHN 2012-27045/2; no locality, coll. Jousseaume, MNHN 2012-27050/3; no locality, coll. Jousseaume, MNHN 2012-27049/50.

##### Diagnosis.

A very small, sinistral species with very narrow umbilicus, conical dorsal surface, and hairs standing in five rows on the body whorl; palatal plicae more or less straight, the 4th and 5th divided; lamella slightly curved, with short lower and long upper elongation in anterior direction; there are two denticles posteriorly, one above and one below.

##### Measurements

(in mm): D: 8.1–9, H: 4.6–5.1 (n = 3, SMF 172072).

##### Differential diagnosis.

*Endothyrella plectostoma* is similar to *Endothyrella affinis* and *Endothyrella tricarinata* in the narrow umbilicus. All other *Endothyrella* species of similar size have wider umbilicus. *Endothyrella plectostoma* is usually smaller, darker than *Endothyrella affinis*, it has a horizontal, relatively long plica anterior to the lamella, and has the periostracal folds arranged on five spiral line. In contrast, *Endothyrella affinis* lacks the horizontal parietal plica and has four hair rows. Moreover, *Endothyrella plectostoma* has a narrower umbilicus and more elevated spire than *Endothyrella affinis*. See also under *Endothyrella sowerbyi* and *Endothyrella tricarinata* and Table 5.

##### Description of the genitalia

(Figures 22A, 22D–F, 26): Three specimens have been anatomically examined. Collection data: Sikhim, leg. Godwin-Austen, NHMUK 1903.7.1.451. All specimens had 5–6 embryos developing in their uterus. In one specimen no epiphallic differentiation was observed, the vas deferens started from the distal part of the penis (Fig. 22D).

The left ommatophoral retractor passes between penis and vagina. Atrium short; penis relatively short, internally with holes of various sizes; some tiny, rounded calcareous crystals were found in the penis lumen, not directly associated with the holes; this inner structure continued in the epiphallus; penial caecum short, with central thickening; retractor muscle short, it inserts on the proximal end of the penial caecum; epiphallus slightly longer than penis, it enters the proximal penial portion laterally; vas deferens long and slender; vagina approximately as long as the penis, but thicker, curved centrally; vagina with several thick and relatively long muscle fibres attaching it to the body wall and to the diaphragm, especially at its curved portion; vagina internally with longitudinal folds, which are rather sharp, elevated at the curved area of the vagina, and low elsewhere; the gametolytic sac and the diverticulum are aligned in parallel; the gametolytic sac is slightly thicker and shorter; a relatively long part of the spermoviduct was visible distal to the thickened uterus with the developing embryos; the embryo sac contained no visible calcareous granules, which were reported in other plectopylid species (Páll-Gergely and Hunyadi 2013, Páll-Gergely and Asami 2014); albumen gland conspicuously small. The latter trait is largely dependent on the period of the life cycle of the dissected specimen. In the present case, however, three specimens were anatomically examined and all specimens had a small albumen gland.

##### Radula

(Figure 19E–F): Radula elongated, but not very slender, central tooth present, laterals 8, standing in straight lines (perpendicular to the central column); marginals at least 14, staying in oblique rows; central tooth relatively narrow-based triangular, smaller than the endocone of the first lateral, but much larger than the ectocone; laterals bicuspid, endocone oval or narrow-based triangular; marginals tricuspid (the endocone has two cusps); all cusps pointed, the incision between the innermost two cusps is deep; in some cases the three cusps are almost of the same size.

##### Distribution.

Museum samples are labelled from several locations. This species is probably widely distributed in north-eastern India through south-eastern Bangladesh to Bago, the Arakan Hills and in the Kayah State in Burma (Myanmar) (Figure 7). A sample (ZMH 45910) was collected in “Siam” (= Thailand), which is possible because other samples were collected in Myanmar not far from the Thai Border.

##### Remarks.

The name “*prodigium* Benson” probably refers to *Endothyrella plectostoma*. It is a manuscript name, which was mentioned several times in the literature (Godwin-Austen 1875, Tryon 1887, Pilsbry 1894, Gude 1899c), but has never been published formally.

#### Endothyrella robustistriata

Taxon classificationAnimaliaPulmonataPlectopylidae

Páll-Gergely sp. n.

http://zoobank.org/ED59E23B-D8CF-4E09-A439-FE7919DDD5F7

Figures 9K–L , 23C–D

Endothyrella robustistriata 1914b Plectopylis (Endothyra) minor (partim), — Gude: The Fauna of British India…: 76.

##### Type material.

Munipur, Laisen Peak, coll. Godwin-Austen, NHMUK 1903.7.1.3453/1 (holotype, Figure 23D); Naga Hills, Ihang valley, coll. Godwin-Austen, NHMUK 1903.7.1.770/3 paratypes; Naga Hills, coll. Godwin-Austen, NHMUK 1903.7.1.767/3 paratypes (Figure 23C); Lhota Naga Hills, coll. Chennell, NHMUK 1903.7.1.765/4 paratypes.

##### Diagnosis.

A tiny species with elevated spire, smooth ventral side and strongly reticulated dorsal surface; parietal wall with a single lamella, an upper and a lower denticle posteriorly, and a long lower plica which reaches the peristome.

##### Description.

Shell tiny, sinistral, with slightly elevated spire and conical/domed dorsal surface; colour light brown, greenish or yellowish; protoconch consists of approx. 2 whorls, glossy, in some populations (NHMUK 1903.7.1.767, NHMUK 1903.7.1.770, NHMUK 1903.7.1.3453) only the last half whorl has a somewhat ribbed surface, whereas in another population (NHMUK 1903.7.1.765) nearly the whole protoconch is ribbed; dorsal surface of the teleoconch with clearly visible reticulated sculpture dominated by spiral lines; ventral side hairless, smooth, glossy, sometimes with radial growth lines; the ventral and dorsal surface change relatively abruptly above the middle line of the body whorls (from apertural = frontal view); inside the umbilicus there are sharp periostracal folds corresponding with radial ribs; whorls 4.5–4.75 (n = 3), slowly growing, separated by relatively deep suture; umbilicus narrow and deep; apertural lip whitish, thickened, normally not reflexed, or reflexed only near the umbilicus; callus very weak, nearly invisible in case of fresh shells, in case of old, corroded shells it becomes white; aperture without entering fold.

Two opened specimens were observed (NHMUK 1903.7.1.767 and NHMUK 1903.7.1.765). Parietal wall with one rather straight lamella which bends anteriorly; it has both the upper and lower ends elongated anteriorly; two small denticles visible at the posterior side of the lamella, one above and one below; lower plica very long, reaches the peristome; palatal wall with six plicae; first slim and short, the second–fifth plicae horizontal; they do not seem to be divided if we observe through the translucent shell wall, but their middle portion (where the lamella is present on the parietal wall) is much lower; the posterior ends of the middle plicae slightly bent downwards, whereas the anterior parts are straight and horizontal; the last plica is short and slightly curved (Figure 9K–L).

##### Measurements

(in mm): D: 4.1–4.6, H: 2.3–3.5 (n = 2 NHMUK 1903.7.1.765).

##### Differential diagnosis.

*Endothyrella blanda* is similar in shell shape to *Endothyrella robustistriata* sp. n., but is larger, has hairy ventral surface (or if hairs are missing, than hollows are visible indicating the hairs’ positions), and on its dorsal surface the radial lines are dominant. See also under *Endothyrella macromphalus* and *Endothyrella williamsoni* and Table 5.

##### Etymology.

The word *robustistriata* means strongly striated (Latin) which refers to the prominent spiral striae of the new species on the dorsal side of its shell.

##### Type locality.

Munipur, Laisen Peak.

##### Distribution.

The new species is known only from the Naga Hills and Manipur (Figure 11).

#### Endothyrella sowerbyi

Taxon classificationAnimaliaPulmonataPlectopylidae

(Gude, 1899)

Figure 13C–D

Endothyrella sowerbyi 1899a *Plectopylis sowerbyi* Gude: Science Gossip, 5: 239, figs 93a–f. [“Khasi Hills: Assam”].
Endothyrella sowerbyi 1899c Plectopylis (Endothyra) sowerbyi, — Gude: Science Gossip, 6: 148, 149.
Endothyrella sowerbyi 1899d Plectopylis (Endothyra) sowerbyi, — Gude: Science Gossip, 6: 175, 177.
Endothyrella sowerbyi 1914b Plectopylis (Endothyra) sowerbyi, — Gude: The Fauna of British India…: 72, 80–81, figs 30a–f.
Endothyrella sowerbyi 1915 Plectopylis (Endothyra) sowerbyi, — Gude: Records of the Indian Museum, 8: 507, 509.

##### Types.

Khasia Hills, India, NHMUK 1922.8.29.48. (holotype, Figure 13C).

##### Additional material examined.

Indien, leg. Stoliczka, coll. Oberwimmer, NHMW 109252/2 (mixed sample with *Endothyrella plectostoma*: NHMW 71640/O/415); Ostindien, Pegu, leg. Stoliczka, coll. Edlauer, 477, NHMW 109253/7 (mixed sample with *Endothyrella plectostoma*: NHMW 75000/E/4770); Darjeeling, Himalaya, India, coll. Rušnov ex coll. Blume, NHMW 71770/R/15 (3 shells; mixed sample with *Endothyrella plectostoma*: NHMW 71770/R/14); Khasi Hills, leg. Stoliczka, 1880, NHMW 109254 (approx. 70 shells; mixed sample with *Endothyrella plectostoma*: NHMW 92593 and *Endothyrella blanda*: NHMW 109255); Khasi Hills, coll. W. Blanford, NHMUK 1906.2.2.356.4 (3 shells; mixed sample with *Endothyrella plectostoma*: NHMUK 1906.2.2.356.1–3); Darjeeling, 3500', leg. Lister, NHMUK 1907.9.13.11–22/11; Birma, Moulmein, Hinterindien, coll. Krüper 1928, ex coll. Oberwimmer, SMF 346406/2 (mixed sample with *Endothyrella plectostoma*: SMF 118090); Khasi Hills, coll. Bosch, ex coll. Rolle, SMF 346408/5 (mixed sample with *Endothyrella plectostoma*: SMF 172072) (Fig. 13D); Khasi Hills, coll. Jetschin, ex coll. Linter 1893, SMF 118087/1; Darjeeling, Himalaya, coll. Jetschin ex coll. Oberwimmer 1899, SMF 346407/2 (mixed sample with *Endothyrella plectostoma*: SMF 118088).

##### Diagnosis.

A very small, sinistral species with narrow umbilicus (but wider than in the three similar species; *affinis*, *plectostoma*, *tricarinata*), rather domed dorsal surface, and hairs standing in five rows on the body whorl; the hairs are usually missing and the ventral side is with relatively strong radial lines; plication similar to *Endothyrella plectostoma*, but the main anterior parietal plica is missing or weak.

##### Measurements

(in mm): D: 7.8–8.6, H: 4.3–5.0 (n = 3, SMF 346408).

##### Differential diagnosis.

*Endothyrella affinis* is larger, has lighter shell with narrower umbilicus and a weaker sculpture. *Endothyrella sowerbyi* has a wider umbilicus and a thinner peristome than *Endothyrella plectostoma*. Moreover, the spire is lower and the dorsal side is rather domed in *Endothyrella sowerbyi* (conical in *plectostoma*), and the main parietal plica is weaker or missing. See also under *Endothyrella tricarinata* and Table 5.

##### Distribution.

Museum specimens are collected from the Khasi Hills, Darjeeling, and Burma.

##### Remarks.

During the preparation of this revision, *Endothyrella sowerbyi* was handled as the synonym of *Endothyrella plectostoma*, because the only known specimen (the holotype) looked like a juvenile shell of *Endothyrella plectostoma*. The first author recognized that *Endothyrella sowerbyi* is a valid species in the Senckenberg Museum in August, 2015, because of several mixed samples deposited there. Thus, the *Endothyrella plectostoma*/*sowerbyi* sample of the SMF were identified and the *Endothyrella sowerbyi* shells were separated by B. Páll-Gergely. The *Endothyrella plectostoma* samples in the NHM were checked by Jonathan Ablett, whereas those in the NHMW were examined by Zoltán Fehér.

#### Endothyrella tricarinata

Taxon classificationAnimaliaPulmonataPlectopylidae

(Gude, 1897)

Figure 13E–F

Endothyrella tricarinata 1897b Plectopylis plectostoma var. tricarinata Gude: Science Gossip, 3: 275, figs 40a–b. [“Bengal”].
Endothyrella tricarinata 1897g Plectopylis plectostoma var. tricarinata, — Gude: The Journal of Malacology, 6: 45, fig. 2.
Endothyrella tricarinata 1899c Plectopylis (Endothyra) plectostoma var. tricarinata, — Gude: Science Gossip, 6: 148.
Endothyrella tricarinata 1899d Plectopylis (Endothyra) plectostoma var. tricarinata, — Gude: Science Gossip, 6: 176, 177.
Endothyrella tricarinata 1901 Plectopylis plectostoma var. exerta Gude **new synonym**: The Journal of Malacology, 8: 49, figs 5a–d. [“Khasi Hills: Assam”].
Endothyrella tricarinata 1914b Plectopylis (Endothyra) plectostoma var. tricarinata, — Gude: The Fauna of British India…: 83, figs 32a–b. [“Bengal”, “Khasi Hills”].
Endothyrella tricarinata 1914b Plectopylis (Endothyra) plectostoma var. exerta, Gude, The Fauna of British India…: 83–84, figs 33a–d.

##### Types.

Bengal, coll. MacAndrew ex coll. Benson, UMZC 102170 (2 syntypes of Plectopylis plectostoma var. tricarinata, Figure 13E); Khasia Hills, ex Nissor (?), NHMUK 1922.8.29.50. (syntype of Plectopylis plectostoma var. exerta, Figure 13F).

##### Additional material examined.

India, Khasia Hills, K4.30, coll. Rolle, NHMW 50854/2; Assam, Khasia Hills, coll. Bosch, ex coll. Rolle, SMF 172073/3; Assam, Cherrapoonjee, coll. Jetschin, ex coll. Gude 1900, (labelled as syntype, but it is probably not), SMF 118097/1; Assam, coll. Ehrmann ex coll. Schlüter, SMF 150113/1; Khasi Hills, figured in Godwin-Austen (1874), NHMUK 1903.7.1.757 (note that in the original sample it is erroneously 759) (11 specimens under the name *affinis*); Khasi Hills, coll. W. Blanford, NHMUK 1906.1.1.743/2; Khasi Hills, India, Assam, NHMUK 1916.3.16.6–7/2; Khasi Hills, coll. Kennard, NHMUK 20150181/2 (mixed sample with *Endothyrella plectostoma* see NHMUK 20150180); Khasi Hills, NHMUK 20150196/2 (mixed sample with *Endothyrella plectostoma*); Khasi Hills, Assam, ‘Preston’, V.W. MacAndrew Collection (Acc. No.1563), NHMUK 20150197/3; Khasi Hills, Bengal, ‘Rolle, C/R June 03', V.W. MacAndrew Collection (Acc. No.1563), NHMUK 20150198/2; Khasi Hills, India, ‘Rolle, C/R 8/5/13', V.W. MacAndrew Collection (Acc. No.1563), NHMUK 20150199/2.

##### Diagnosis.

A small, sinistral species with narrow umbilicus, conical dorsal surface with strong spiral lines, strongly, densely ribbed surface, and hairs standing in four rows on the body whorl; palatal plicae more or less straight, they are more or less divided; lamella slightly curved, with small denticles on the posterior side (they might fuse to the lamella), and a long upper plica on the anterior side of the lamella.

##### Measurements

(in mm): D: 10.1–10.4, H: 6.3–6.7 (n = 2, SMF 172073).

##### Differential diagnosis.

*Endothyrella affinis* has less shouldered whorls, wider umbilicus, weaker sculpture and it lacks the long horizontal plica anterior to the lamella. *Endothyrella tricarinata* differs from *Endothyrella plectostoma* by the larger size, more conical dorsal surface, narrower umbilicus, the shouldered whorls, the presence of only four rows of hairs, and the stronger sculpture. *Endothyrella sowerbyi* has much weaker dorsal sculpture and has wider umbilicus. See also Table 5.

##### Distribution.

All museum samples were collected from the Khasi Hills and Assam (Figure 11).

##### Remarks.

Two varieties of *Plectopylis plectostoma* have been described under the names Plectopylis plectostoma var. tricarinata and Plectopylis plectostoma var. exerta. Both of them differ from typical *Endothyrella plectostoma* specimens by the more shouldered whorls, and the more conical dorsal side of the shell having stronger spiral lines. No difference between the type specimens of these forms have been found except for the presence (*exerta*) and the absence (*tricarinata*) of hairs. The absence of hairs might be due to the corroded state of the syntypes of *tricarinata*. Although the difference between typical *Endothyrella plectostoma* and typical *tricarinata*/*exerta* shells seem to be minor, we found no intermediate forms, and in some cases we found mixed museum samples which indicate that the shells might have been collected from the same site. This suggest that *Endothyrella plectostoma* and *Endothyrella tricarinata* are distinct species.

#### Endothyrella williamsoni

Taxon classificationAnimaliaPulmonataPlectopylidae

(Gude, 1915)

Figure 17A

Endothyrella williamsoni 1915 Plectopylis (Endothyra) williamsoni Gude: Records of the Indian Museum, 8: 509, Plate 42, figs 1a–d. [“Abor Hills, exact part not indicated”].

##### Types.

Abor Hills, leg. C.F.G. Oakes, R.E., NHMUK 1903.7.1.3087. (5 syntypes, Figure 17A)

##### Diagnosis.

Shell very small, sinistral with narrow umbilicus and conical dorsal surface; shell hairless but densely, finely ribbed and ornamented with low radial periostracal lamellae on the whole shell; callus strong; palatal plicae horizontal, almost straight and thin at their middle; lamella slightly curved; there is long, horizontal plica anteriorly to the lamella, and a short horizontal plica above the long one; additionally, there is a very short upper plica above the lamella, a small denticle posteriorly above, and a long lower plica near the suture which reaches the aperture.

##### Measurements

(in mm): D: 6, H: 3.6–3.7 (n = 2, type series).

##### Differential diagnosis.

*Endothyrella williamsoni* has a more elevated spire than *Endothyrella macromphalus* and *Endothyrella minor*, and has two horizontal parietal plicae anterior to the lamella which are missing in the other two species. The most similar species in terms of shell shape and size to *Endothyrella williamsoni* is *Endothyrella blanda*. The latter species, on the other hand, lacks the two horizontal parietal plicae anterior to the lamella which area characteristic for *Endothyrella williamsoni*. Moreover, *Endothyrella blanda* specimens have seven rows of hairs, whereas *Endothyrella williamsoni* is hairless. *Endothyrella robustistriata* sp. n. is smaller, has stronger dorsal sculpture and lack the main plica which is characteristic for *Endothyrella williamsoni*. See also Table 5.

##### Distribution.

This species is known from the type locality only (Figure 10).

### Species with uncertain identity

#### Plectopylis hanleyi

Taxon classificationAnimaliaPulmonataPlectopylidae

Godwin-Austen, 1879b

Plectopylis hanleyi 1879b *Plectopylis hanleyi* Godwin-Austen: The Annals and Magazine of Natural History, 5 (4): 164. [“Sikkim?”].
Plectopylis hanleyi 1897c *Plectopylis hanleyi*, — Gude: Science Gossip, 4: 11.
Plectopylis hanleyi 1899a *Plectopylis hanleyi*, — Gude: Science Gossip, 5: 240.
Plectopylis hanleyi 1899c Plectopylis (Endothyra) hanleyi, — Gude: Science Gossip, 6: 148.
Plectopylis hanleyi 1899d Plectopylis (Endothyra) hanleyi, — Gude: Science Gossip, 6: 175, 176.
Plectopylis hanleyi 1914b Plectopylis (Endothyra) hanleyi, — Gude: The Fauna of British India…: 73, 77.

##### Original description.

“Shell sinistral, depressedly conoid, openly umbilicated, probably hirsute when young. Sculpture coarse, irregular, transverse ridges. Colour uniform ochraceous. Spire conoidal; apex blunt, smooth. Suture well marked. Whorls six, close-wound, convex. Aperture semicircular, diagonal; peristome somewhat thickened, white, with a thin callus on the parietal margin, not to the extent of a ridge. Size — major diam. 5.5, minor diam. 5.0, alt. 3.0 millims. Parietal vertical lamina simple; palatal plicæ in two rows, four long in front, four short behind, and one basal long. The shell is very distinct; it has somewhat the form of *Plectopylis plectostoma*, but is not so angular on the periphery, while the internal plication is quite different, besides being so very much smaller in size.”

##### Remarks.

In the original description Godwin-Austen (1879b) wrote that the holotype is “in the collection of Mr. Sylvanus Hanley”. In Godwin-Austen’s copy of Gude (1914, page 77), Godwin-Austen has written “In my collection”. The holotype, however, was not found in the collection of the NHM. Only one NHM specimen was found labelled *Plectopylis hanleyi*, and this is annotated with a question mark (“Sikkim, Rarhichu, H. H. Godwin-Austen colln.”). However, this specimen is very similar to the type specimen of *Plectopylis blanda*, and is not identical with the single shell in Godwin-Austen’s (1879b) description, because it has only 4.75 whorls (the holotype of *Plectopylis hanleyi* has six). Moreover, Godwin-Austen (1879b) described the palatal lamellation, whereas the above mentioned specimen is intact, therefore the inner lamellae and plicae could not be observed. Some parts of Hanley’s collection are housed in the Leeds Museum and in the Manchester Museum. The former were contacted and confirmed that the holotype was not deposited there. The catalogue of the type specimens of the Manchester Museum (McGhee 2008) did not list *Plectopylis hanleyi*. Since the holotype of *Plectopylis hanleyi* seems to be lost, and the description is not sufficient to diagnose the species (although it matches with *Endothyrella blanda*), *Plectopylis hanleyi* is considered to be a nomen dubium.

## Results and discussion

Examining all species assigned to *Chersaecia* and *Endothyrella* by Gude (1899c, 1915) revealed that all species formerly assigned to *Endothyrella* by Gude (1899c, 1915) were correctly placed in that genus. The genus *Chersaecia* is, on the other hand, very diverse in terms of shell characters. The type species of *Chersaecia*, *Plectopylis leiophis*, has a finely tuberculated protoconch and an apertural fold (Figure 2). We suggest retaining only those species in *Chersaecia* which share the same features. Consequently, some former *Chersaecia* species (*aborensis*, *andersoni*, *babbagei*, *bedfordi*, *brahma*, *laomontana*, *oglei*, *serica*, *williamsoni*) are excluded from that genus. Most of these species (*aborensis*, *babbagei*, *bedfordi*, *brahma*, *oglei*, *serica*, *williamsoni*) are classified in *Endothyrella* on the basis of the absence of an apertural fold, the ribbed protoconch, the hairs standing in multiple spiral lines and the characters of the armature. *Plectopylis andersoni* and *Plectopylis laomontana* are not assigned to either genus because of the large, keeled shell of *andersoni* with reticulated protoconch and the unique anatomical features of *laomontana* (unpublished information). The systematic position of these two species and the species remained in genus *Chersaecia* will be discussed in separate publications.

The finely ribbed protoconch is considered to be one of the key characters allowing separation of *Chersaecia* and *Endothyrella* species. Dextral *Endothyrella* species however, have “no typical” protoconch: (1) *Endothyrella babbagei* has slightly waved ribs (Figure 6C); (2) *Endothyrella inexpectata* sp. n. has a rather smooth protoconch, some ribbing is only visible on the last half/quarter of whorl (Figure 6F); (3) *Endothyrella serica* has a very finely granulated protoconch with rather irregular ribs/ridges and there is an additional spiral line running close to and parallel with the suture (Figures 6B). However, we see no justification for erecting new (sub) genera for these dextral species yet. Information on their anatomy and molecular evidence may shed light on the importance of these differences as well as the relationship with sinistral *Endothyrella* species.

Based on the ribbed protoconch *Endothyrella* seems to be closely related to *Gudeodiscus*, *Halongella*, *Sicradiscus* and *Sinicola*, and to “*Plectopylis*” *laomontana* and “*Plectopylis*” *andersoni*. Other plectopylid genera without ribs on the protoconch (*Plectopylis*, *Endoplon*, *Chersaecia*) are probably only distantly related. The radula morphology of *Endothyrella* (large central tooth and tricuspid, pointed marginals) are similar to *Sinicola*, *Sicradiscus* and Gudeodiscus (Gudeodiscus) species, whereas Gudeodiscus (Veludiscus) Páll-Gergely 2015 and *Halongella* are characterized by small central teeth and bicuspid or bluntly tricuspid marginals.

## Supplementary Material

XML Treatment for Chersaecia

XML Treatment for Endothyrella

XML Treatment for Endothyrella babbagei

XML Treatment for Endothyrella inexpectata

XML Treatment for Endothyrella oglei

XML Treatment for Endothyrella serica

XML Treatment for Endothyrella aborensis

XML Treatment for Endothyrella affinis

XML Treatment for Endothyrella angulata

XML Treatment for Endothyrella bedfordi

XML Treatment for Endothyrella blanda

XML Treatment for Endothyrella brahma

XML Treatment for Endothyrella dolakhaensis

XML Treatment for Endothyrella fultoni

XML Treatment for Endothyrella macromphalus

XML Treatment for Endothyrella minor

XML Treatment for Endothyrella miriensis

XML Treatment for Endothyrella nepalica

XML Treatment for Endothyrella oakesi

XML Treatment for Endothyrella pinacis

XML Treatment for Endothyrella plectostoma

XML Treatment for Endothyrella robustistriata

XML Treatment for Endothyrella sowerbyi

XML Treatment for Endothyrella tricarinata

XML Treatment for Endothyrella williamsoni

XML Treatment for Plectopylis hanleyi
